# New Visible-Light-Sensitive Dicyanocoumarin- and COUPY-Based Caging Groups with Improved Photolytic Efficiency

**DOI:** 10.3390/molecules30102158

**Published:** 2025-05-14

**Authors:** Marta López-Corrales, Vicente Marchán

**Affiliations:** Departament de Química Inorgànica i Orgànica, Secció de Química Orgànica, Institut de Biomedicina de la Universitat de Barcelona (IBUB), Universitat de Barcelona (UB), Martí i Franquès 1-11, E-08028 Barcelona, Spain

**Keywords:** coumarin, COUPY, photocaging, caging groups, photopharmacology

## Abstract

Photolabile protecting groups (PPGs), also known as caging groups, are valuable tools in photopharmacology. They enable precise control over the release of bioactive compounds from the corresponding caged compounds at a precisely controlled time and place using light of specific wavelengths. This study introduces a novel approach to fine-tuning the photophysical and photochemical properties of visible-light-sensitive dicyanocoumarin- and COUPY-based caging groups by incorporating a phenyl group in a position adjacent to the photolabile bond. Our photoactivation studies with visible light demonstrated that this structural modification slightly improved the photolytic efficiency of both dicyanocoumarin- and COUPY-caged model compounds compared to their methyl-substituted or unsubstituted counterparts. Furthermore, COUPY PPGs were efficiently photoactivated with red light (620 nm) and successfully used to cage two antitumor drugs, chlorambucil and 4-phenylbutyric acid. These findings highlight the potential of phenyl-containing caging groups based on dicyanocoumarin and COUPY scaffolds as versatile platforms for developing new light-activated tools for photopharmacology applications.

## 1. Introduction

Photolabile protecting groups (PPGs), also known as caging groups, can be used to temporarily inactivate the biological activity of a compound of interest by masking essential functionalities through photolabile bonds [1]. Upon exposure to light of a specific wavelength, PPGs undergo photocleavage of these linkages, triggering the release of the bioactive payload at a precisely controlled time and location [2,3,4,5]. The development of new PPGs is crucial to address the limitations of current systems, particularly with respect to the wavelengths required to achieve uncaging with high efficiency. Ideally, PPGs to be used in photopharmacology should be removable by light within the visible or near-infrared (NIR) region of the electromagnetic spectrum to minimize damage to biological systems and allow deeper tissue penetration [6,7]. However, despite recent advances in the field, most of the PPGs described in the literature remain photoactivated by short wavelengths of light. High uncaging quantum yields and rapid photolysis rates are also essential to ensure that the caging groups are removed quickly and completely, thereby maximizing the efficiency of the uncaging process [8,9,10]. Among the visible/NIR-light-sensitive PPGs based on organic chromophores, BODIPY [11,12,13], coumarin [14,15,16,17,18], cyanine [19,20], naphthalene [21,22], and xanthenium [23] derivatives have contributed in recent years to the advancement of photopharmacology, improving the precision and safety of light-activated therapies.

The efficiency of payload photorelease can be evaluated using the so-called “photolytic efficiency” parameter, Φ_Phot_ × *ε*(*λ_irr_*) [1], which is proportional to the amount of the photoreleased substrate. Firstly, *ε*(*λ_irr_*) represents the ability of a given PPG to absorb photons at the irradiation wavelength. This characteristic has been progressively improved in recent years with the development of PPGs exhibiting high absorptivity at longer wavelengths [24]. Secondly, the uncaging quantum yield (Φ_Phot_) is defined as the number of payload molecules released per 100 photons absorbed. Achieving a high Φ_Phot_, which correlates with a more efficient photocleavage, still remains a challenge, as the enhancement of *ε* with strong bathochromic shifts toward the far-red and NIR region has often come at the expense of the photochemical quantum yield [25]. In addition to possessing both high Φ_Phot_ and *ε*(*λ_irr_*), an ideal PPG must fulfill several other important requirements such as aqueous solubility and dark stability, production of non-toxic photo-byproducts upon irradiation, and the ability to release the payload with spatiotemporal precision.

**Scheme 1:**
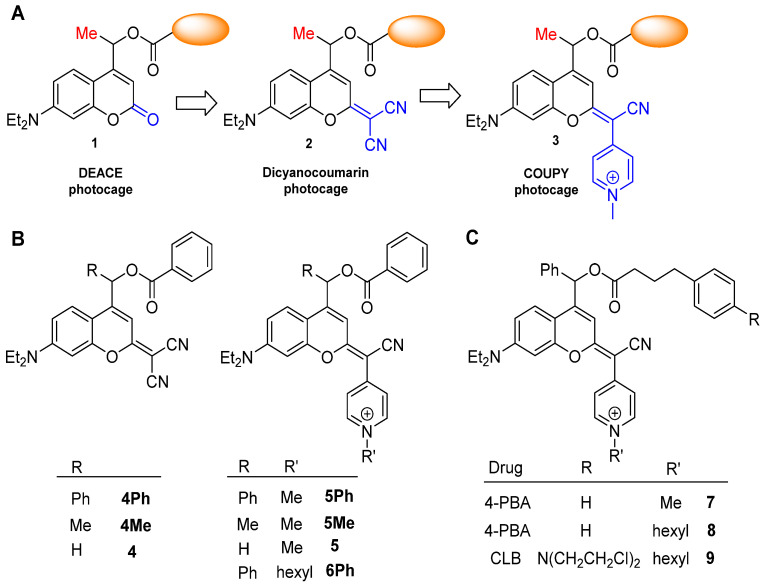
(**A**) Schematic representation of the rational design of dicyanocoumarin- and COUPY-based PPGs. (**B**) Structure of phenyl-containing dicyanocoumarin- (**4Ph**) and COUPY-based (**5Ph**, **6Ph**) caged model compounds synthesized in this work and of previously reported photocages (**4**, **4Me**, **5** and **5Me**). (**C**) COUPY photocages of 4-PBA and CLB bioactive compounds (**7**–**9**) synthesized in this work.

Among the most widely used PPGs, the coumarin scaffold meets most of the criteria for an ideal PPG [26]. Coumarins are relatively easy to synthesize from commercially available precursors and are amenable to structural modifications that facilitate the attachment of the compound to be caged through different types of linkages. The photocleavage mechanism of coumarin-based PPGs is well established and involves a heterolytic bond cleavage upon irradiation, leading to the formation of a tight ion pair [27,28,29]. This ion pair escapes from the solvent cage and reacts with water, resulting in the formation of a coumarin alcohol photoproduct and the corresponding uncaged compound. Thus, stabilizing the tight ion pair represents a promising strategy for enhancing the Φ_Phot_ of coumarin-based PPGs, regardless of the chemical nature of the payload, including conventional coumarins containing the benzopyrone scaffold [30,31]. However, this strategy has been less explored in coumarin-based PPGs removable upon irradiation with long wavelengths of light. In this context, our group reported that the presence of a methyl group adjacent to the photocleavable bond accelerates the photoheterolysis rate of dicyanocoumarin-based PPGs due to the increased stability of the secondary carbocation intermediate formed upon photoheterolysis [15]. As shown in Scheme 1A, replacing the carbonyl group of the lactone in the DEACE photocages (e.g., compound **1**) with a dicyanomethylene group results in red-shifted absorption, allowing uncaging to be carried out with green light (e.g., compound **2**). In two subsequent studies [32,33], we demonstrated the same effect in a new family of caging groups based on COUPY fluorophores [34,35,36], in which one nitrile group in dicyanocoumarin **2** was replaced by *N*-alkyl-4-pyridinium (e.g., compound **3**). Due to their increased push–pull character, COUPY-based photocages can be photoactivated by yellow (560 nm) and red light (620 nm). Moreover, owing to their specific mitochondrial accumulation, COUPY-based PPGs offer a good opportunity for delivering bioactive payloads into mitochondria [33,34], a key subcellular organelle involved in energy production and various functions related to cell growth and death.

Based on these antecedents, in this work, we incorporated a phenyl group adjacent to the photolabile linkage in both dicyanocoumarin-based (**4Ph**) and COUPY-based (**5Ph**, **6Ph**) caging groups to evaluate its influence on the uncaging process (Scheme 1B) This modification aimed to further stabilize the secondary carbocation intermediate generated upon photoheterolysis, compared to compounds containing a methyl group (**4Me** and **5Me**) or lacking any substituent at this position (**4** and **5**). Benzoic acid was selected as a model compound to be caged in all cases, forming an ester bond with the caging groups. Additionally, we demonstrated that phenyl-containing COUPY PPGs can be used to cage bioactive compounds such as 4-phenylbutyric acid (4-PBA) [37] and chlorambucil (CLB) [38] (Scheme 1C), both of which are known for their antitumor properties.

## 2. Results and Discussion

### 2.1. Synthesis and Characterization of Dicyanocoumarin (***4Ph***) and COUPY (***5Ph***, ***6Ph***, ***7***–***9***) Photocages

**Scheme 2:**
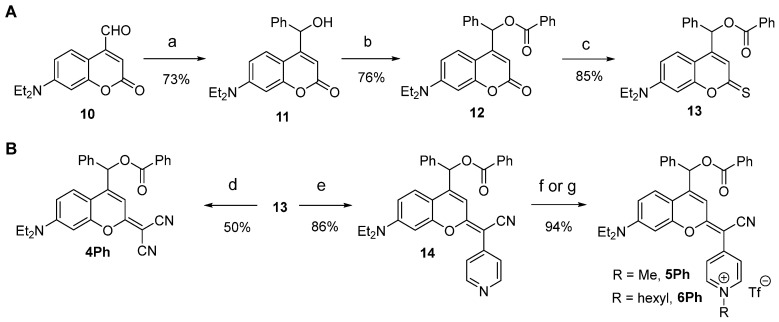
Synthetic scheme followed for the synthesis of dicyanocoumarin (**4Ph**) and COUPY (**5Ph**, **6Ph**) photocages. (**A**) Synthesis route for thiocumarin precursor **13**, and (**B**) synthesis route for final photocages. Reagents and conditions: (a) PhMgBr, THF anh., 0 °C to RT, 1 h; (b) PhCOOH, EDC.HCl, DMAP, DCM, RT, 18 h; (c) LW, toluene, 105 °C, 18 h; (d) malononitrile, NEt_3_, DMF anh., RT, 4 h; (e) (i) 4-pyridylacetonitrile hydrochloride, NaH, ACN anh., RT, 4 h, (ii) AgNO_3_, RT, 2 h; (f) CF_3_SO_3_CH_3_, DCM, RT, 18 h; (g) 1-bromohexane, ACN anh., 60 °C, 18 h.

As shown in Scheme 2, dicyanocoumarin (**4Ph**) and COUPY (**5Ph**, **6Ph**) photocages of benzoic acid were synthesized from coumarin aldehyde **10**, which was obtained from commercially available 7-(*N,N*-diethylamino)-4-methylcoumarin through oxidation with selenium dioxide [15]. First, aldehyde **10** was reacted with phenylmagnesium bromide to afford coumarin alcohol **11**, whose esterification with benzoic acid using EDC as a coupling agent and DMAP as a catalyst provided compound **12**. Thionation of the lactone of coumarin ester **12** with Lawesson’s reagent (LW) afforded thiocoumarin **13**, a key intermediate in the synthetic route. Condensation of thiocoumarin **13** with malononitrile in the presence of triethylamine, followed by treatment with silver nitrate, afforded the dicyanocoumarin photocage **4Ph**. In contrast, COUPY photocages **5Ph** and **6Ph** were obtained in two steps through condensation of thiocoumarin **13** with 4-pyridylacetonitrile followed by *N*-alkylation with methyl trifluoromethanesulfonate or 1-bromohexane, respectively.

**Scheme 3:**
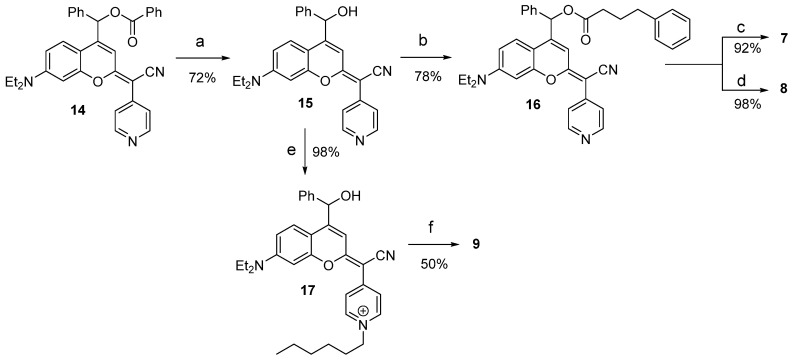
Synthetic scheme followed for the synthesis of COUPY photocages **7**–**9**. (a) NaOH 1 M, ACN/H_2_O 2:1 (*v*/*v*), 35 °C, 18 h. (b) 4-PBA, EDC.HCl, DMAP, DCM/DMF 2:1, RT (*v*/*v*), 18 h; (c) CF_3_SO_3_CH_3_, DCM, RT, 18 h; (d) 1-bromohexane, ACN anh., 60 °C, 18 h; (e) (i) 1-bromohexane, DMF anh., 60 °C, 18 h, (ii) KCl, RT, 4 h; (f) CLB, EDC·HCl, DMAP, DCM, RT, 18 h.

4-PBA- and CLB-containing COUPY photocages **7**–**9** were synthesized from COUPY alcohol **15**, which was obtained by basic hydrolysis of COUPY scaffold **14** (Scheme 3). Esterification with 4-PBA afforded coumarin **16**, whose pyridine was *N*-alkylated with methyl trifluoromethanesulfonate or 1-bromohexane to yield COUPY photocages **7** and **8**, respectively. In contrast, esterification with CLB, using also EDC and DMAP, to obtain COUPY photocage **9**, was performed after *N*-hexylation of coumarin alcohol **15** using 1-bromohexane, followed by treatment with KCl. This latter treatment was implemented to prevent the substitution of one of the chlorine atoms in the nitrogen mustard by the bromide counter-anion. All the compounds were purified by silica column chromatography and fully characterized by high-resolution mass spectrometry (HRMS) and 1D ^1^H and ^13^C{^1^H} NMR spectroscopy. The purity of the compounds was assessed by reversed-phase HPLC-MS (Figure S1).

Interestingly, the 1D ^1^H-NMR spectra of non-alkylated COUPY derivatives (**14**–**16**) in DMSO-*d*_6_ showed two sets of proton signals in ~90:10 ratios, which reproduces the results previously found with other COUPY fluorophores and photocages and accounts for the existence of *E* and *Z* rotamers around the exocyclic carbon–carbon double bond (Figures S2–S4) [32,33]. In all cases, the *E* rotamer was identified as the predominant species. By contrast, only one set of proton signals was observed in the ^1^H-NMR spectra of COUPY-photocages **5Ph**, **6Ph** and **7**–**9**. The presence of a NOESY cross-peak between the H8 of the coumarin backbone and the proton signals of the pyridinium moiety confirmed the *E*-configuration of the exocyclic backbone in these compounds (Figures S5–S9).

### 2.2. Photophysical Characterization of the Compounds

**Figure 1:**
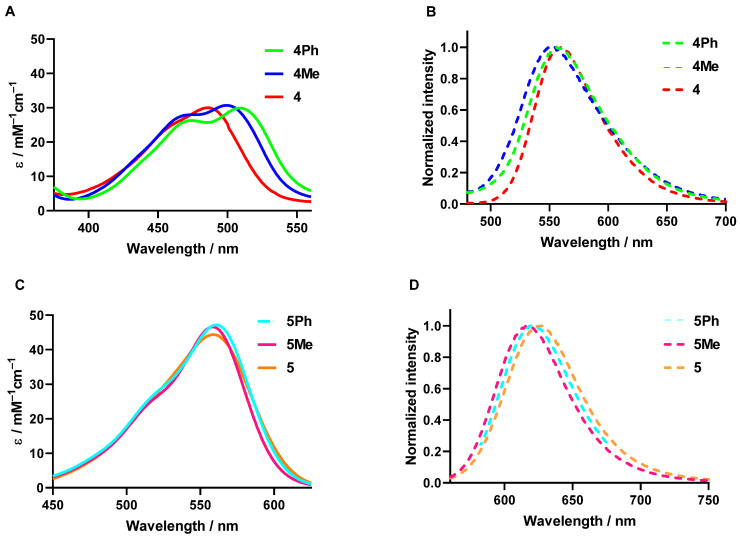
Absorption (**left**) and emission (**right**) spectra of dicyanocoumarin photocages (**4Ph**, **4Me** and **4**) (**A**,**B**) and COUPY photocages (**5Ph**, **5Me** and **5**) (**C**,**D**).

The photophysical properties of the newly synthesized phenyl-containing caged model compounds **4Ph** and **5Ph** were studied in a mixture of PBS and ACN (8:2, *v*/*v*) (Table 1 and Figure 1). These properties were compared with those of previously described compounds, which either have a methyl group adjacent to the photolabile linkage (**4Me** and **5Me**) or lack any substituent at this position (**4** and **5**). This comparison aimed to elucidate the effect of introducing a phenyl group at this position on the spectroscopic properties of the compounds.

Figure 1 illustrates a significant difference in the absorption spectra between dicyanocoumarin and COUPY phenyl-containing photocages. Specifically, **4Ph** absorbs in the green region (λ_max_ = 509 nm), while **5Ph** absorbs in the yellow region (λ_max_ = 561 nm) of the electromagnetic spectrum, with some weak absorption extending beyond 600 nm. This difference is attributed to the enhanced push–pull effect in the COUPY scaffold, achieved by replacing one nitrile group with the electron-withdrawing pyridinium moiety. In both cases, the introduction of a phenyl group causes a slight red-shift with respect to the parent compounds lacking this group, which is more pronounced in the dicyanocoumarin series. However, the emission maxima are blue-shifted in methyl- and phenyl-containing photocages compared to those lacking any substituent (e.g., compare λ_em_ for **5** and **5Me**/**5Ph** in Table 1). As shown in Figure 1, the emission for dicyanocoumarin derivatives appears in the yellow region, whereas COUPY derivatives emit in the far-red region, with maxima ranging from 619 nm (**5Ph**) to 627 nm (**5**). All compounds exhibit relatively high Stokes’ shifts (e.g., 47 nm for **4Ph** and 58 nm for **5Ph**) and have similar molar extinction coefficients to their methyl-containing counterparts (*ε* ≈ 30 mM^−1^cm^−1^ for **4Ph** and **4Me**; *ε* ≈ 47 mM^−1^cm^−1^ for **5Ph** and **5Me**). As summarized in Table 1, fluorescent quantum yield of the dicyanocoumarin derivatives was found to be quite similar regardless of the structural modification around the photolabile bond (Φ_F_ = 0.10 and 0.08 and 0.12 for **4Ph** and **4Me**, respectively). Interestingly, incorporating the phenyl group into the COUPY scaffold resulted in a significant increase in fluorescence emission quantum yield (e.g., Φ_F_ = 0.20 for **5Ph** vs. Φ_F_ = 0.12–0.13 for **5Me** and **5**).

### 2.3. Photolysis Studies of Dicyanocoumarin and COUPY Photocages

**Figure 2:**
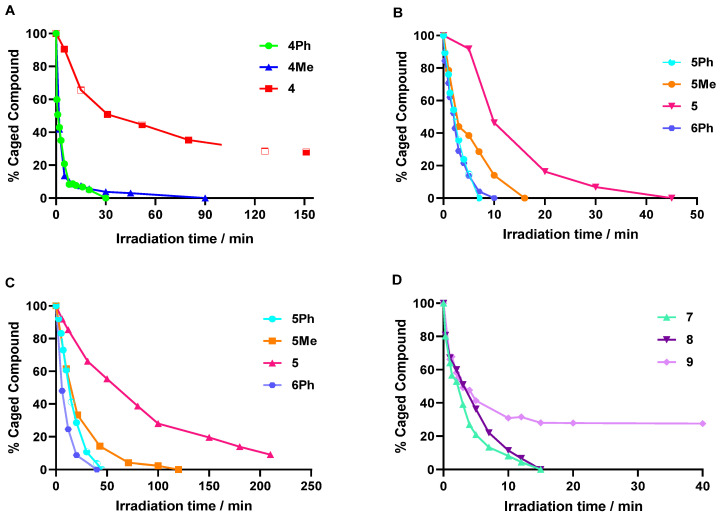
Plot of the temporal evolution of the amount of dicyanocoumarin-caged compounds upon irradiation with green light (505 nm, 100 mW cm^−2^) (**A**), and of COUPY-caged compounds upon irradiation with visible light (470−750 nm range, centered at 530 nm; 150 mW cm^−2^) (**B**,**D**) or red light (620 nm, 130 mW cm^−2^) (**C**). Lines connecting the experimental data points are shown for visual guidance only. All the experiments were performed in an 8:2 (*v*/*v*) mixture of PBS buffer and ACN at 37 °C.

The photoactivation of dicyanocoumarin- and COUPY-caged model compounds (**4Ph**, **5Ph** and **6Ph**) as well as that of the reference compounds (**4**, **4Me**, **5** and **5Me**) was evaluated in a mixture of PBS and ACN (8:2, *v*/*v*) at 37 °C. Photolysis of the COUPY photocages of CLB and 4-BPA (**7**–**9**) was also studied. Prior to irradiation, the compounds were incubated for 2 h at 37 °C to assess their stability in the dark. As shown in Figures S10–S12, no significant degradation was observed in the PBS/ACN mixture, confirming their suitability for further photoactivation studies. The progress of the photolysis under visible light irradiation was monitored by reversed-phase HPLC-MS analysis following the progressive disappearance of the compounds over time (Figures S13–S25). Based on the absorption spectra of the compounds, green light (505 nm, 100 mW cm^−2^) was used for dicyanocoumarins whereas visible light (470−750 nm range, centered at 530 nm; 150 mW cm^−2^) was used for all COUPY photocages. Red light (620 nm; 130 mW cm^−2^) was also used in photolysis studies with COUPY-caged model compounds. As shown in Figure 2, the concentration of all compounds decreased gradually with increasing irradiation time.

Under identical experimental conditions, the release of benzoic acid from compounds **4** and **5** was the slowest in both families compared to their analogs containing either a methyl or phenyl group adjacent to the photolabile bond (e.g., *k*_u_ = 0.009 min^−1^ for **4** and *k*_u_ = 0.184 min^−1^ for **4Ph** under green light irradiation) (Table 2). The incorporation of the phenyl group proved beneficial, as compounds **4Ph** and **5Ph** were photoactivated more rapidly than the methyl-containing analogs (e.g., *k*_u_ = 0.177 min^−1^ for **5Me** and *k*_u_ = 0.384 min^−1^ for **5Ph** under visible light irradiation). Dicyanocoumarin-based PPGs **4Ph** and **4Me** were completely uncaged within 30 min and 90 min, respectively, upon irradiation with green light. In contrast, approximately 40% of compound **4** remained intact after more than 2 h of irradiation (Figure 2A). A similar trend was observed in the COUPY series, where benzoic acid was fully photoreleased in less than 7 min for the phenyl derivative (**5Ph**), compared to 16 min and 45 min for **5Me** and **5**, respectively, using visible light (Figure 2B).

Considering that alkylating the pyridine group of the COUPY scaffold with a hexyl group enhances the mitochondrial uptake compared to the *N*-methyl counterpart [33], we evaluated whether this structural modification would affect the photolysis rate. Notably, the photolysis reaction for compound **6Ph** was completed within a similar timeframe, taking only 10 min. Importantly, benzoic acid was also efficiently photoreleased from all COUPY photocages upon irradiation with red light (620 nm), following the same trend observed with visible light (Figure 2C).

Similarly, 4-PBA- and CLB-containing COUPY photocages (**7**–**9**) were efficiently photoactivated upon irradiation with visible light (Figure 2D). Overall, these results confirmed that the photolysis rate of dicyanocoumarin- and COUPY-based caging groups can be accelerated by incorporating a phenyl group adjacent to the photolabile bond.

As summarized in Scheme S1, three main compounds were identified by HPLC-MS analysis in the photolysis studies for all cases: the photoreleased benzoic acid payload, the expected coumarin alcohol photoproduct, and an additional minor photolytic byproduct. For compounds **4Me** and **5Me**, vinyl coumarin photoproducts were formed via a *β*-elimination reaction (**22**–**23**) from the secondary carbocation intermediate generated upon heterolytic cleavage of the C−O bond. For compounds **4** and **5**, an oxidized coumarin byproduct was identified by MS (**26**–**27**). Similarly, the formation of an oxidized photoproduct was observed (**28**–**30**) in the case of phenyl-containing COUPY photocages (**4Ph**–**6Ph**), as *β*-elimination is not possible in these cases.

Finally, the photolytic efficiency of the uncaging process was determined for dicyanocoumarin photocages (**4Ph**, **4Me** and **4**) upon irradiation with green light, and for COUPY photocages (**5Ph**, **5Me** and **5**) upon irradiation with both visible and red light. This efficiency was calculated as the product of the molar absorption coefficient at the irradiation wavelength and the photolysis quantum yield (Φ_Phot_), derived from the disappearance of photocages upon irradiation (Table 2). In all cases, the incorporation of a phenyl group adjacent to the photolabile bond slightly enhanced the uncaging efficiency, making compounds **4Ph** and **5Ph** the most photolytically efficient caging groups within their respective families. Consistent with the results from the photolysis studies, the Φ_Phot_ of compound **4Ph** was slightly higher than that of the methylated analog (e.g., Φ_Phot_ = 21 × 10^−5^ for **4Ph** vs. Φ_Phot_ = 18 × 10^−5^ for **4Me**), and notably higher compared to the unmethylated analog **4** (Φ_Phot_ = 0.11 × 10^−5^). A similar trend was observed in COUPY photocages, where compound **5Ph** exhibited the highest Φ_Phot_ under visible (Φ_Phot_ = 11 × 10^−5^) and red light (18 × 10^−5^) irradiation.

## 3. Materials and Methods

### 3.1. General Methods

Common chemicals and solvents (HPLC grade or reagent grade quality) were purchased from commercial sources and used without further purification. A hot plate magnetic stirrer, together with an aluminum reaction block of the appropriate size, was used as the heating source in all reactions requiring heat. Aluminum plates coated with a 0.2 mm thick layer of silica gel 60 F_254_ were used for thin-layer chromatography analyses (TLC), whereas column chromatography purification was carried out using silica gel 60 (230–400 mesh). Reversed-phase high-performance liquid chromatography (HPLC) analyses were carried out on a Jupiter Proteo C12 column (150 × 4.6 mm, 90 Å 4 μm, flow rate: 1 mL/min, column 1) or on a XSelect^®^CSH^TM^ Phenyl-Hexyl column (100 × 4.6 mm, 130 Å 3.5 μm, flow rate: 1 mL/min, column 2) using linear gradients of 0.1% formic acid in H_2_O (A) and 0.1% formic acid in ACN (B). NMR spectra were recorded at 25 °C in a 400 MHz spectrometer using the deuterated solvent as an internal deuterium lock. The residual protic signal of chloroform, DMSO or MeOH was used as a reference in ^1^H and ^13^C NMR spectra recorded in CDCl_3,_ DMSO-*d*_6_ and CD_3_OD, respectively. Chemical shifts are reported in part per million (ppm) in the δ scale, coupling constants in Hz and multiplicity as follows: s (singlet), d (doublet), t (triplet), q (quartet), qt (quintuplet), m (multiplet), dd (doublet of doublets), dq (doublet of quartets), br (broad signal), etc. Electrospray ionization mass spectra (ESI-MS) were recorded on a Waters ACQUITY ARC—PDA-QDa system (Milford, MA, USA), and high-resolution (HR) ESI-MS on a G1969A LC/MSD-TOF instrument from Agilent Technologies (Santa Clara, CA, USA).

### 3.2. Synthesis of Coumarin Scaffolds (***11**–**17***)

Compound **11**

A solution of 4-carbaldehyde-7-(*N,N*-diethylamino)coumarin (**10**)^15^ (1.58 g, 6.46 mmol) in anhydrous THF (70 mL) was cooled at 0 °C under Ar atmosphere. Then, a solution of phenylmagnesium bromide in dry THF (7.75 mL, 7.75 mmol) was added dropwise in the dark, and the reaction mixture was stirred for 1 h at room temperature. After the addition of a saturated solution of NH_4_Cl (100 mL), the mixture was brought to room temperature. The solution was extracted four times with ethyl acetate (80 mL), and finally, the combined organic phases were washed with brine (100 mL), dried over anhydrous MgSO_4_ and filtered. After removal of the solvent under vacuum, the product was purified by column chromatography (neutral aluminum oxide, 50–100% DCM in hexane and 0–1.2% MeOH in DCM) to give 1.53 g (73% yield) of a yellow solid.

TLC: Rf (2% MeOH in DCM) 0.50. ^1^H NMR (400 MHz, CDCl_3_) δ (ppm): 7.43 (2H, m), 7.33 (3H, m), 7.27 (1H, d, *J* = 8.0 Hz), 6.45 (2H, d, *J* = 4.0 Hz), 6.42 (1H, dd, *J* = 9.1 Hz *J* = 2.6 Hz), 5.96 (1H, s), 3.34 (4H, q, *J* = 7.3 Hz), 2.71 (1H, br s), 1.15 (6H, t, *J* = 7.3 Hz). ^13^C{^1^H} NMR (101 MHz, CDCl_3_) δ (ppm): 162.7, 156.5, 156.2, 150.2, 140.6, 129.0, 128.7, 127.3, 125.9, 108.4, 106.4, 106.2, 97.7, 72.6, 44.6, 12.4. HRMS (ESI-TOF) *m/z* [M + H]^+^ 324.1594 calcd for C_20_H_22_NO_3_, found 324.1590; analytical HPLC (10 to 100% B over 15 min, column 1) R_t_ = 6.72 min.

Compound **12**

Compound **11** (1.40 g, 4.33 mmol), benzoic acid (793 mg, 6.50 mmol), EDC hydrochloride (1.25 g, 6.50 mmol), and DMAP (793m g, 6.50 mmol) were cooled at 0 °C under an Ar atmosphere and then dissolved in DCM (120 mL). The mixture was stirred at 0 °C for 15 min and then it was allowed to react overnight at room temperature. The solution was washed with saturated NH_4_Cl (200 mL), saturated NaHCO_3_ (1 × 200 mL), and brine (200 mL). The organic layer was dried over anhydrous MgSO_4_ and filtered. After removal of the solvent under vacuum, the product was purified by column chromatography (silica gel, 50–80% DCM in hexane) to give 1.41 g (76% yield) of a yellow solid.

TLC: Rf (DCM) 0.45. ^1^H NMR (400 MHz, CDCl_3_) δ (ppm): 8.10 (2H, m), 7.59 (2H, tt, *J* = 7.3 Hz, *J* = 1.5 Hz), 7.54 (2H, m), 7.46 (2H, m), 7.36 (4H, m), 7.29 (1H, s), 6.49 (1H, s), 6.48 (1H, dd, *J* = 5.3 Hz, *J* = 2.6 Hz), 3.37 (4H, q, *J* = 7.0 Hz), 1.17 (6H, t, *J* = 7.0 Hz). ^13^C{^1^H} NMR (101 MHz, CDCl_3_) δ (ppm): 165.3, 162.3, 156.7, 153.0, 150.6, 137.0, 133.7, 130.0, 129.5, 129.3, 129.2, 128.7, 128.2, 125.8, 108.8, 106.6, 106.1, 97.9, 77.4, 73.2, 44.8, 12.5. HRMS (ESI-TOF) *m/z* [M + H]^+^ 428.1856 calcd for C_27_H_26_NO_4_, found 428.1850; analytical HPLC (60 to 100% B over 15 min, column 1) R_t_ = 11.30 min.

Compound **13**

Lawesson’s reagent (918 mg, 2.27 mmol) was added to a solution of **12** (1.21 g, 2.84 mmol) in toluene (60 mL) under an Ar atmosphere, and the mixture was stirred at 105 °C in the dark overnight. After removal of the solvent under reduced pressure, the product was purified by column chromatography (silica gel, 0–50% DCM in hexane) to give 1.07 g of an orange solid (85% yield). TLC: Rf (Hexane/DCM 1:1) 0.30. ^1^H NMR (400 MHz, CDCl_3_) δ (ppm): 8.11 (2H, d, *J* = 5.6 Hz), 7.59 (1H, t, *J* = 6.0 Hz), 7.52 (2H, d, *J* = 5.6 Hz), 7.46 (3H, d, *J* = 6.4 Hz), 7.36 (3H, m), 7.31 (1H, s), 7.28 (1H, s), 6.66 (1H, d, *J* = 2.0 Hz), 6.57 (1H, dd, *J* = 7.2 Hz, *J* = 2.0 Hz), 3.38 (4H, q, *J* = 5.6 Hz), 1.18 (6H, t, *J* = 5.6 Hz). ^13^C{^1^H} NMR (101 MHz, CDCl_3_) δ (ppm): 197.23, 165.22, 159.46, 150.85, 145.39, 136.90, 133.63, 129.94, 129.28, 129.17, 129.07, 128.61, 127.95, 125.70, 120.38, 110.37, 108.17, 97.41, 72.67, 44.89, 12.41. HRMS (ESI-TOF) *m/z* [M + H]^+^ 444.1628 calcd for C_27_H_26_NO_3_S, found 444.1622; analytical HPLC (60 to 100% B over 15 min, column 1) R_t_ = 13.18 min.

Compound **14**

Thiocoumarin **13** (128 mg, 0.29 mmol) was added to a solution of 4-pyridylacetonitrile hydrochloride (89 mg, 0.58 mmol) and NaH (60% dispersion in mineral oil, 23 mg, 0.58 mmol) in a dry ACN/DCM 1:1 (*v/v*) mixture (20 mL) under an Ar atmosphere and protected from light. After the mixture was stirred for 4 h at room temperature, silver nitrate (123 mg, 0.72 mmol) was added, and the reaction mixture was stirred at room temperature for 2 h under an Ar atmosphere and protected from light. The crude product was evaporated under reduced pressure and purified by column chromatography (silica gel, 0−3% MeOH in DCM) to give 132 mg (86% yield) of an orange solid.

TLC: Rf (5% MeOH in DCM) 0.50. ^1^H NMR (400 MHz, DMSO-*d*_6_) δ (ppm): (major rotamer, *E*) 8.64 (2H, dd, *J* = 6.3, 3.0 Hz), 8.08 (2H, br dd, *J* = 7.1, 1.2 Hz), 7.75 (5H, m), 7.63 (2H, t, *J* = 7.4 Hz), 7.58 (1H, d, *J* = 9.1 Hz), 7.49 (3H, m), 7.39 (1H, s), 7.0 (1H, d, *J* = 0.8 Hz), 6.77 (1H, d, *J* = 2.5 Hz), 6.71 (1H, dd, *J* = 9.1 Hz, 2.5 Hz), 3.49 (4H, q, *J* = 7.3 Hz), 1.16 (6H, t, *J* = 7.3 Hz). ^13^C{^1^H} NMR (101 MHz, DMSO-*d*_6_) δ (ppm): (major rotamer, *E*) 164.9, 163.0, 155.0, 151.1, 150.5, 150.5, 145.4, 140.1, 137.4, 134.5, 129.8, 129.6, 129.55, 129.52, 129.5, 129.4, 129.3, 128.5, 128.3, 126.5, 122.8, 120.9, 119.4, 110.0, 108.7, 106.1, 97.5, 82.9, 73.6, 44.4, 12.9, 12.8. HRMS (ESI-TOF) *m/z* [M + H]^+^ 528.2282 calcd for C_34_H_30_N_3_O_3_, found 528.2282; analytical HPLC (30 to 100% B over 15 min, column 1) R_t_ = 8.58 min. Rotamers ratio *E/Z* 90:10.

Compound **15**

To a solution of coumarin **14** (100 mg, 0.19 mmol) in a 2:1 (*v/v*) mixture of ACN and H_2_O (8 mL), an aqueous solution of sodium hydroxide 1 M (0.57 mL, 0.57 mmol) was added, and the reaction mixture was stirred overnight at 35 °C. After removal of the solvent under reduced pressure, the product was purified by column chromatography (silica gel, 0.25–3.5% MeOH in DCM) to give 58 mg of an orange solid (72% yield). TLC: Rf (7% MeOH in DCM) 0.53. ^1^H NMR (400 MHz, DMSO-*d*_6_) δ (ppm): (major rotamer, *E*) 8.58 (2H, dd, *J* = 6.3, 1.7 Hz), 7.75 (2H, dd, *J* = 6.3, 1.7 Hz), 7.41 (1H, d, *J* = 9.1), 7.35 (2H, m), 7.28 (1H, m), 7.17 (1H, d, *J* = 0.9), 6.68 (1H, d, *J* = 2.2), 6.58 (1H, dd, *J* = 8.8, 2.4 Hz), 6.27 (1H, d, *J* = 4.1 Hz), 5.98 (1H, *br* d, *J* = 3.7 Hz), 3.41 (4H, m), 1.16 (6H, t, *J* = 7.1 Hz). ^13^C{^1^H} NMR (101 MHz, DMSO-*d*_6_) δ (ppm): (major rotamer, *E*) 163.9, 154.8, 154.7, 150.7, 150.6, 150.5, 142.8, 140.5, 129.0, 128.2, 127.7, 126.7, 120.7, 119.8, 109.8, 108.6, 106.9, 97.2, 81.6, 70.8, 55.4, 44.4, 44.2, 12.9, 12.8. HRMS (ESI-TOF) *m/z* [M + H]^+^ 424.2020 calcd for C_27_H_26_O_2_N_3_, found 424.2033; analytical HPLC (10 to 100% B over 15 min, column 1) R_t_ = 8.53 min. Rotamers ratio *E/Z* 88:12.

Compound **16**

A mixture of coumarin **15** (58.2 mg, 0.14 mmol), 4-PBA (34.0 mg, 0.21 mmol), EDC·hydrochloride (39.0 mg, 0.21 mmol), and DMAP (25.1 mg, 0.21 mmol) was cooled at 0 °C under an Ar atmosphere and then dissolved in a 2:1 (*v/v*) mixture of DCM and DMF anhydrous (6 mL). The mixture was stirred at 0 °C for 15 min and then 18 h at room temperature. After removal of the solvent under reduced pressure, the product was purified by column chromatography (silica gel, 50–100% DCM in hexane, and then 0.25–1% MeOH in DCM) to give 61 mg of an orange solid (78% yield). TLC: Rf (5% MeOH in DCM) 0.46. ^1^H NMR (400 MHz, DMSO-*d*_6_) δ (ppm): (major rotamer, *E*) 8.59 (2H, dd, *J* = 6.2, 1.6 Hz), 7.74 (2H, dd, *J* = 6.2, 1.6 Hz), 7.57 (2H, m), 7.40 (5H, m), 7.25 (2H, m), 7.16 (2H, m), 7.09 (1H, s), 6.58 (1H, dd, *J* = 8.8, 2.4 Hz), 6.27 (1H, d, *J* = 4.1 Hz), 5.98 (1H, *br* d, *J* = 3.7 Hz), 3.41 (4H, m), 1.16 (6H, t, *J* = 7.1 Hz). ^13^C{^1^H} NMR (101 MHz, DMSO-*d*_6_) δ (ppm): (major rotamer, *E*) 172.0, 163.0, 154.9, 151.1, 150.5, 145.5, 141.6, 140.1, 137.4, 129.4, 129.4, 128.8, 128.3, 126.5, 126.4, 120.9, 119.5, 110.050, 108.5, 106.1, 97.4, 82.7, 72.8, 44.3, 33.5, 26.8, 26.7, 12.8. HRMS (ESI-TOF) *m/z* [M + H]^+^ 570.2751 calcd for C_37_H_36_O_3_N_3_, found 570.2756; analytical HPLC (30 to 100% B over 15 min, column 1) R_t_ = 7.34 min. Rotamers ratio *E/Z* 90:10.

Compound **17**

To a solution of coumarin **15** (41.6 mg, 0.098 mmol) in anhydrous DMF (5 mL), 1-bromohexane (2.8 mL, 19.6 mmol) was added under an Ar atmosphere, and the reaction mixture was stirred overnight at 60 °C. After removal of the solvent under reduced pressure, the product was purified by column chromatography (silica gel, 0.5–6% MeOH in DCM) to give 51 mg of a pink solid (98% yield). TLC: Rf (10% MeOH in DCM) 0.31. ^1^H NMR (400 MHz, DMSO-*d*_6_) δ (ppm): 8.73 (2H, d, *J* = 5.8 Hz), 8.22 (2H, d, *J* = 5.8 Hz), 7.65 (1H, d, *J* = 6.2 Hz), 7.48 (2H, *br* d, *J* = 5.2 Hz), 7.39 (1H, s), 7.36 (2H, t, *J* = 6.2 Hz), 6.97 (1H, *br* s), 6.82 (1H, dd, *J* = 7.5, 2.1 Hz), 6.45 (1H, d, *J* = 2.0 Hz), 6.18 (1H, d, *J* = 3.5 Hz), 4.46 (2H, t, *J* = 5.8 Hz), 3.50 (4H, m), 1.87 (2H, m), 1.31 (6H, *br* s), 1.13 (6H, t, *J* = 5.7 Hz), 0.87 (3H, m). ^13^C{^1^H} NMR (101 MHz, DMSO-*d*_6_) δ (ppm): 167.6, 156.8, 155., 151.9, 149.1, 143.5, 142.6, 129.1, 128.4, 127.8, 127.4, 121.6, 118.8, 112.0, 107.9, 107.8, 97.1, 80.0, 70.7, 59.3, 44.5, 31.1, 31.0, 25.6, 22.3, 14.3, 12.9. HRMS (ESI-TOF) *m/z* [M + H]^+^ 508.2959 calcd for C_33_H_38_O_2_N_3_, found 508.2972; analytical HPLC (10 to 100% B over 15 min, column 1) R_t_ = 6.32 min.

### 3.3. Synthesis of Photocages (***4Ph***, ***5Ph***, ***7***–***9***)

Compound **4Ph**

A solution of malononitrile (27 mg, 0.40 mmol) and NEt_3_ (0.1 mL, 0.80 mmol) in anhydrous DMF (3 mL) was added to a solution of **14** (118 mg, 0.27 mmol) in anhydrous DMF (5 mL) under an Ar atmosphere. The reaction mixture was stirred in the dark for 4 h. Then, AgNO_3_ (68 mg, 0.40 mmol) was added and stirring was maintained for 2 h. After filtration and removal of the solvent under reduced pressure, the product was purified by column chromatography (silica gel, 0–90% DCM in hexane) to give 94 mg of a pink solid (50% yield). TLC: Rf (DCM) 0.35. ^1^H NMR (400 MHz, CDCl_3_) δ (ppm): 8.10 (2H, d, *J* = 7.8 Hz), 7.60 (1H, br t, *J* = 7.4 Hz), 7.44 (8H, m), 7.31 (1H, s), 7.03 (1H, s), 6.57 (2H, m), 3.40 (4H, q, *J* = 7.2 Hz), 1.20 (6H, t, *J* = 7.2 Hz). ^13^C{^1^H} NMR (101 MHz, CDCl_3_) δ (ppm): 171.9, 165.2, 155.4, 151.5, 149.6, 136.5, 133.8, 129.9, 129.5, 129.2, 129.0, 128.8, 127.8, 126.2, 114.6, 113.9, 110.7, 107.0, 105.8, 97.4, 72.8, 55.8, 44.9, 12.5. HRMS (ESI-TOF) *m/z* [M + H]^+^ 476.1969 calcd for C_30_H_26_O_3_N_3_, found 476.1984; analytical HPLC (60 to 100% B over 15 min, column 1) R_t_ = 12.59 min.

Compound **5Ph**

Methyl trifluoromethanesulfonate (18 μL, 0.15 mmol) was added to a solution of compound **14** (40 mg, 0.076 mmol) in DCM (5 mL) under an Ar atmosphere. The mixture was stirred overnight at room temperature and protected from light. After removal of the solvent under reduced pressure, purification by column chromatography (silica gel, 0−3% MeOH in DCM) afforded 51 mg (94% yield) of a pink solid. TLC: Rf (1% MeOH in DCM) 0.45. ^1^H NMR (400 MHz, DMSO-*d*_6_) δ (ppm): 8.65 (2H, d, *J* = 8.0 Hz), 8.20 (2H, d, *J* = 8.0 Hz), 8.09 (2H, m), 7.74 (4H, m), 7.60 (2H, m), 7.44 (4H, m), 7.21 (1H, s), 6.98 (1H, d, *J* = 2.7 Hz), 6.89 (1H, dd, *J* = 9.3, 2.7 Hz), 4.22 (3H, s), 3.51 (4H, q, *J* = 6.7 Hz), 1.15 (6H, t, *J* = 6.7 Hz). ^13^C{^1^H} NMR (101 MHz, DMSO-*d*_6_) δ (ppm): 167.5, 165.2, 155.9, 152.6, 151.2, 149.7, 143.2, 136.6, 133.8, 129.9, 129.5, 129.3, 129.1, 128.8, 127.8, 126.2, 121.6, 117.9, 112.0, 107.9, 107.5, 97.5, 80.9, 73.0, 46.5, 45.1, 29.7, 12.4. HRMS (ESI-TOF) *m/z* [M]^+^ 542.2438 calcd for C_35_H_32_O_3_N_3_, found 542.2457; analytical HPLC (30 to 100% B over 15 min, column 1) R_t_ = 7.85 min.

Compound **6**

To a solution of **14** (60.7 mg, 0.11 mmol) in ACN anhydrous (5 mL), 1-bromohexane (0.8 mL, 5.75 mmol) was added under an Ar atmosphere, and the reaction mixture was stirred overnight at 60 °C. After removal of the solvent under reduced pressure, the product was purified by column chromatography (silica gel, 0−5% MeOH in DCM) to give 75 mg (94% yield) of a pink solid. TLC: Rf (1% MeOH in DCM) 0.47. ^1^H NMR (400 MHz, DMSO-*d*_6_) δ (ppm): 8.74 (2H, d, *J* = 7.8 Hz), 8.21 (2H, d, *J* = 7.8 Hz), 8.09 (2H, m), 7.75 (4H, m), 7.59 (2H, m), 7.45 (4H, m), 7.22 (1H, s), 7.0 (2H, d, *J* = 2.4 Hz), 6.90 (1H, dd, *J* = 6.9, 2.6 Hz), 4.47 (2H, t, *J* = 7.1 Hz), 3.53 (4H, q, *J* = 7.2 Hz), 1.88 (2H, m), 1.29 (4H, *br* s), 1.15 (6H, t, *J* = 7.2 Hz). ^13^C{^1^H} NMR (101 MHz, DMSO-*d*_6_) δ (ppm): 167.5, 165.2, 156.1, 152.6, 151.1, 149.5, 142.6, 136.7, 133.8, 129.9, 129.4, 129.2, 129.1, 128.8, 127.8, 126.1, 122.1, 118.2, 112.0, 108.0, 107.6, 98.4, 81.1, 73.0, 60.1, 45.4, 31.6, 31.4, 31.1, 25.8, 22.4, 13.9, 12.7. HRMS (ESI-TOF) *m*/*z* [M]^+^ 612.3221 calcd for C_40_H_42_O_3_N_3_, found 612.3235; analytical HPLC (30 to 100% B over 15 min, column 1) R_t_ = 9.59 min.

Compound **7**

Methyl trifluoromethanesulfonate (14 μL, 0.11 mmol) was added to a solution of compound **16** (31.1 mg, 0.054 mmol) in DCM (3 mL) under an Ar atmosphere. The mixture was stirred overnight at room temperature and protected from light. After removal of the solvent under reduced pressure, purification by column chromatography (silica gel, 0−6.5% MeOH in DCM) afforded 36.4 mg (92% yield) of a pink solid. TLC: Rf (10% MeOH in DCM) 0.48. ^1^H NMR (400 MHz, CD_3_OD) δ (ppm): 8.51 (1H, d, *J* = 7.6 Hz), 8.25 (1H, d, *J* = 7.6 Hz), 7.59 (1H, d, *J* = 9.2 Hz), 7.51 (2H, m), 7.40 (3H, m), 7.21 (4H, m), 7.11 (3H, m), 6.94 (1H, d, *J* = 2.6 Hz), 6.82 (1H, dd, *J* = 9.2, 2.6 Hz), 4.21 (3H, s), 3.55 (4H, q, *J* = 7.1 Hz), 2.63 (2H, t, *J* = 7.8 Hz), 2.51 (2H, m), 1.99 (2H, m), 1.23 (6H, t, *J* = 7.1 Hz). ^13^C{^1^H} NMR (101 MHz, CD_3_OD): δ (ppm) 173.5, 168.8, 157.2, 153.7, 152.8, 150.8, 144.8, 142.5, 138.3, 130.4, 130.2, 129.5, 129.4, 129.1, 127.9, 127.0, 122.6, 119.0, 113.0, 108.8, 108.6, 97.8, 81.9, 74.1, 46.9, 45.8, 36.0, 34.4, 27.8, 12.7. HRMS (ESI-TOF) *m/z* [M]^+^ 584.2908 calcd for C_38_H_38_O_3_N_3_, found 584.2909; analytical HPLC (10 to 100% B over 15 min, column 1) R_t_ = 6.80 min.

Compound **8**

To a solution of coumarin **16** (20 mg, 0.035 mmol) in anhydrous ACN (4 mL), 1-bromohexane (0.5 mL, 3.51 mmol) was added under an Ar atmosphere, and the reaction mixture was stirred overnight at 60 °C. After removal of the solvent under reduced pressure, the product was purified by column chromatography (silica gel, 0.5–6% MeOH in DCM) to give 25.2 mg of a pink solid (98% yield). TLC: Rf (10% MeOH in DCM) 0.44. ^1^H NMR (400 MHz, CD_3_OD) δ (ppm): 8.60 (2H, d, *J* = 7.4 Hz), 8.28 (2H, d, *J* = 7.4 Hz), 7.60 (1H, d, *J* = 7.3 Hz), 7.52 (2H, m), 7.39 (3H, m), 7.21 (4H, m), 7.11 (2H, m), 6.97 (1H, d, *J* = 2.6 Hz), 6.83 (1H, dd, *J* = 9.3, 2.6 Hz), 4.44 (2H, t, *J* = 7.5 Hz), 3.56 (4H, q, *J* = 6.0 Hz), 2.63 (2H, t, *J* = 7.6 Hz), 2.51 (2H, m), 1.99 (2H, m), 1.38 (4H, m), 1.22 (6H, t, *J* = 6.0 Hz), 0.92 (5H, m). ^13^C{^1^H} NMR (101 MHz, CD_3_OD): δ (ppm) 173.5, 168.8, 157.2, 153.7, 152.9, 151.0, 143.9, 142.5, 138.3, 130.4, 130.2, 129.5, 129.4, 129.1, 127.9, 127.0, 122.7, 119.0, 113.1, 108.8, 108.7, 97.9, 82.0, 74.1, 61.1, 45.9, 36.0, 34.4, 32.7, 32.3, 32.2, 27.8, 26.9, 23.7, 23.5, 14.4, 14.3, 12.7. HRMS (ESI-TOF) *m/z* [M]^+^ 654.3690 calcd for C_43_H_48_O_3_N_3_, found 654.3693; analytical HPLC (10 to 100% B over 15 min, column 1) R_t_ = 6.77 min.

Compound **9**

A mixture of coumarin **17** (26.5 mg, 0.048 mmol), CLB (22.2 mg, 0.073 mmol), EDC·hydrochloride (14 mg, 0.073 mmol) and DMAP (9 mg, 0.073 mmol) was cooled at 0 °C under an argon atmosphere and then dissolved in DCM (6 mL). The mixture was stirred at 0 °C for 15 min and then 18 h at room temperature. After removal of the solvent under reduced pressure, the product was purified by column chromatography (silica gel, 0.5–5% MeOH in DCM) to give 20 mg of purple solid (50% yield). TLC: Rf (10% MeOH in DCM) 0.34. ^1^H NMR (400 MHz, CD_3_OD) δ (ppm): 8.62 (2H, d, *J* = 7.4 Hz), 8.32 (2H, d, *J* = 7.4 Hz), 7.60 (1H, d, *J* = 9.3 Hz), 7.52 (2H, m), 7.41 (3H, m), 7.25 (1H, d, *J* = 1.0 Hz), 7.20 (1H, s), 7.02 (2H, d, *J* = 8.5 Hz), 6.97 (1H, *J* = 2.0 Hz), 6.86 (1H, dd, *J* = 9.3, 2.5 Hz), 6.62 (2H, d, *J* = 8.9 Hz), 4.43 (2H, t, *J* = 7.5 Hz), 3.62 (12H, m), 2.56 (2H, t, *J* = 7.3 Hz), 2.49 (2H, td, *J* = 7.0, 3.2 Hz), 2.00 (4H, m), 1.40 (4H, m), 1.23 (6H, t, *J* = 7.1 Hz), 0.94 (3H, m). ^13^C{^1^H} NMR (101 MHz, CD_3_OD): δ (ppm) 172.2, 167.4, 155. 8, 152.3, 151.4, 149.6, 144.6, 142.5, 137.0, 129.8, 129.3, 129.0, 128.8, 128.5, 127.8, 127.8, 126.5, 124.9, 121.3, 117.6, 112.0, 111.7, 107.4, 107.3, 96.5, 80.6, 72.5, 59.7, 53.4, 53.1, 44.5, 40.2, 33.5, 33.0, 30.9, 30.8, 26.5, 25.5, 22.1, 12.9, 11.3. HRMS (ESI-TOF) *m/z* [M + H]^+^ 793.3646 calcd for C_47_H_55_O_3_N_4_Cl_2_, found 793.3644; analytical HPLC (30 to 100% B over 15 min, column 1) R_t_ = 8.35 min.

### 3.4. Photophysical Characterization of the Compounds

Absorption spectra were recorded in a Jasco V-730 spectrophotometer (Jasco, Tokyo, Japan) at room temperature. Molar absorption coefficients (ε) were determined by direct application of Beer–Lambert’s law, using solutions of the compounds in an 8:2 (*v*/*v*) mixture of PBS buffer and ACN with concentrations about 10^−6^ M. Emission spectra were registered in a Photon Technology International (PTI) fluorimeter (Ediso, NJ, USA). The standard fluorophores used for determining fluorescence quantum yields (Φ_F_) were fluorescein and cresyl violet. Fluorescein, with a quantum yield reference value (Φ_F;Ref_) of 0.92, was dissolved in 0.1 M aqueous sodium hydroxide. This fluorophore was used for dicyanocoumarin photocages. Cresyl violet, with Φ_F;Ref_ = 0.54, was dissolved in ethanol and used for COUPY photocages. The concentrations were adjusted such that the absorption was around 0.04 at the excitation wavelength for dicyanocoumarin photocages (λ_Ex_ = 460 nm) or around 0.1 at the excitation wavelength for COUPY photocages (λ_Ex_ = 540 nm). Then, optically matched solutions of the compounds and the corresponding reference fluorophore were excited, and the fluorescence spectra were recorded. Φ_F_ values were calculated using the following Equation (1):

(1) $$\Phi_{F;Sample}=\frac{{Area}_{Sample}}{{Area}_{Ref}}\times {\frac{{Abs}_{Ref}}{{Abs}_{Sample}}\times \left(\frac{{ƞ}_{Sample}}{{ƞ}_{Ref}}\right)}^{2}\times \Phi_{F;ref}$$

where Area_Sample_ and Area_Ref_ are the integrated fluorescence for the sample and the reference, Abs_Sample_ and Abs_Ref_ are the absorbance registered at excitation wavelength, and ƞ_Sample_ and ƞ_Ref_ are the refractive indexes of sample and reference solutions, respectively.

### 3.5. Irradiation Experiments

Photolysis studies were performed at 37 °C in a custom-built irradiation setup from Microbeam (Barcelona, Spain), which includes a cuvette, thermostated cuvette holder, and mounted high-power LED of wide range visible light (470–750 nm range, centered at 530 nm, 150 mW cm^−2^), red light (620 nm, 130 mW cm^−2^) or green light (505 nm, 100 mW cm^−2^). In a typical experiment, the cuvette containing 1.5 mL of a solution of the caged compound (20 μM) and 4-*N,N*-dimethylaminopyridine (internal standard, 20 μM) in an 8:2 (*v/v*) mixture of PBS buffer and ACN was placed in front of the light source and irradiated for the indicated times while constantly stirred. At each time point, samples were taken and analyzed by reversed-phase HPLC-ESI MS with column 1 or 2 (see Section 2.1) by using linear gradients of 0.1% formic acid in H_2_O (A) and 0.1% formic acid in ACN (B).

### 3.6. Uncaging Quantum Yield Determination

The uncaging quantum yields (Φ_Phot_) of dicyanocoumarin photocages (**4Ph**, **4Me** and **4**) at 505 nm and COUPY photocages (**5Ph**, **5Me** and **5**) at 530 nm or 620 nm were determined by actinometry using [1,2-bis(2,4-dimethyl-5-phenyl-3-thienyl)perfluorocyclo-pentene] (DAE) as a visible-light actinometer [39]. DAE covers a wide range of the visible region between 480 and 620 nm, being suitable for calculating the photon flux with the three sources. Photon fluxes calculated from DAE actinometry, together with the uncaging photolysis parameters obtained for each photocage, enabled the determination of *Φ*_Phot_ values (see Supporting Information for further details).

## 4. Conclusions

In summary, this work describes a novel approach to enhancing the photophysical and photochemical properties of visible-light-sensitive dicyanocoumarin- and COUPY-based caging groups by incorporating a phenyl group adjacent to the photolabile bond in the coumarin backbone. Dicyanocoumarin- (**4Ph**) and COUPY-based (**5Ph**) caged model compounds were synthesized in a few steps with high yields from commercially available precursors, and their photophysical and photochemical properties were characterized.

Photoactivation studies with visible light revealed that this structural modification improved the photolytic efficiency of both dicyanocoumarin- and COUPY-caged model compounds compared to their methyl-substituted (**4Me** and **5Me**) or unsubstituted (**4** and **5**) counterparts. This was accompanied by a bathochromic shift, enhanced molar absorptivity, and increased fluorescence quantum yields. Additionally, COUPY PPGs were efficiently photoactivated with red light (620 nm) and successfully used to cage two antitumor drugs, 4-phenylbutyric acid (**7**–**8**) and chlorambucil (**9**).

These findings highlight the potential of phenyl-containing caging groups based on dicyanocoumarin and COUPY scaffolds as versatile platforms for developing new light-activated tools for photopharmacology applications. This approach also paves the way for incorporating this modification into other classes of caging groups based on organic chromophores.

## Figures and Tables

**Table 1:** Photophysical properties of dicyanocoumarin-caged (**4Ph**, **4Me** and **4**) and COUPY-caged (**5Ph**, **5Me** and **5**) model compounds.

	Absorption		Emission		
Compound	λ_max_ (nm) ^a^	*ε*(λ_max_) (mM^−1^ cm^−1^) ^b^	λ_em_ (nm) ^c^	Stokes’ Shift (nm) ^d^	Φ_F_ ^e^
**4Ph**	509	30.0	556	47	0.10
**4Me**	498	30.7	555	57	0.08
**4**	486	30.0	560	74	0.09
**5Ph**	561	47.2	619	58	0.20
**5Me**	558	46.6	616	58	0.13
**5**	557	44.2	627	70	0.12

^a^ Wavelength of the absorption maximum. ^b^ Molar absorption coefficient at λ_max_. ^c^ Wavelength of the emission maximum upon excitation at 20 nm below λ_max_. ^d^ Stokes’ shift. ^e^ Fluorescence quantum yield.

**Table 2:** Photochemical parameters for COUPY-caged model compounds ^a^.

Compound	Source (nm)	*k_u_* (min^−1^) ^b^	Φ_Phot_ [×10^−5^] ^c^	*ε*(λ_irr_) [mM^−1^ cm^−1^] ^d^	Φ_Phot_ × *ε*(*λ_irr_*) [M^−1^cm^−1^] ^e^
**4Ph**	505	0.184	22.0	29.7	4.60
**4Me**	505	0.069	18.2	29.6	4.12
**4**	505	0.009	0.13	12.3	0.03
**5Ph**	530	0.384	11.0	30.3	3.57
	620	0.086	19.4	2.79	0.54
**5Me**	530	0.177	8.68	29.8	2.59
	620	0.037	14.7	1.53	0.23
**5**	530	0.099	3.91	31.9	1.25
	620	0.011	1.57	2.3	0.04

^a^ Irradiation was performed with green (505 nm, 100 mW cm^−2^), visible (470−750 nm range, centered at 530 nm; 150 mW cm^−2^), or red (620 nm; 130 mW cm^−2^) light in an 8:2 (*v*/*v*) mixture of PBS buffer and ACN. ^b^ Uncaging first-order rate constant. ^c^ Uncaging quantum yields were determined from the degradation of the compounds by actinometry. ^d^ Molar absorption coefficient at the irradiation wavelength (505, 530 or 620 nm). ^e^ Photolytic efficiency at the irradiation wavelength.

## Data Availability

Data are contained within the article and Supplementary Materials.

## Supplementary Materials

The following supporting information can be downloaded at: https://www.mdpi.com/article/10.3390/molecules30102158/s1, Additional figures and material from irradiation experiments, uncaging quantum yield determination and copies of ^1^H, 2D-NOESY and ^13^C{1H} NMR and HRMS spectra of the synthesized compounds. References [39,40,41,42] are cited in the supplementary materials.
