# Supplementary material for: New Visible-Light-Sensitive Dicyanocoumarin- and COUPY-Based Caging Groups with Improved Photolytic Efficiency
Source: Molecules. 2025 May 14;30(10):2158. doi: 10.3390/molecules30102158 (PMC12114166; doi:10.3390/molecules30102158)
Supplement: Supplementary file 1 [file molecules-30-02158-s001.zip › molecules-3624777-supplementary.pdf]

SUPPORTING INFORMATION

New visible-light sensitive dicyanocoumarin- and COUPY-based caging groups with improved photolytic efficiency

Marta López-Corrales, Vicente Marchán*

Departament de Química Inorgànica i Orgànica, Secció de Química Orgànica, Universitat de Barcelona (UB), and Institut de Biomedicina de la Universitat de Barcelona (IBUB), Martí i Franquès 1-11, E-08028 Barcelona, Spain. Email: vmarchan@ub.edu

Table of contents

1.-Reversed-phase HPLC analysis of coumarin photocages (**4Ph-6Ph, 7-9**) S3

2.- 2D NMR characterization of COUPY scaffolds (**14-16**) S4

3.- 2D NMR characterization of COUPY photocages (**5Ph, 6Ph, 7-9**) S7

4.- Irradiation experiments S12

5.- Determination of uncaging quantum yield (Φ_{Phot}) using DAE actinometry protocol S29

6.- ^{1}H and ^{13}C spectra and HR ESI-MS of the compounds S31

7.- References S57

1.- Reversed-phase HPLC analysis of coumarin photocages (4Ph-6Ph, 7-9).

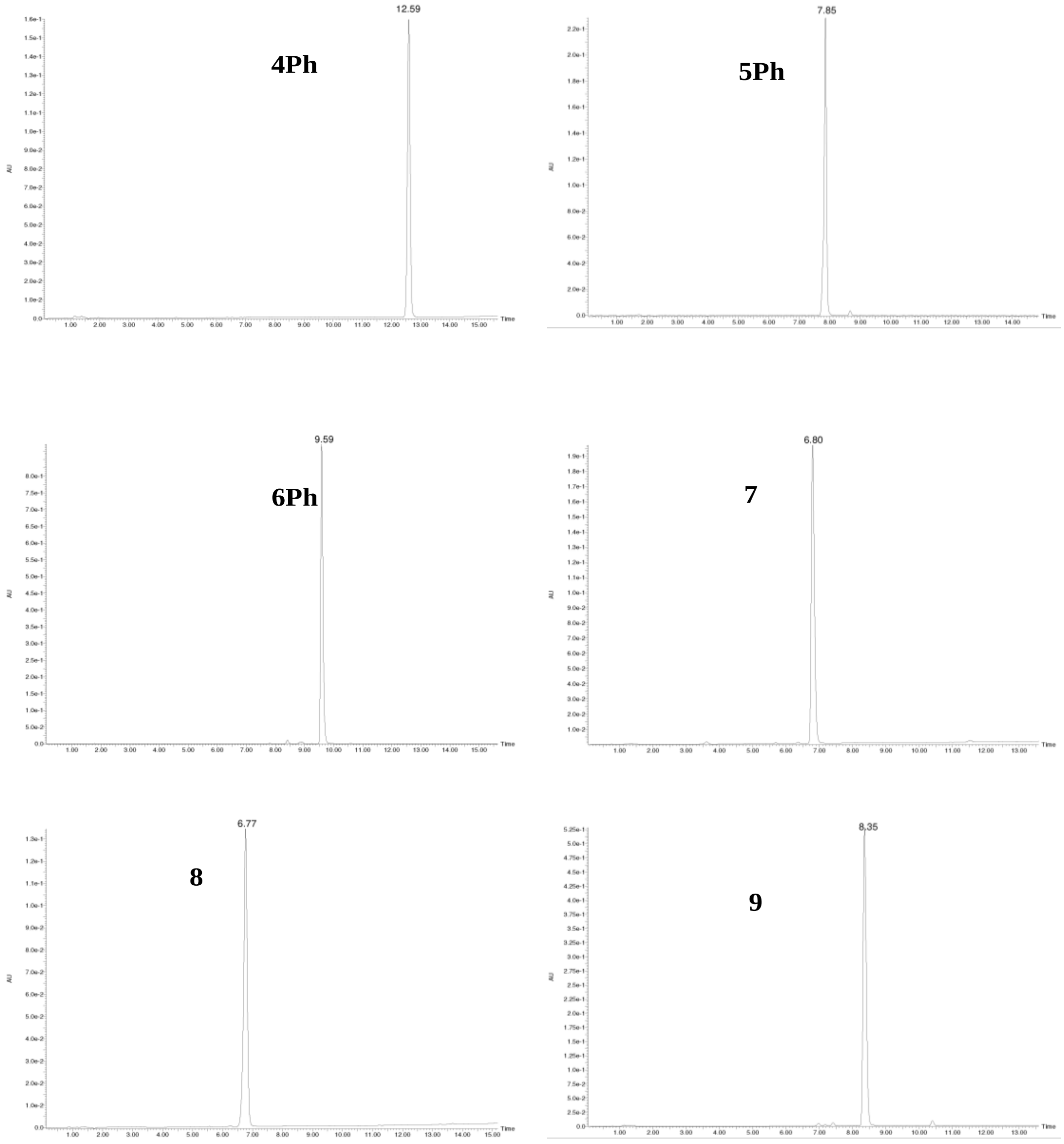

Figure S1. Reversed-phase HPLC traces at 260 nm of dicyanocoumarin- and COUPY-caged compounds using column 1.

2.- 2D NMR characterization of COUPY scaffolds (14-16)

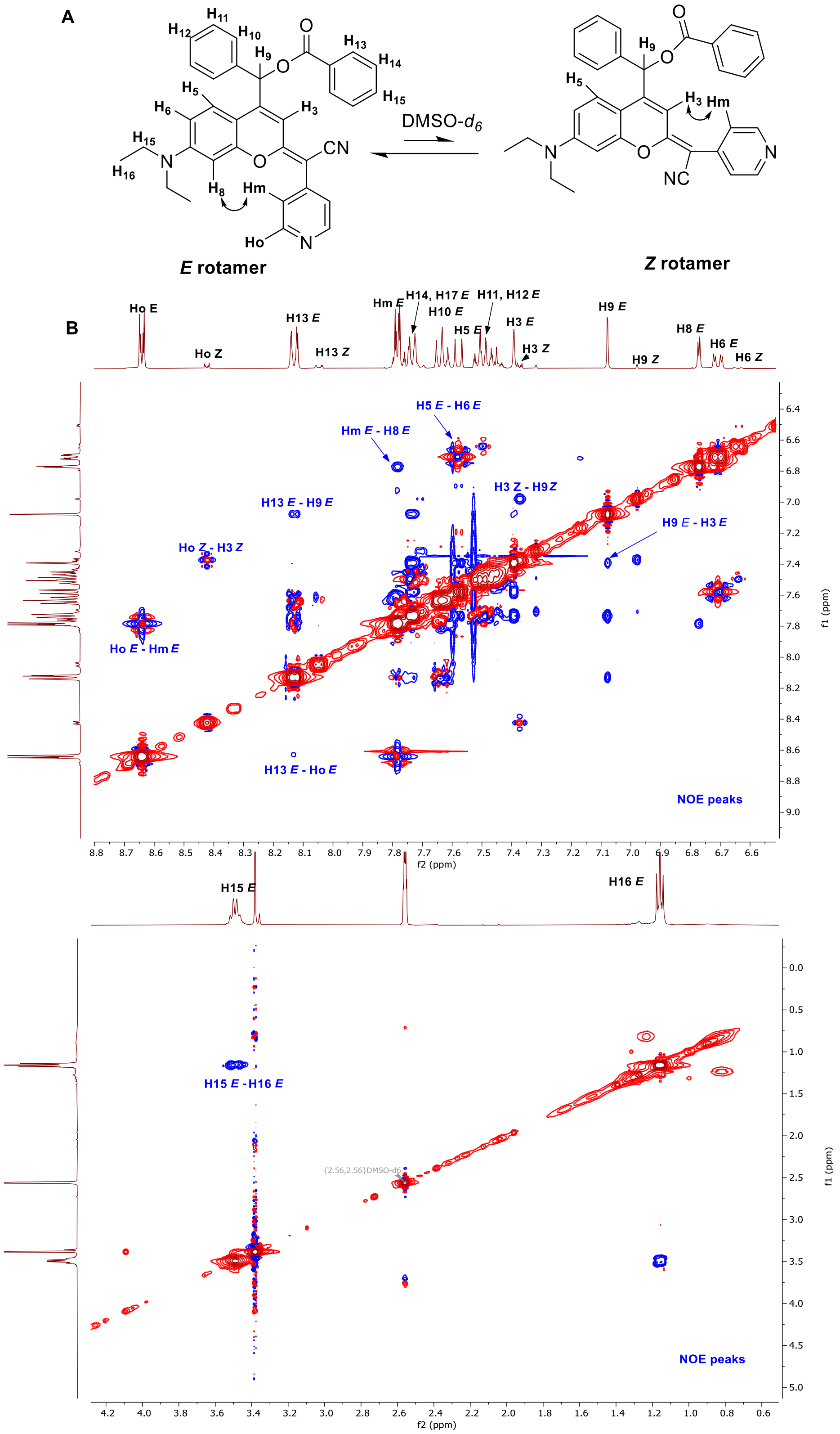

Figure S2. A) Structure of *E* and *Z* rotamers of coumarin **14** with some diagnostic NOE cross-peaks indicated. B) Expansions of the NOESY spectrum (DMSO-d_6, 298 K) of coumarin **14** showing some characteristic NOE cross-peaks.

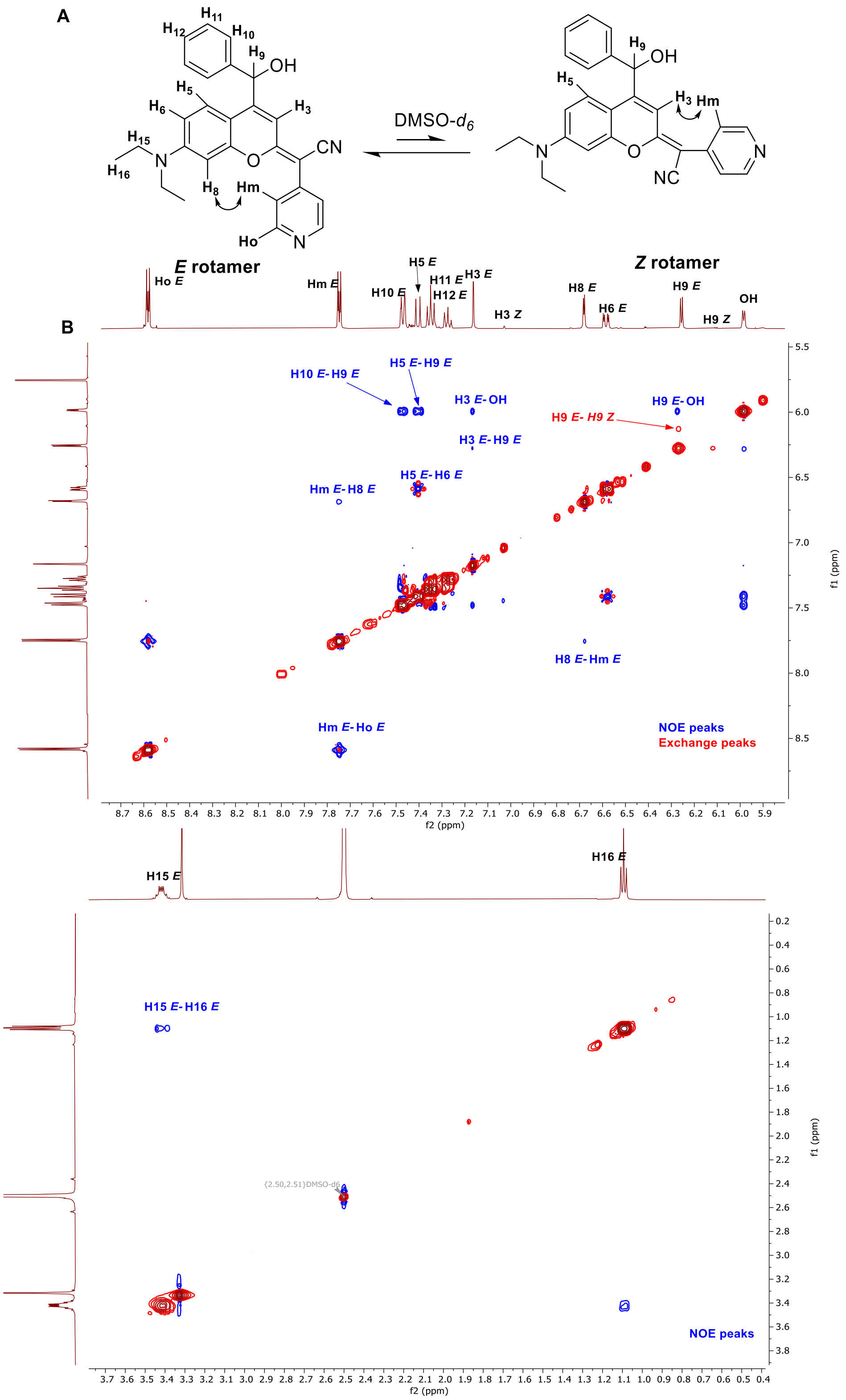

Figure S3. A) Structure of *E* and *Z* rotamers of coumarin **15** with some diagnostic NOE cross-peaks indicated. B) Expansions of the NOESY spectrum (DMSO-d_6, 298 K) of coumarin **15** showing some characteristic NOE cross-peaks and exchange cross-peaks between rotamer resonances of the same sign as the diagonal.

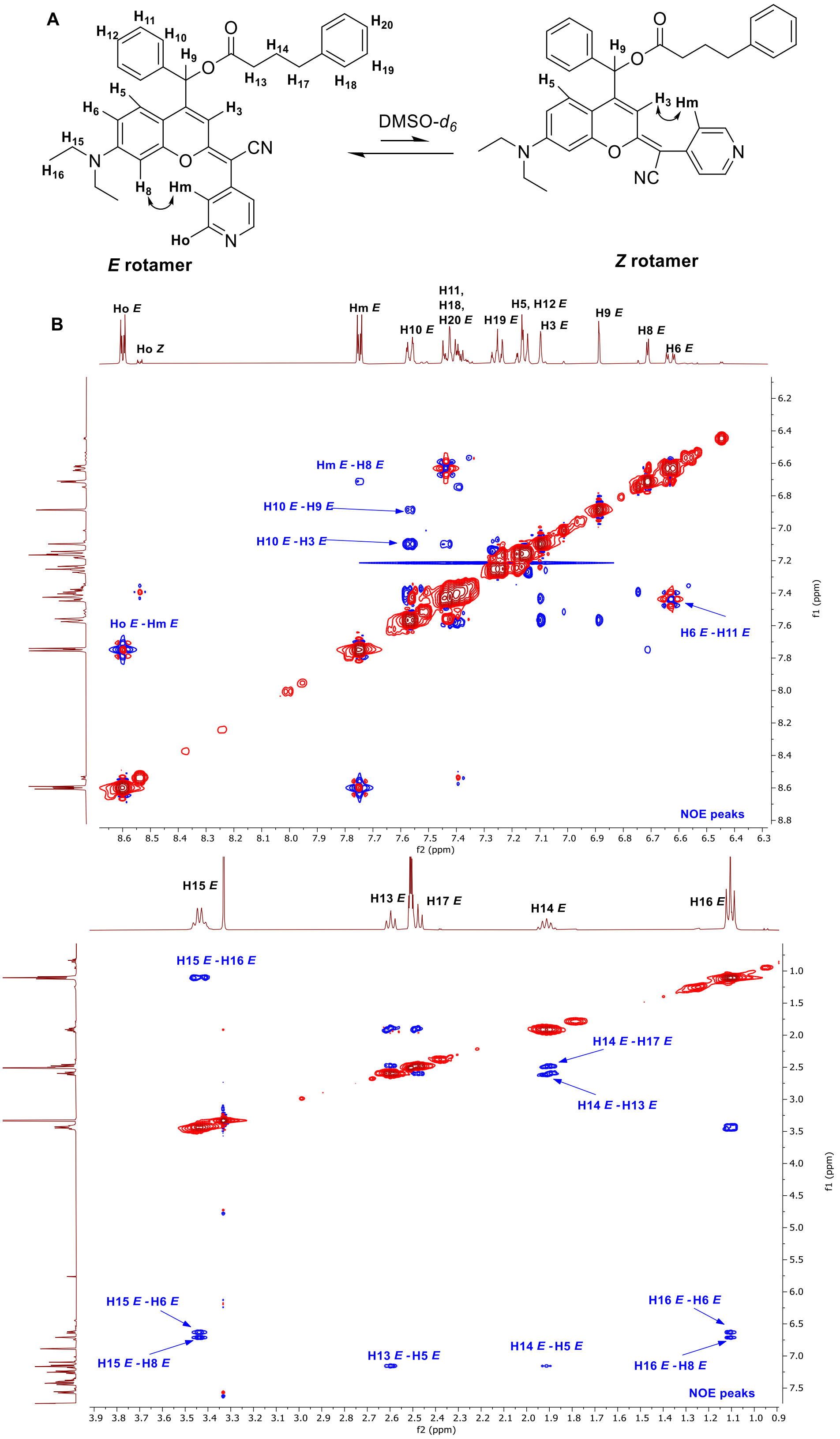

Figure S4. A) Structure of *E* and *Z* rotamers of coumarin **16** with some diagnostic NOE cross-peaks indicated. B) Expansions of the NOESY spectrum (DMSO-d_6, 298 K) of coumarin **16** showing some characteristic NOE cross-peaks.

3.- 2D NMR characterization of COUPY photocages (5Ph, 6Ph, 7-9).

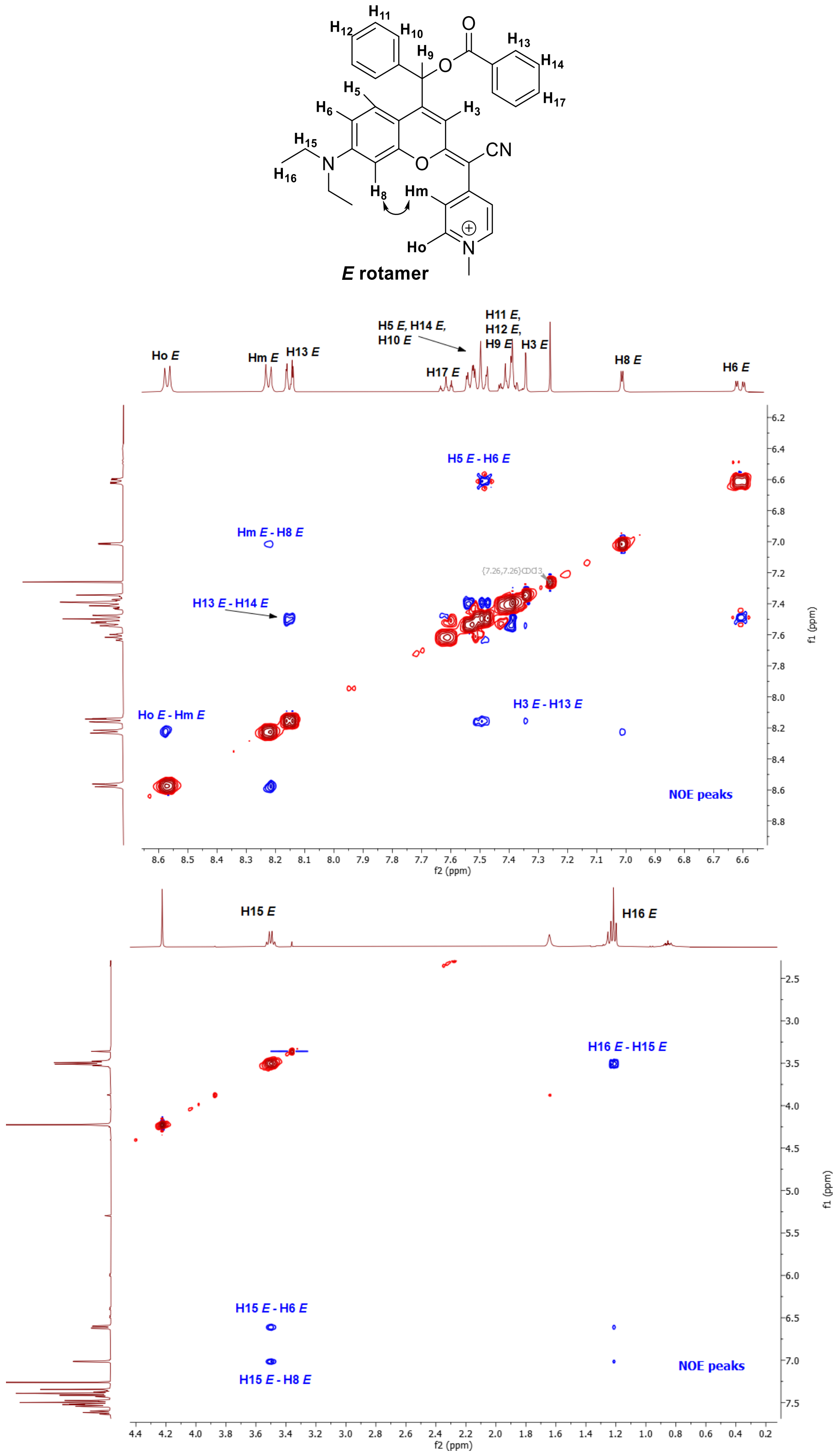

Figure S5. A) Structure of *E* rotamer of coumarin **5Ph** with some diagnostic NOE cross-peaks indicated. B) Expansions of the NOESY spectrum ($CDCl_3$, 298 K) showing some characteristic NOE cross-peaks.

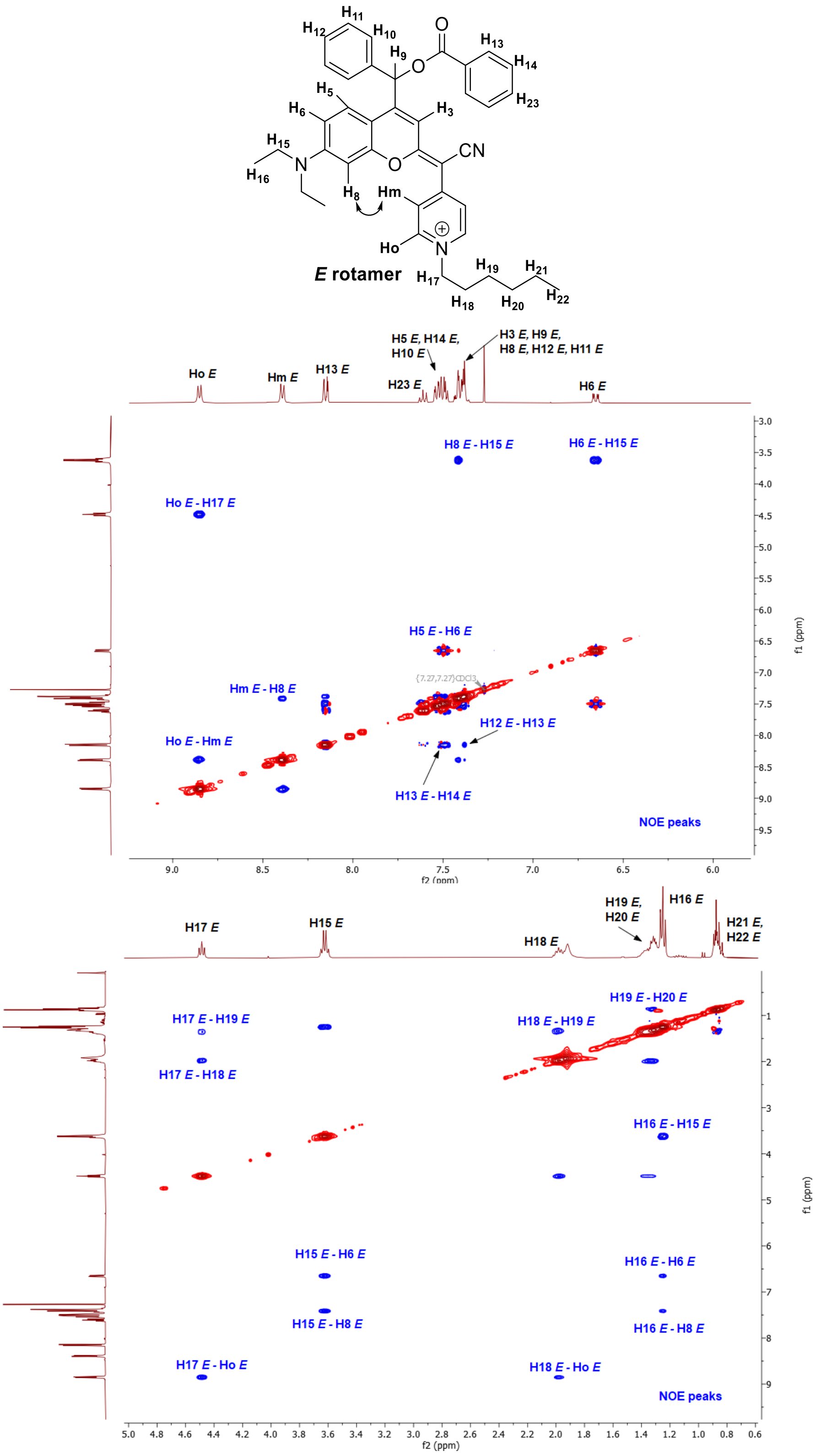

Figure S6. A) Structure of *E* rotamer of coumarin **6Ph** with some diagnostic NOE cross-peaks indicated. B) Expansions of the NOESY spectrum ($CDCl_3$, 298 K) showing some characteristic NOE cross-peaks.

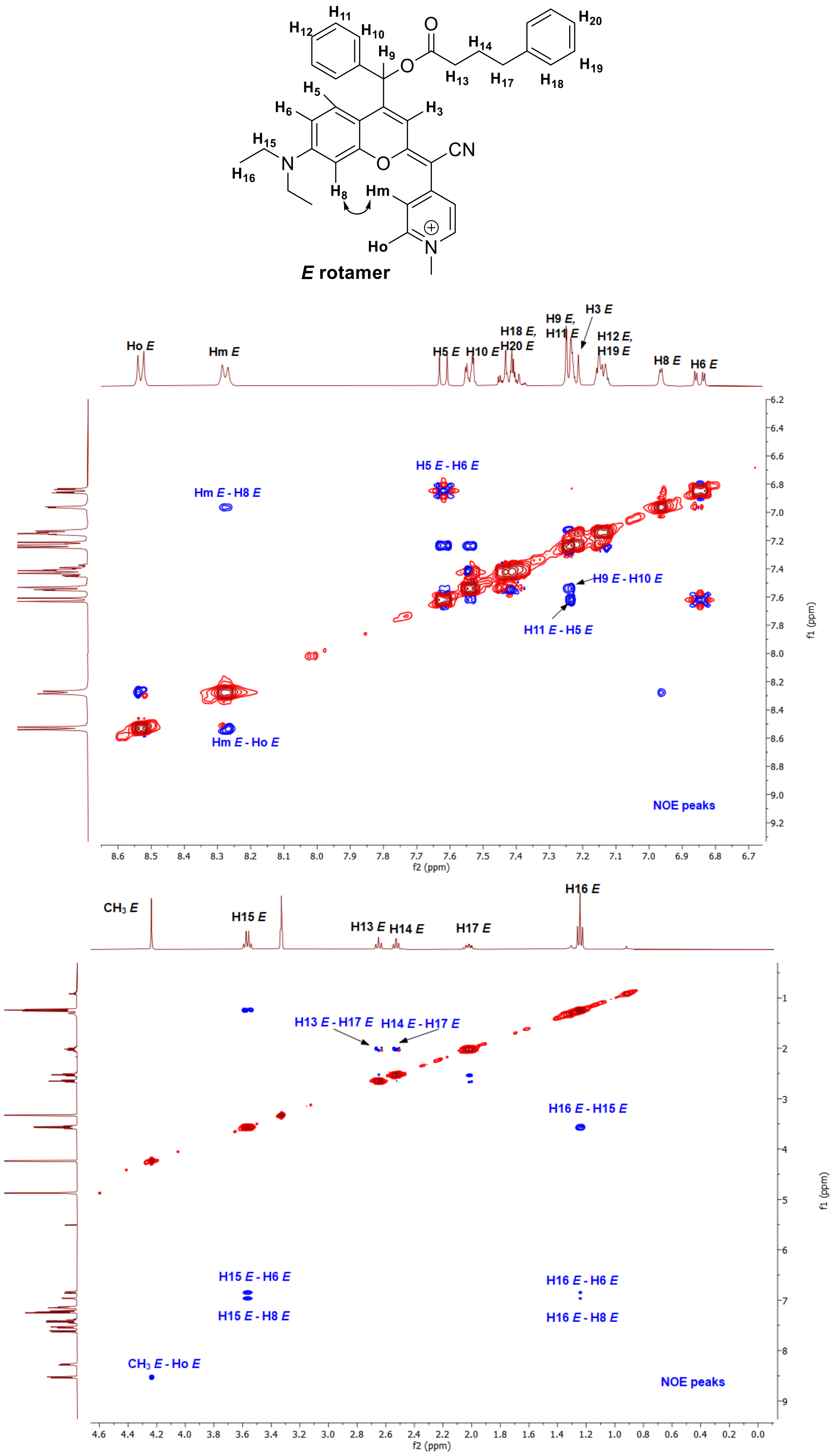

Figure S7. A) Structure of *E* rotamer of coumarin **7** with some diagnostic NOE cross-peaks indicated. B) Expansions of the NOESY spectrum (CD_3OD, 298 K) showing some characteristic NOE cross-peaks.

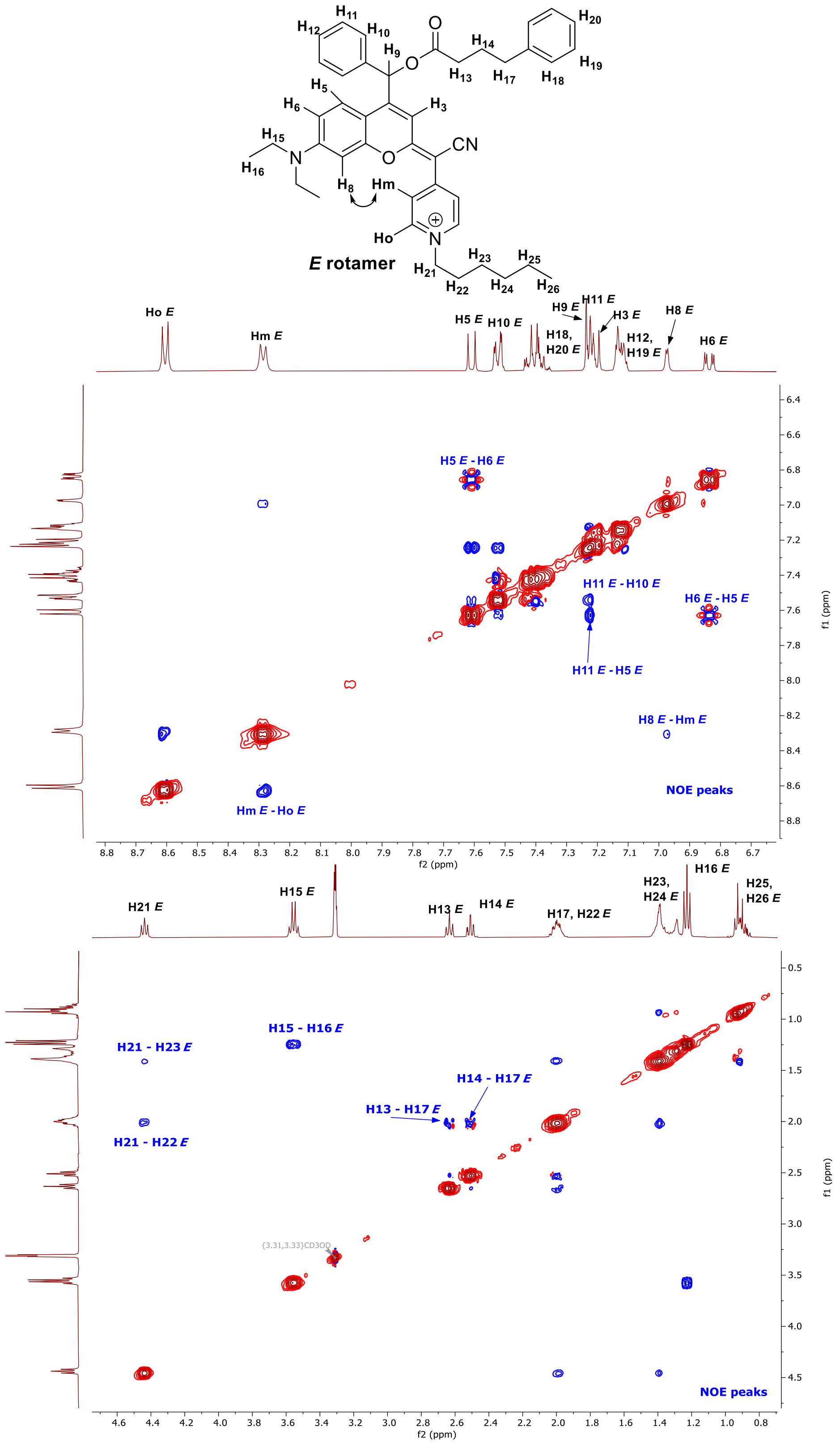

Figure S8. A) Structure of *E* rotamer of coumarin **8** with some diagnostic NOE cross-peaks indicated. B) Expansions of the NOESY spectrum (CD_3OD, 298 K) showing some characteristic NOE cross-peaks.

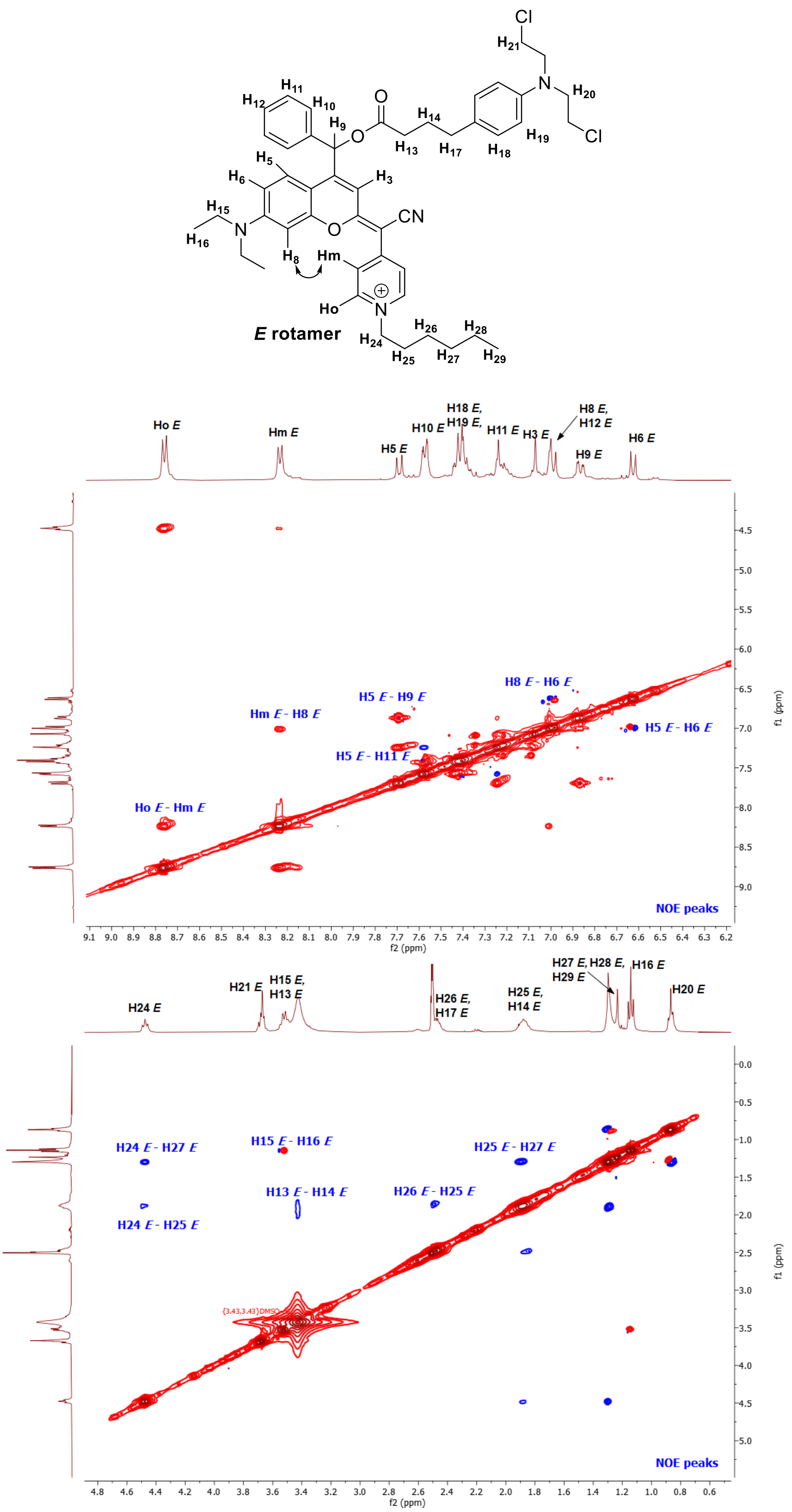

Figure S9. A) Structure of *E* rotamer of coumarin **9** with some diagnostic NOE cross-peaks indicated. B) Expansions of the NOESY spectrum (DMSO-d_6, 298 K) showing some characteristic NOE cross-peaks.

4-. Irradiation experiments

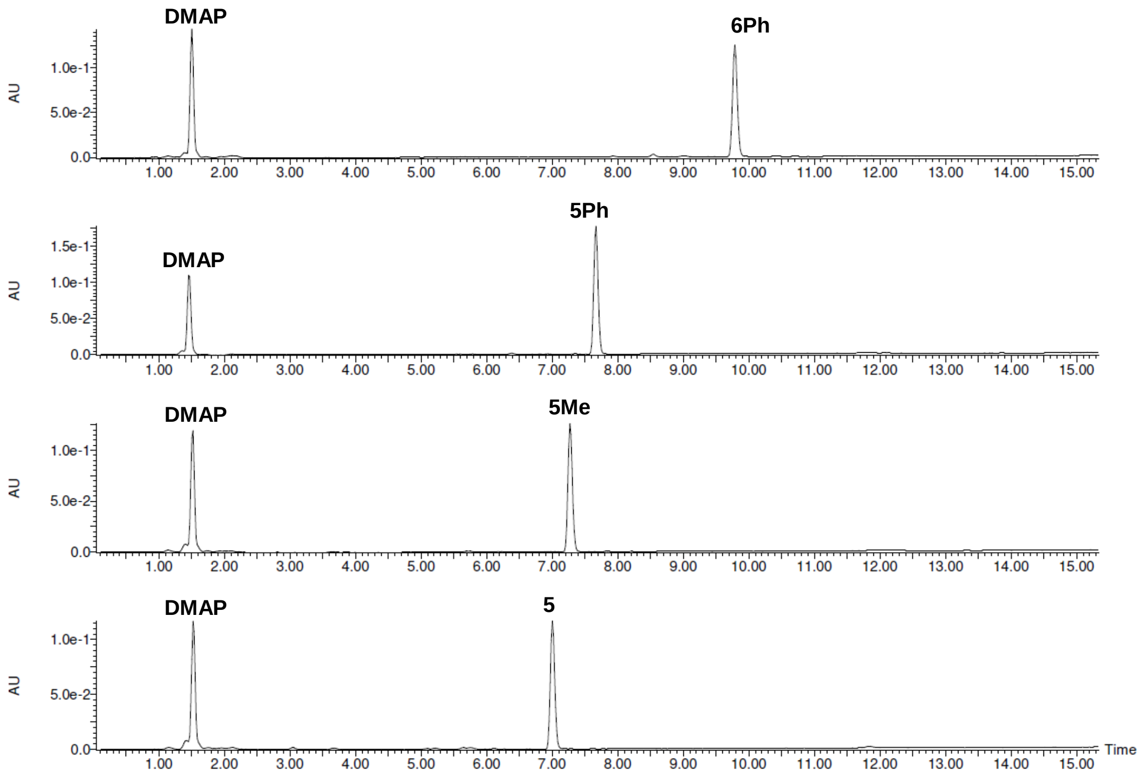

Figure S10. Reversed-phase HPLC-ESI MS traces at 260 nm of COUPY-caged model compounds **5Ph**, **5Me**, **5** and **6Ph** after standing for 2 h in the dark at 37 ºC in a 8:2 (v/v) mixture of PBS buffer and ACN in the presence of DMAP (internal standard) using column 1.

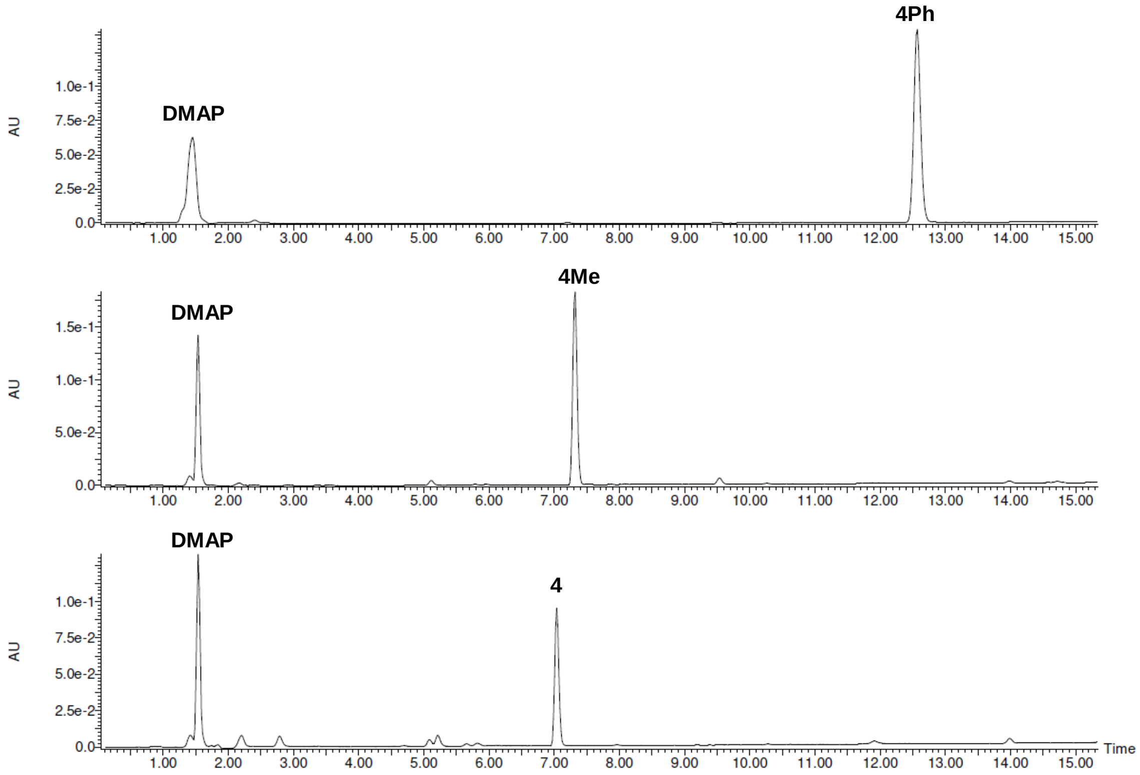

Figure S11. Reversed-phase HPLC-ESI MS traces at 260 nm of dicyanocoumarin-caged **4Ph**, **4Me** and **4** after standing for 2 h in the dark at 37 ºC in a 8:2 (v/v) mixture of PBS buffer and ACN in the presence of DMAP (internal standard) using column 1.

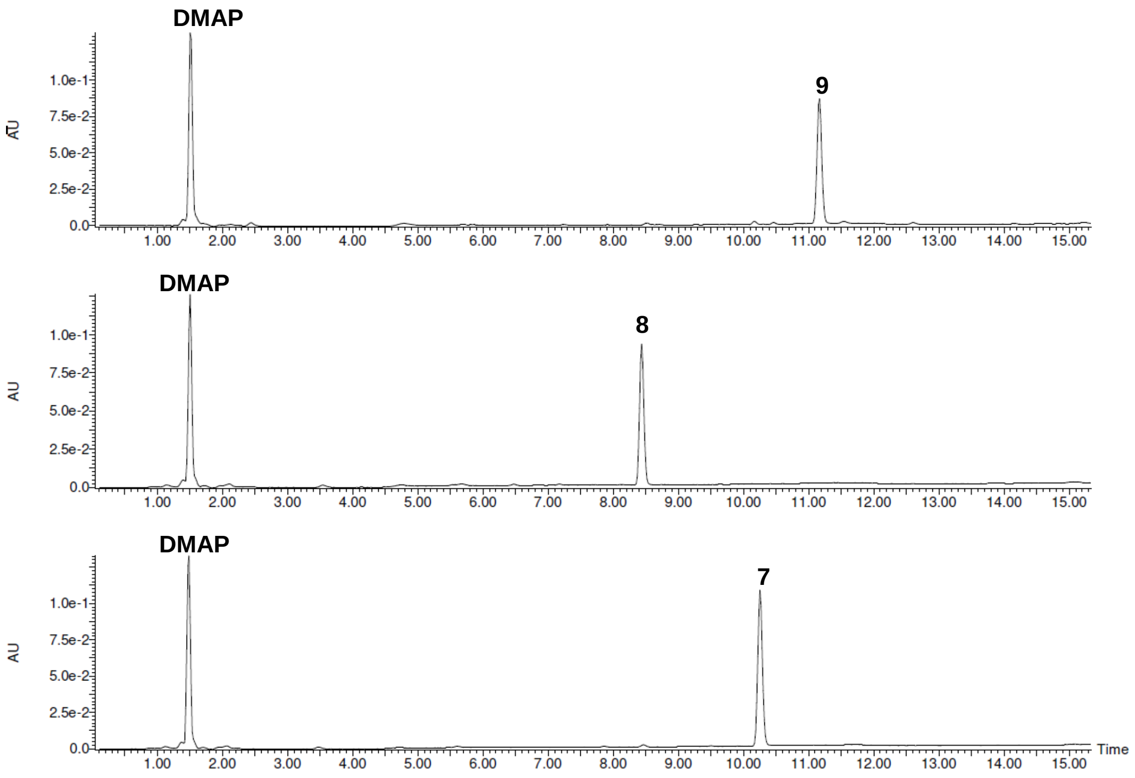

Figure S12. Reversed-phase HPLC-ESI MS traces at 260 nm of COUPY-caged compounds **7-9** after standing for 2 h in the dark at 37 ºC in a 8:2 (v/v) mixture of PBS buffer and ACN in the presence of DMAP (internal standard) using column 1.

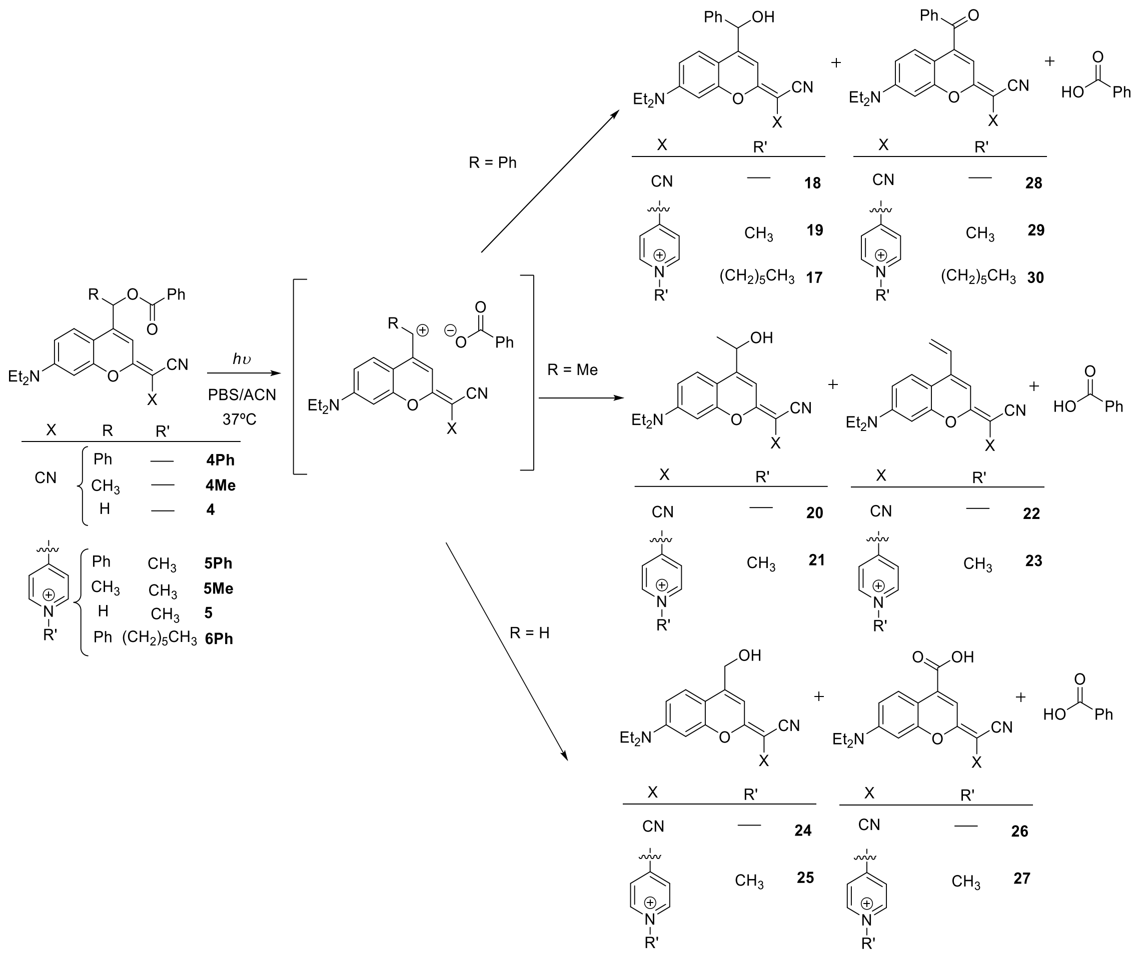

Scheme S1. Mechanistic interpretation of the photolysis of dicyanocoumarin–caged compounds (**4Ph**, **4Me** and **4**) and COUPY-caged model compounds (**5Ph**, **5**, **5Me** and **6Ph**).

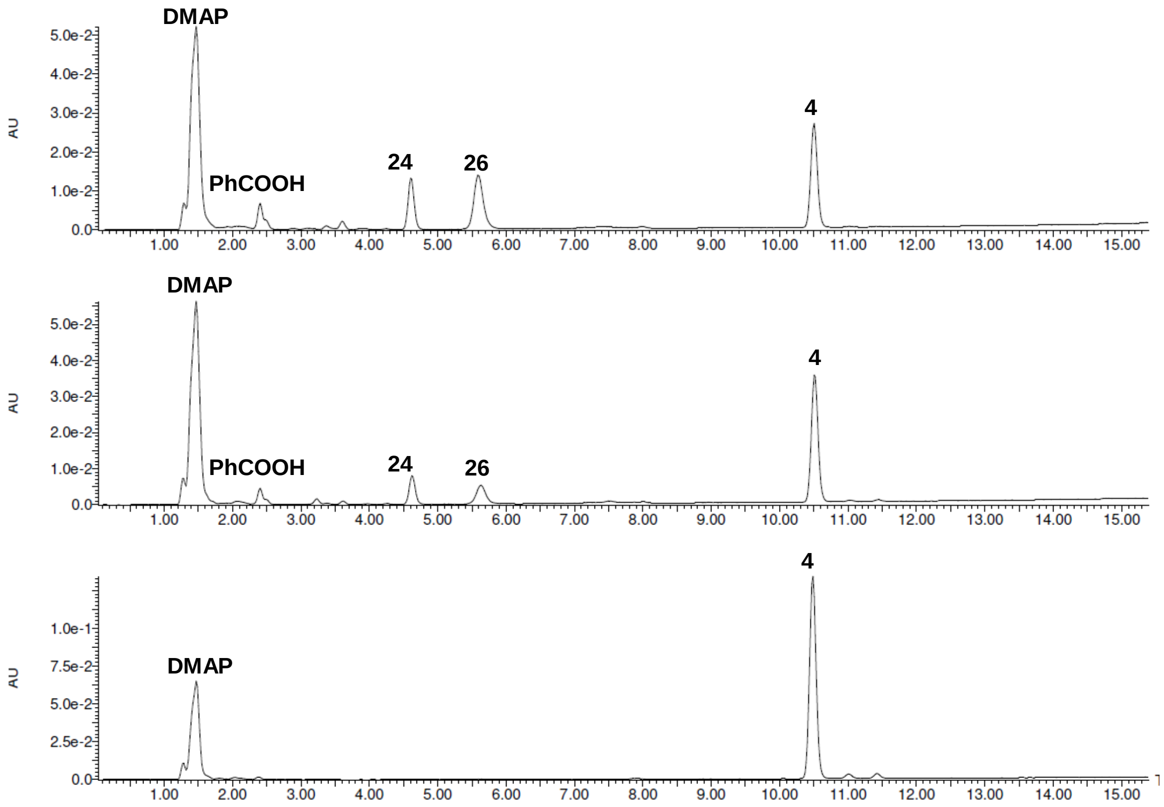

Figure S13 Reversed-phase HPLC-ESI MS traces at 260 nm for the photolysis reaction of **4** in a 8:2 (v/v) mixture of PBS buffer and ACN in the presence of DMAP (internal standard) at t=0 (bottom) and after irradiation with gree LED light (505 nm, 100 mW cm^{-2}) for 30 min (middle) and 150 min (top) at 37 ºC using column 1.

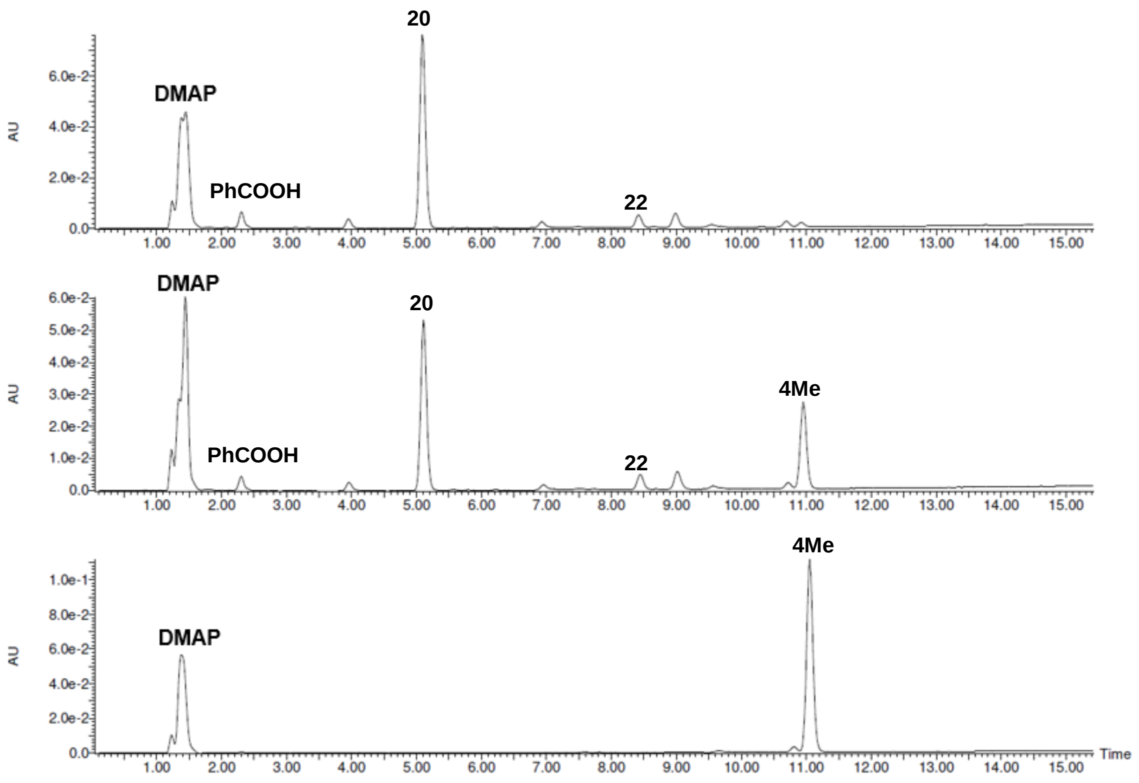

Figure S14. Reversed-phase HPLC-ESI MS traces at 260 nm for the photolysis reaction of **4Me** in a 8:2 (v/v) mixture of PBS buffer and ACN in the presence of DMAP (internal standard) at t=0 (bottom) and after irradiation with green LED light (505 nm, 100 mW cm^{-2}) for 2 min (middle) and 45 min (top) at 37 ºC using column 1.

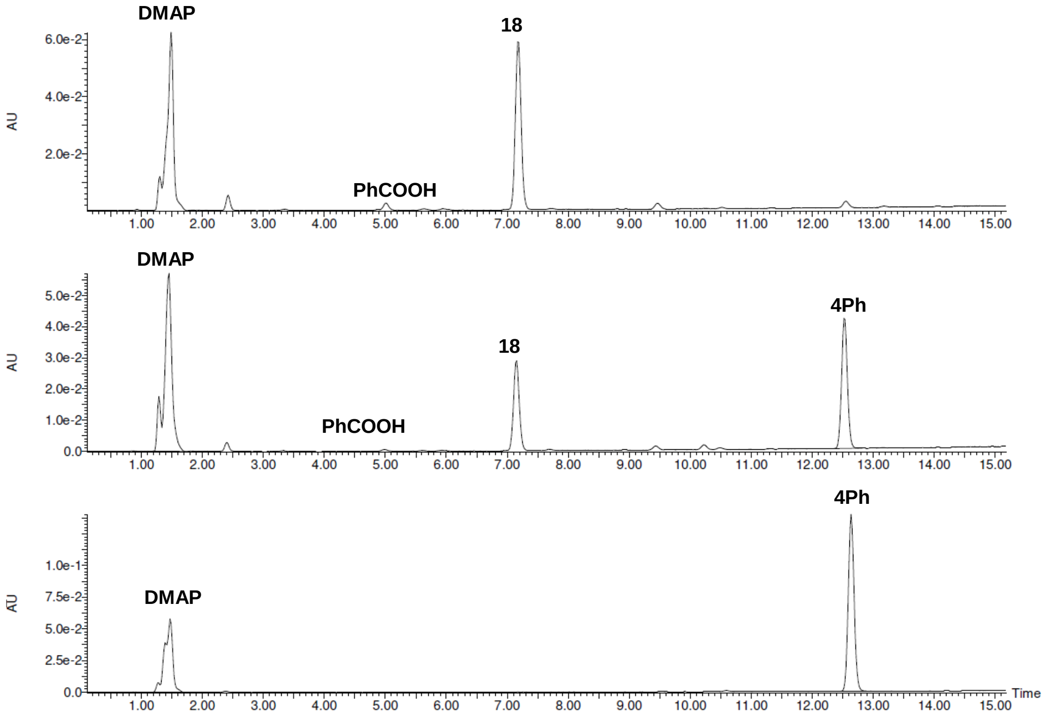

Figure S15. Reversed-phase HPLC-ESI MS traces at 260 nm for the photolysis reaction of **4Ph** in a 8:2 (v/v) mixture of PBS buffer and ACN in the presence of DMAP (internal standard) at t=0 (bottom) and after irradiation with green LED light (505 nm, 100 mW cm^{-2}) for 2 min (middle) and 20 min (top) at 37 ºC using column 1.

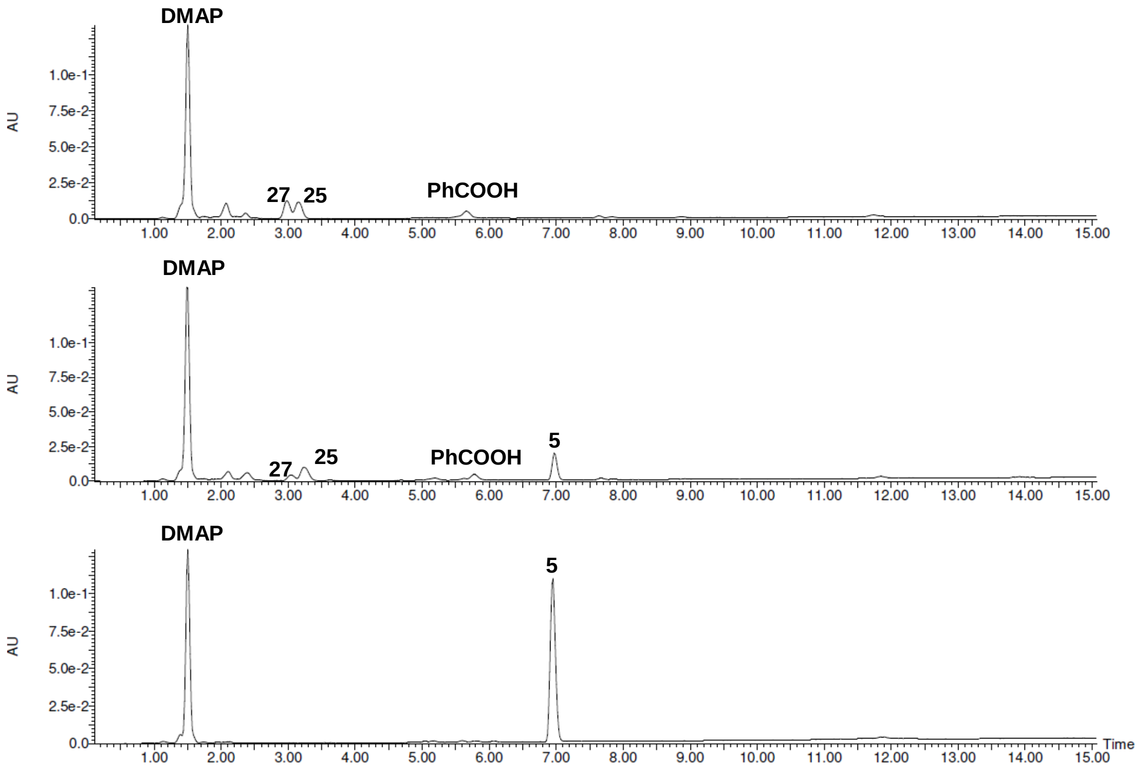

Figure S16. Reversed-phase HPLC-ESI MS traces at 260 nm for the photolysis reaction of **5** in a 8:2 (v/v) mixture of PBS buffer and ACN in the presence of DMAP (internal standard) at t=0 (bottom) and after irradiation with visible LED light (470-750 nm range, centered at 530 nm; 150 mW cm^{-2}) for 20 min (middle) and 45 min (top) at 37 ºC using column 1.

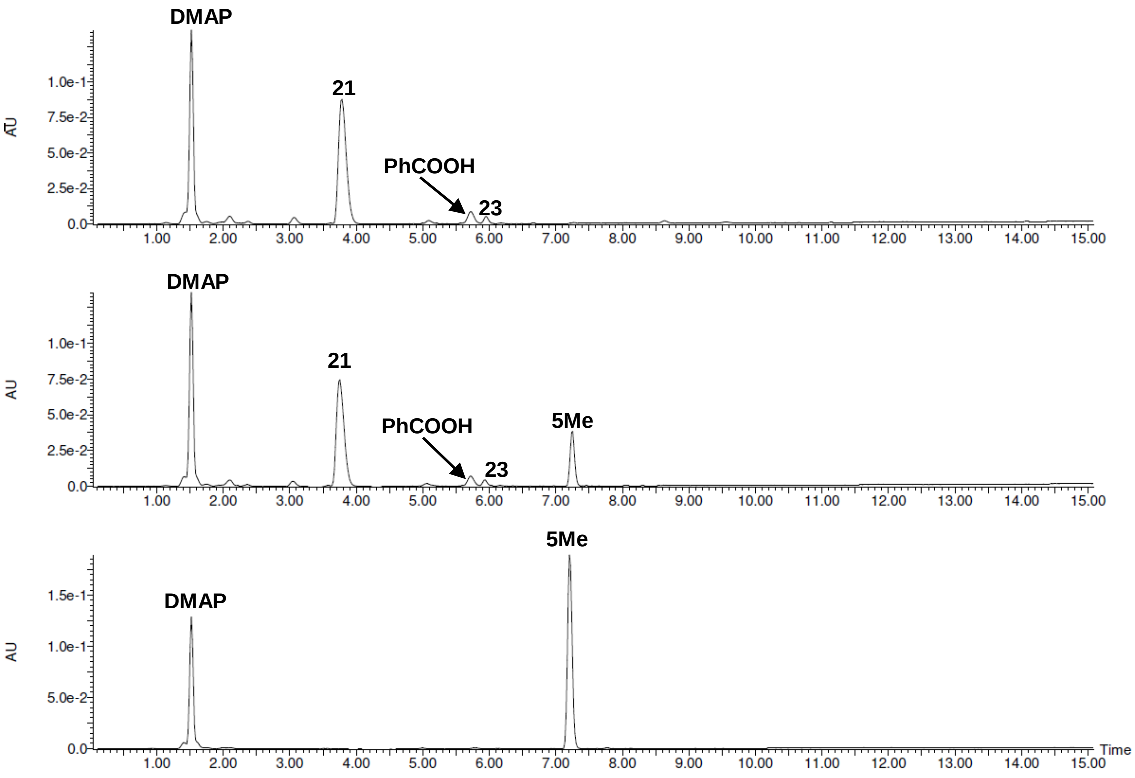

Figure S17. Reversed-phase HPLC-ESI MS traces at 260 nm for the photolysis reaction of **5Me** in a 8:2 (v/v) mixture of PBS buffer and ACN in the presence of DMAP (internal standard) at t=0 (bottom) and after irradiation with visible LED light (470-750 nm range, centered at 530 nm; 150 mW cm^{-2}) for 5 min (middle) and 16 min (top) at 37 ºC using column 1.

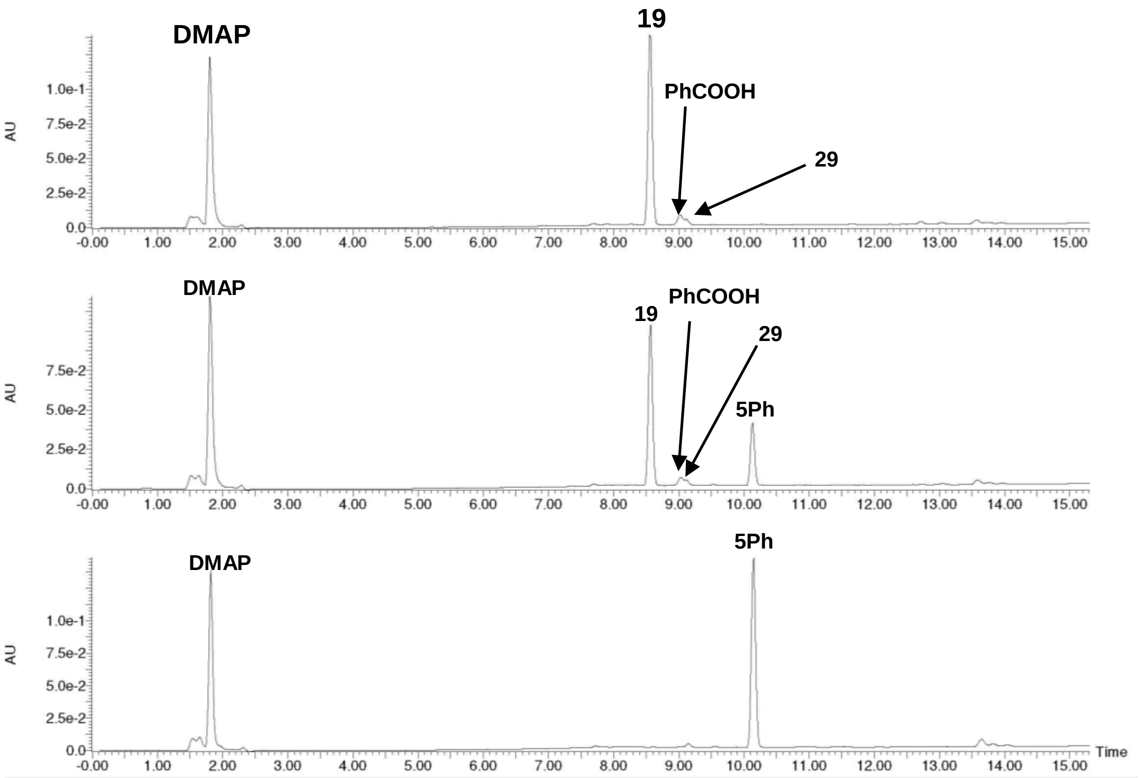

Figure S18. Reversed-phase HPLC-ESI MS traces at 260 nm for the photolysis reaction of **5Ph** in a 8:2 (v/v) mixture of PBS buffer and ACN in the presence of DMAP (internal standard) at t=0 (bottom) and after irradiation with visible LED light (470-750 nm range, centered at 530 nm; 150 mW cm^{-2}) for 1 min (middle) and 7 min (top) at 37 ºC using column 1.

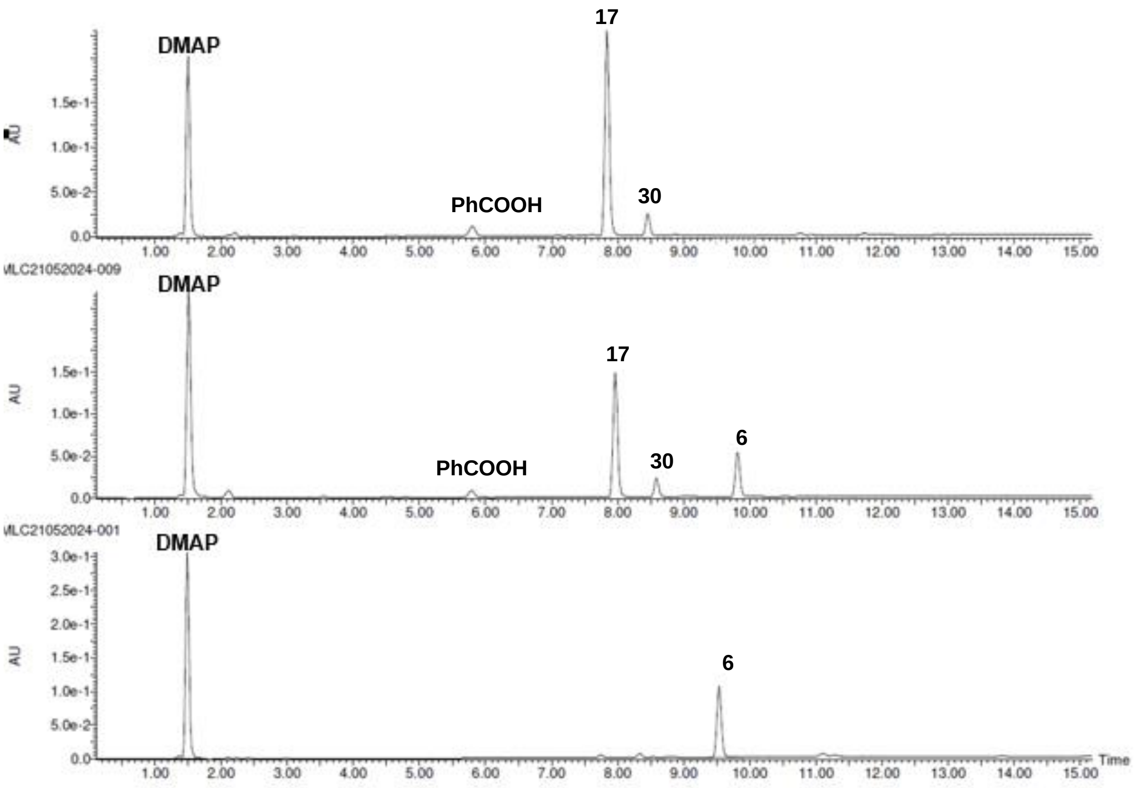

Figure S19. Reversed-phase HPLC-ESI MS traces at 260 nm for the photolysis reaction of **6Ph** in a 8:2 (v/v) mixture of PBS buffer and ACN in the presence of DMAP (internal standard) at t=0 (bottom) and after irradiation with visible LED light (470-750 nm range, centered at 530 nm; 150 mW cm^{-2}) for 1 min (middle) and 10 min (top) at 37 ºC using column 1.

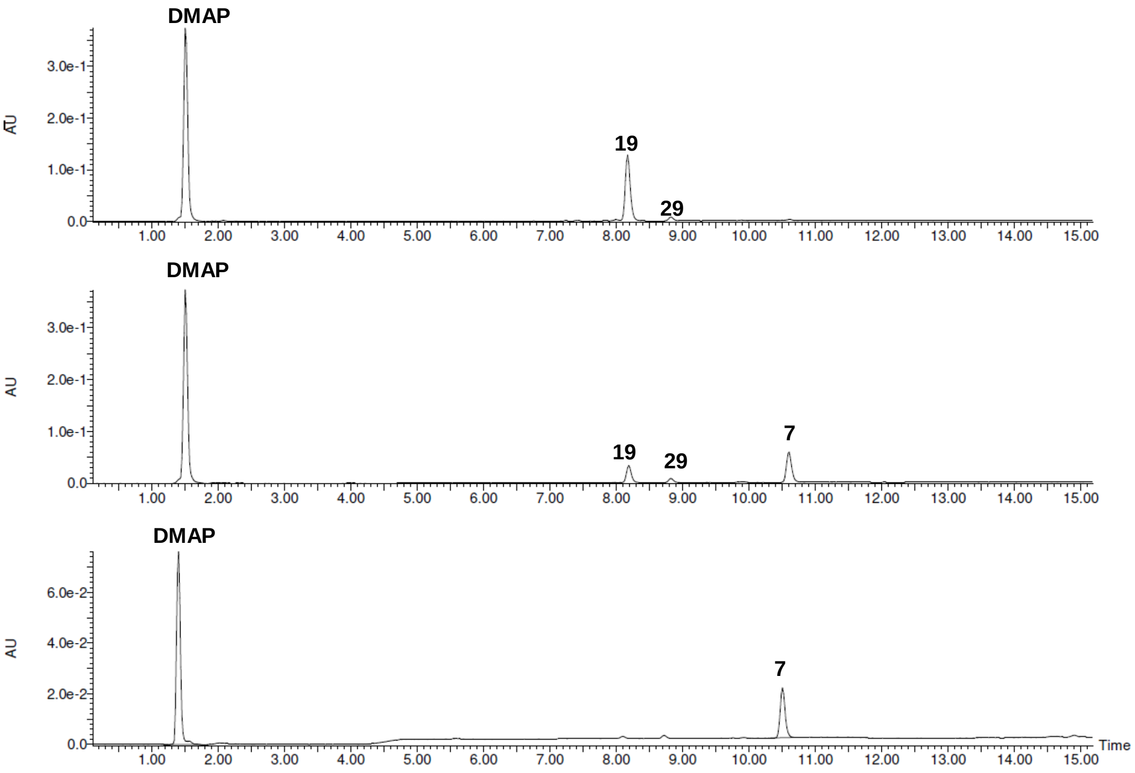

Figure S20. Reversed-phase HPLC-ESI MS traces at 260 nm for the photolysis reaction of **7** in a 8:2 (v/v) mixture of PBS buffer and ACN in the presence of DMAP (internal standard) at t=0 (bottom) and after irradiation with visible LED light (470-750 nm range, centered at 530 nm; 150 mW cm^{-2}) for 5 min (middle) and 15 min (top) at 37 ºC using column 2.

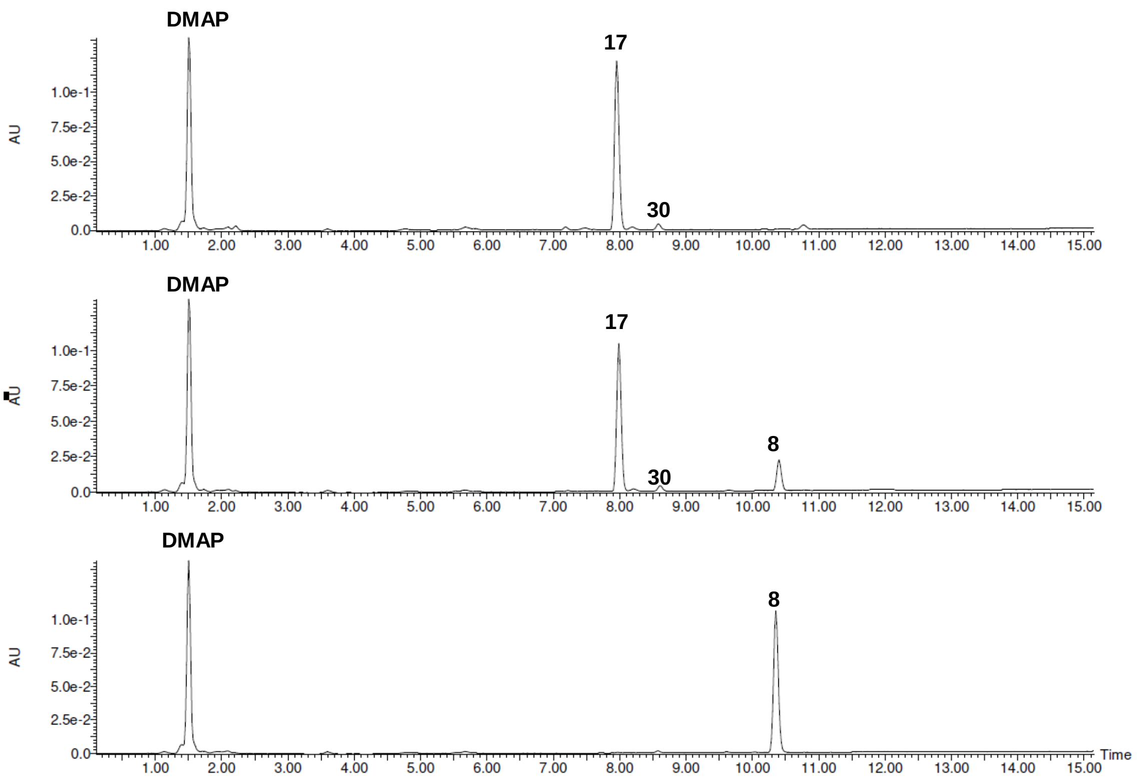

Figure S21. Reversed-phase HPLC-ESI MS traces at 260 nm for the photolysis reaction of **8** in a 8:2 (v/v) mixture of PBS buffer and ACN in the presence of DMAP (internal standard) at t=0 (bottom) and after irradiation with visible LED light (470-750 nm range, centered at 530 nm; 150 mW cm^{-2}) for 1 min (middle) and 15 min (top) at 37 ºC using column 2.

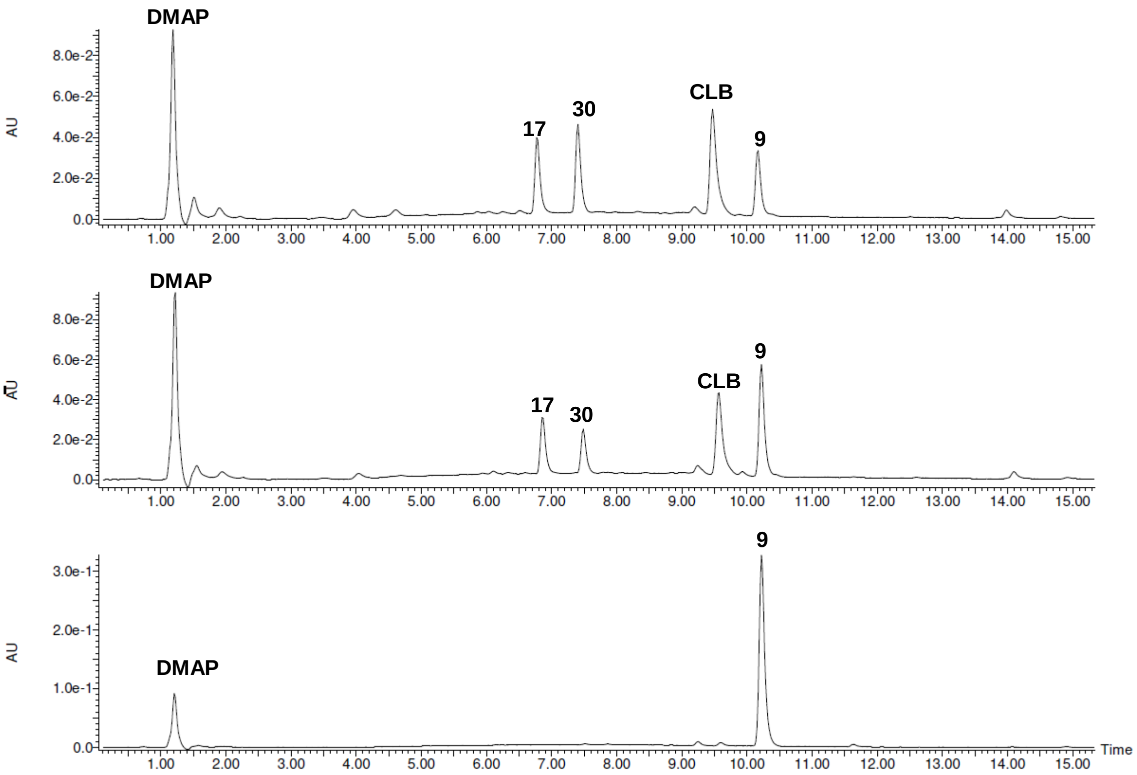

Figure S22. Reversed-phase HPLC-ESI MS traces at 260 nm for the photolysis reaction of **9** in a 8:2 (v/v) mixture of PBS buffer and ACN in the presence of DMAP (internal standard) at t=0 (bottom) and after irradiation with visible LED light (470-750 nm range, centered at 530 nm) for 15 min (middle) and 40 min (top) at 37 ºC using column 2.

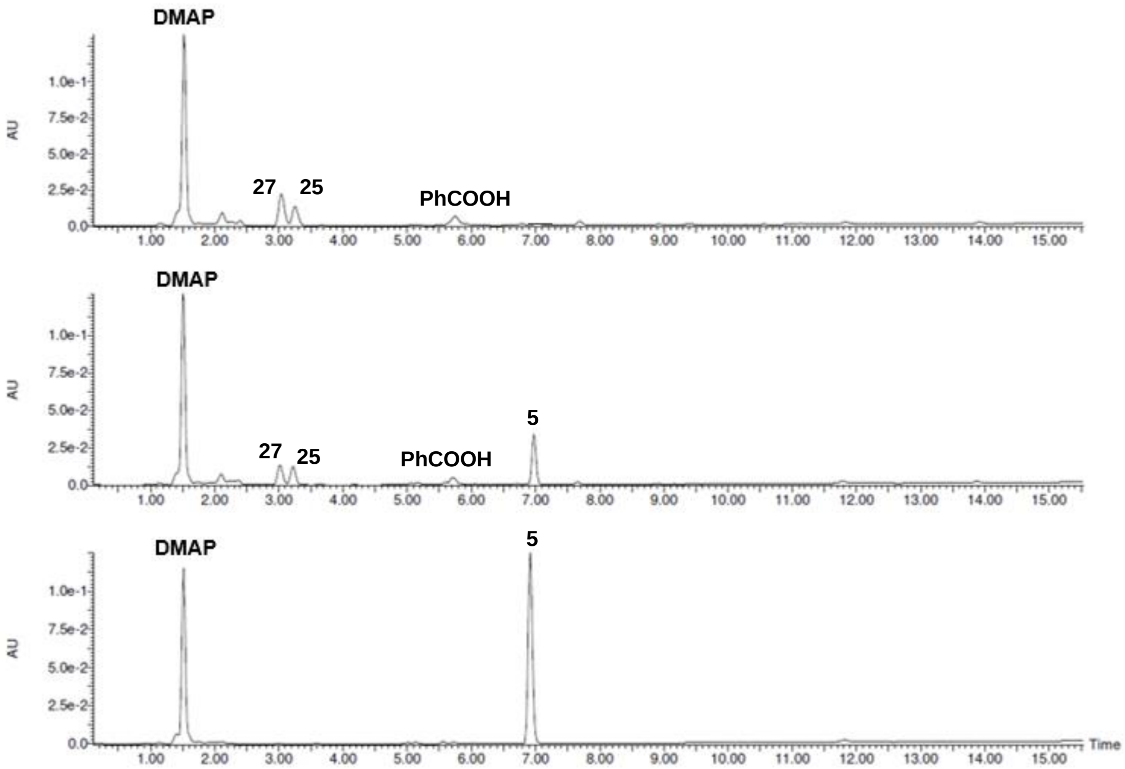

Figure S23. Reversed-phase HPLC-ESI MS traces at 260 nm for the photolysis reaction of **5** in a 8:2 (v/v) mixture of PBS buffer and ACN in the presence of DMAP (internal standard) at t=0 (bottom) and after irradiation with visible LED light (620 nm; 130 mW cm^{-2}) for 60 min (middle) and 210 min (top) at 37 ºC using column 1.

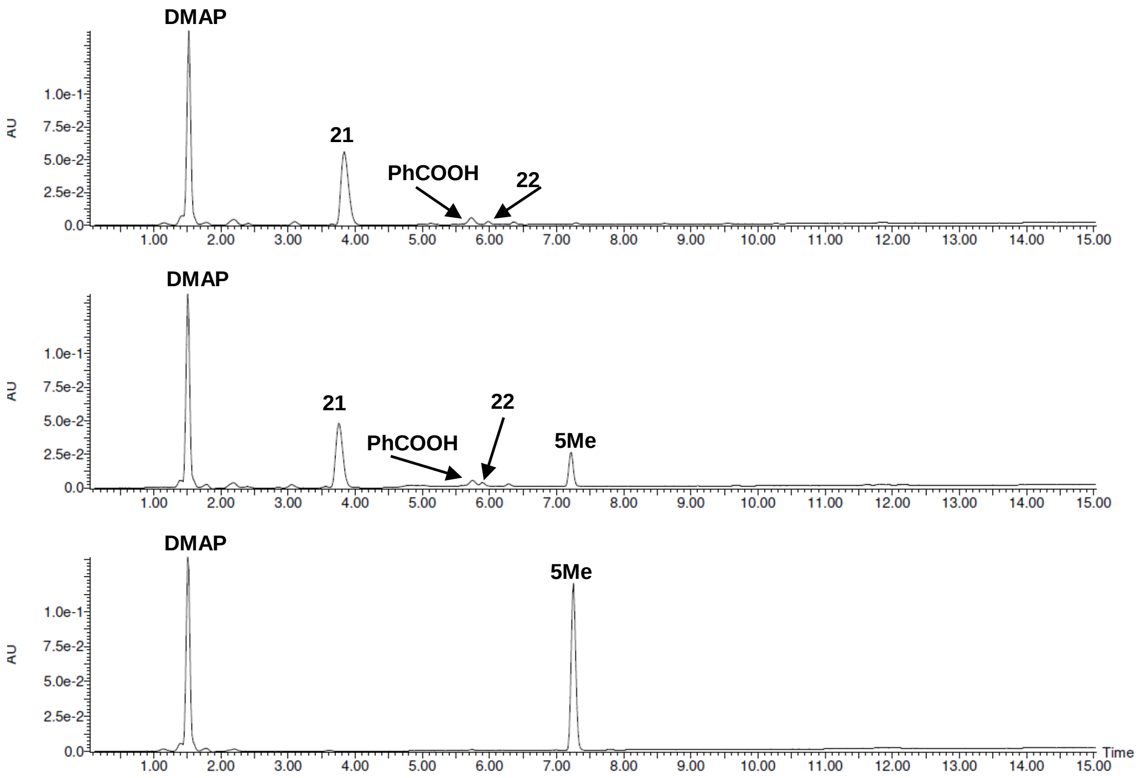

Figure S24. Reversed-phase HPLC-ESI MS traces at 260 nm for the photolysis reaction of **5Me** in a 8:2 (v/v) mixture of PBS buffer and ACN in the presence of DMAP (internal standard) at t=0 (bottom) and after irradiation with visible LED light (620 nm; 130 mW cm^{-2}) for 30 min (middle) and 120 min (top) at 37 ºC using column 1.

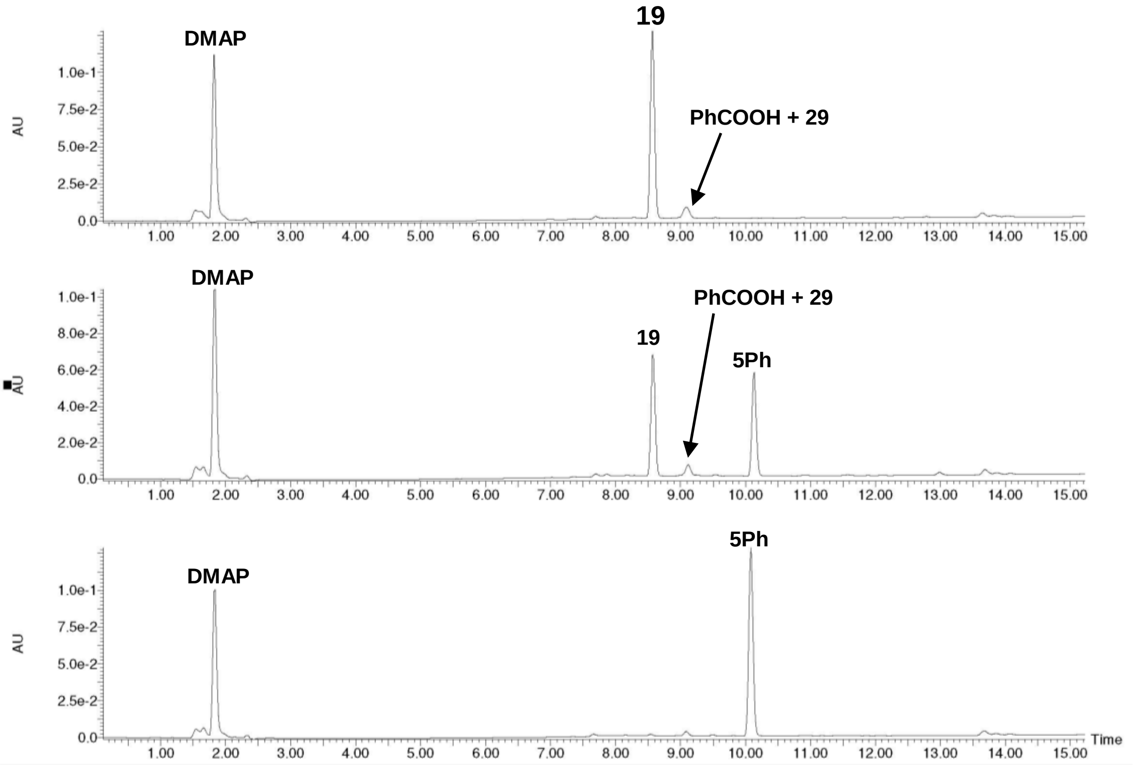

Figure S25. Reversed-phase HPLC-ESI MS traces at 260 nm for the photolysis reaction of **5Ph** in a 8:2 (v/v) mixture of PBS buffer and ACN in the presence of DMAP (internal standard) at t=0 (bottom) and after irradiation with visible LED light (620 nm; 130 mW cm^{-2}) for 20 min (middle) and 45 min (top) at 37 ºC using column 1.

5.- Determination of uncaging quantum yield (Φ_{Phot}) using DAE actinometry protocol

To calculate the uncaging quantum yield of dicyanocoumarin (**4**, **4Me** and **4Ph**) and COUPY (**5**, **5Me** and **5Ph**) photocages, first the photon flux of the light sources was determined by actinometry following a previously reported procedure.[39] The actinometrical measurements using [1,2-bis(2,4-dimethyl-5-phenyl-3-thienyl)perfluorocyclopentene] (DAE), which acts as a visible-light actinometer, involves two steps: (i) conversion of DAE OF (open form) to DAE CF (closed form) under UV irradiation (UV lamp, λ_{max} = 365 nm) for 30 min, and (ii) conversion of DAE CF to DAE OF under the desired visible light irradiation wavelength. As shown in Figure S25, DAE OF only exhibits one absorption band centered at 268 nm, while DAE CF shows two additional bands at 370 nm and 564 nm. Moreover, this can also be easily observed by the colour of the solution: DAE CF appears purple in hexanes, whereas DAE OF is colourless. Once the complete formation of DAE CF was confirmed, the sample was irradiated with visible light over a time range of 1 to 150 seconds to induce ring opening and the UV-Vis spectra were recorded (Figure S25).

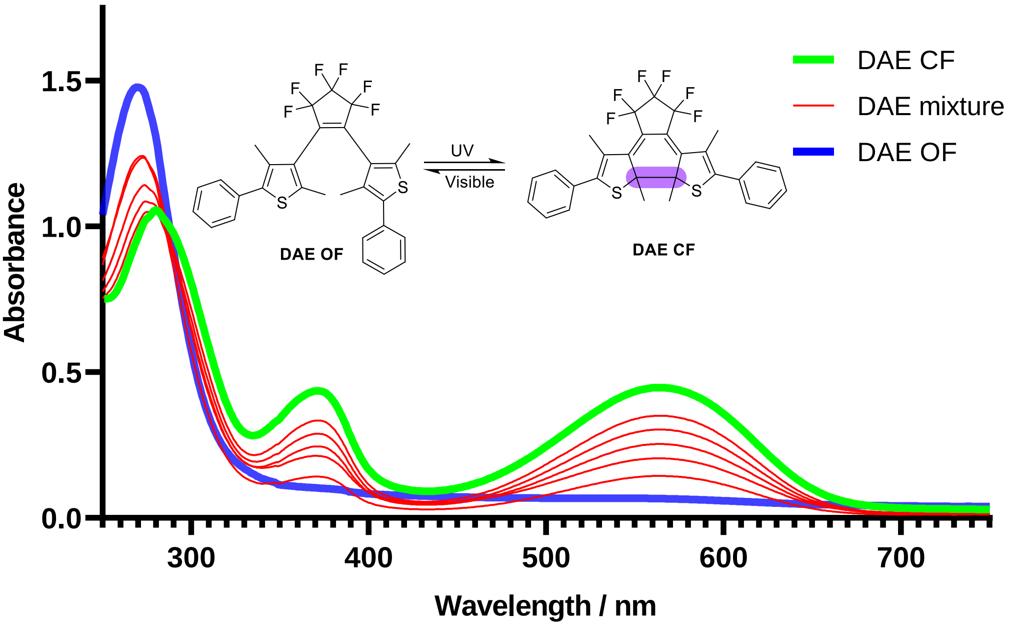

Figure S26. Absorption spectra of DAE OF solution in hexanes (30 µM) before UV irradiation (one band at 268 nm is observed) and of DAE CF after 30 min of UV irradiation (two additional bands at 370 and 564 nm are observed). After irradiation with visible light, the conversion of DAE CF to DAE OF is evidenced by the disappearance of the bands at 370 and 564 nm.

Once the photon flux (I_{abs}) of the light sources (470-750 nm range, centered at 530 nm, 150 mW cm^{-2}; 620 nm, 130 mW cm^{-2}; 505 nm, 100 mW cm^{-2}) were determined (6.19 x 10^{-7} Einstein s^{-1}, 4.96 x 10^{-7} Einstein s^{-1} or 3.98 x 10^{-7}Einstein s^{-1}, respectively),[40,41,42] the uncaging quantum yield (Φ_{Phot}) of COUPY photocages **4-6** was calculated using the following equation:

$$\frac{d[PPG]}{t} = -\phi_{\mathrm{Phot}} \frac{I_{absco}}{V}$$

in which I_{absco} represents the corrected photon flux that only considers the number of photons absorbed by the COUPY photocage and not by the photoreleased coumarin alcohol.

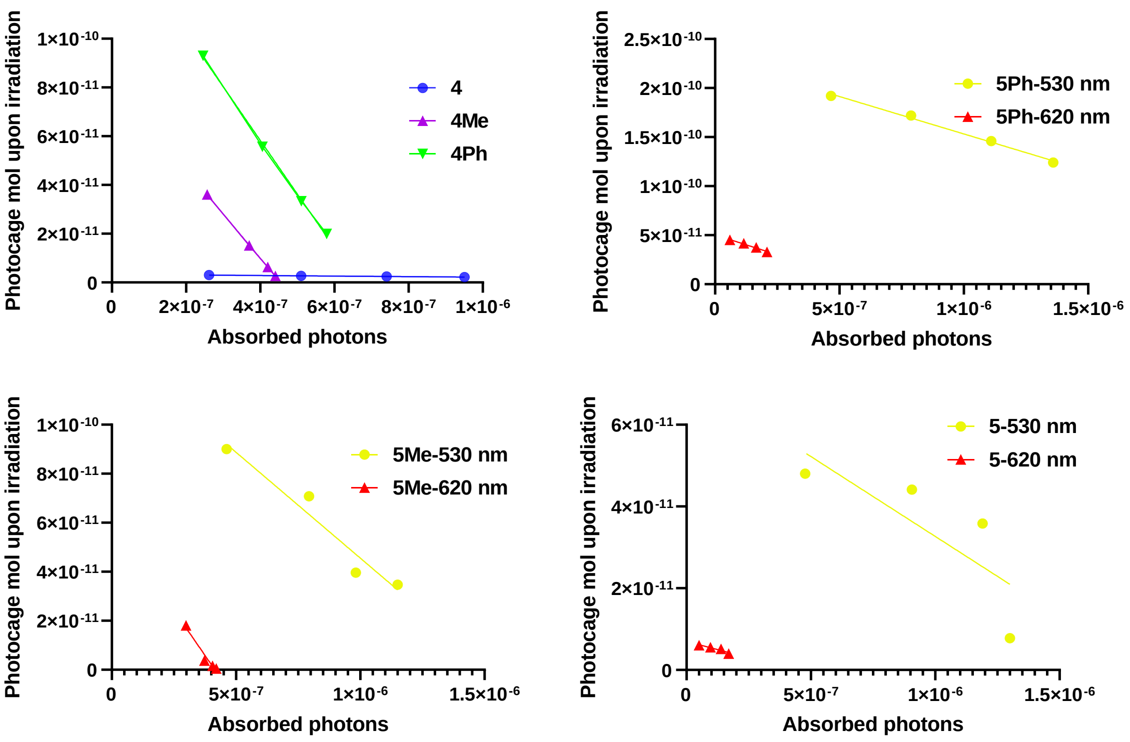

Figure S27. Determination of photochemical quantum yields using different LEDs (green light for **4Ph**, **4Me** and **4**; visible light and red light for **5Ph**, **5Me** and **5**) from the decrease of photocages as absorbed light dose increases. Photolysis quantum yields were calculated as the initial slope of the plot of the amount of coumarin photocage deprotected *vs* the number of photons absorbed. Only the initial points were included in the calculation to avoid inner-filter effects due to the coumarin photoproducts, which absorb in the same spectral range and thus could slow down the process as the reaction progresses.

6.- ^{1}H and ^{13}C NMR spectra and HR ESI-MS of the compounds

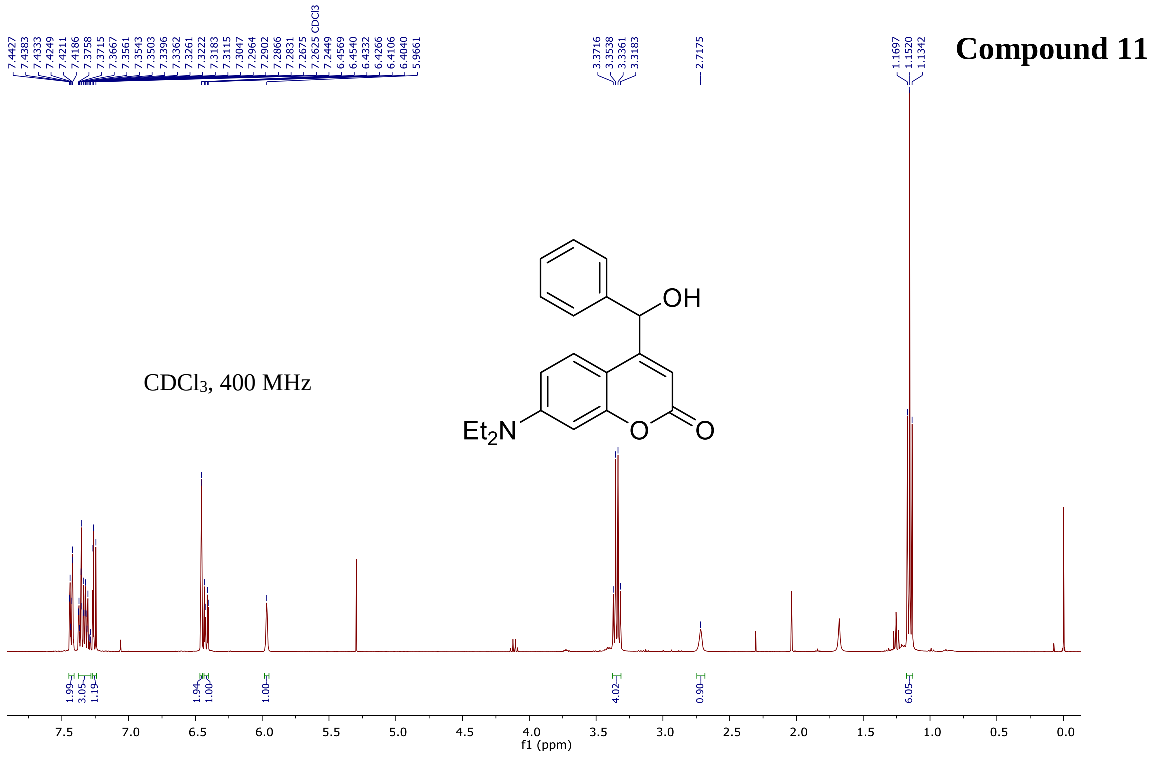

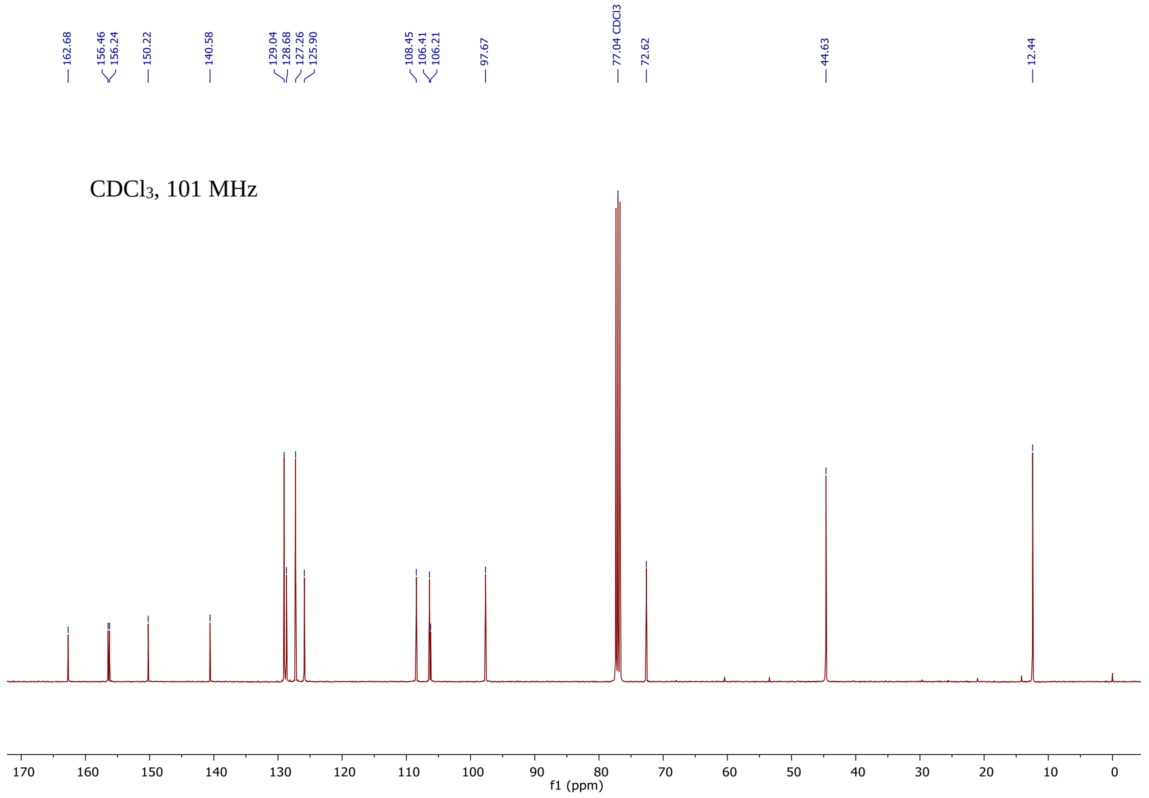

Figure S28. ^{1}H and ^{13}C NMR spectra of compound **11** in $CDCl_3$.

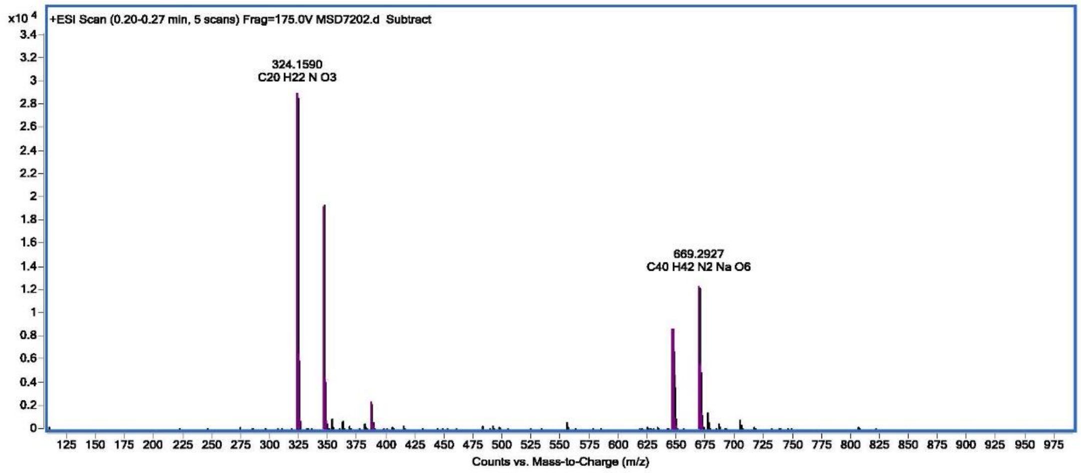

Figure S29. HR ESI-MS spectrum of compound **11**.

Compound 12

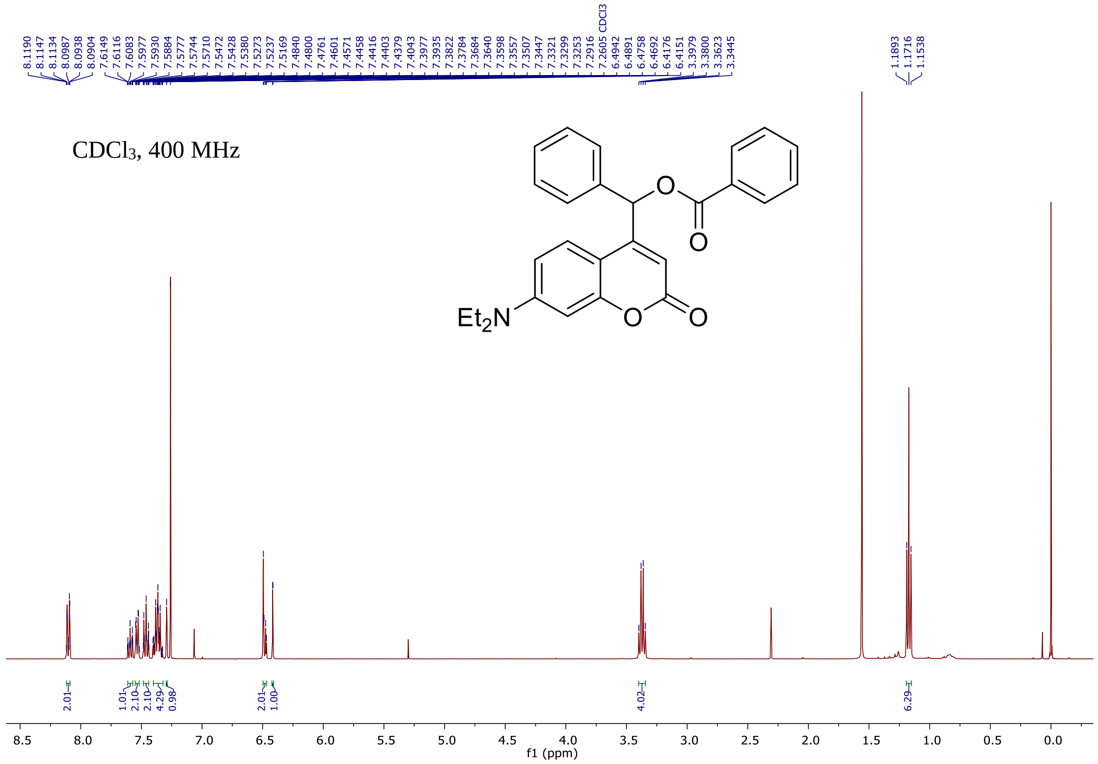

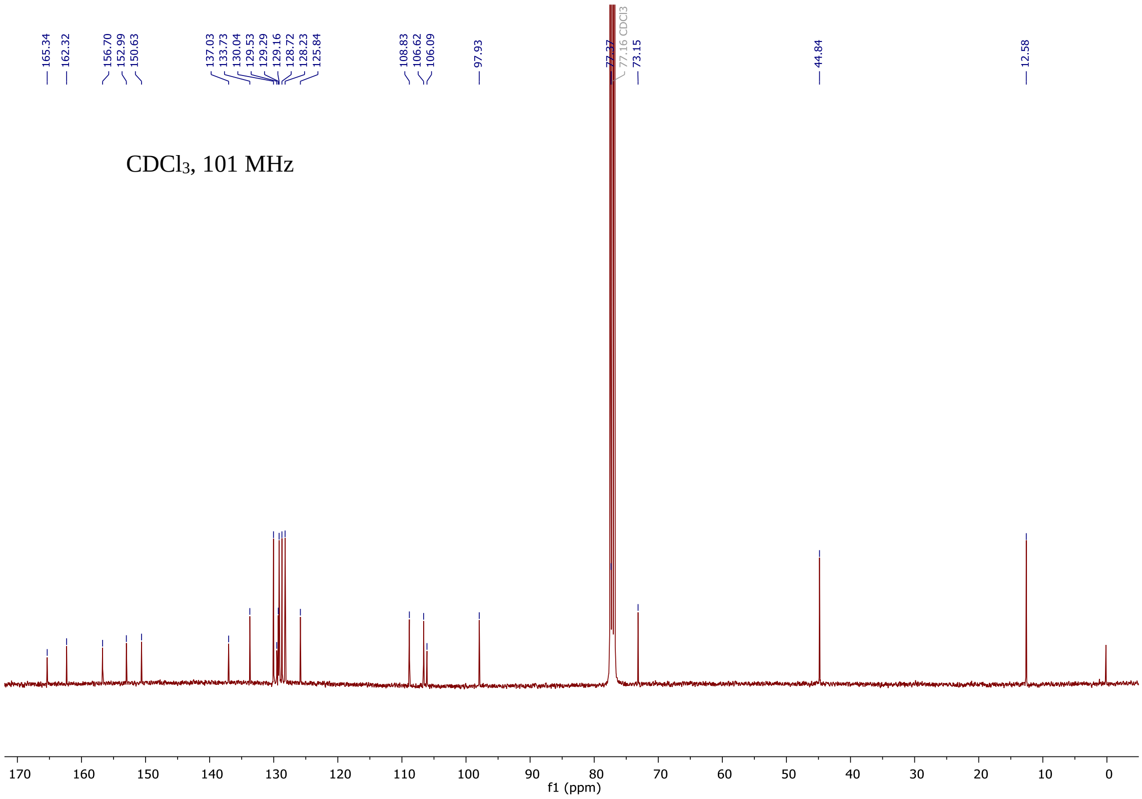

Figure S30. ¹H and ¹³C NMR spectra of compound **12** in $CDCl_3$.

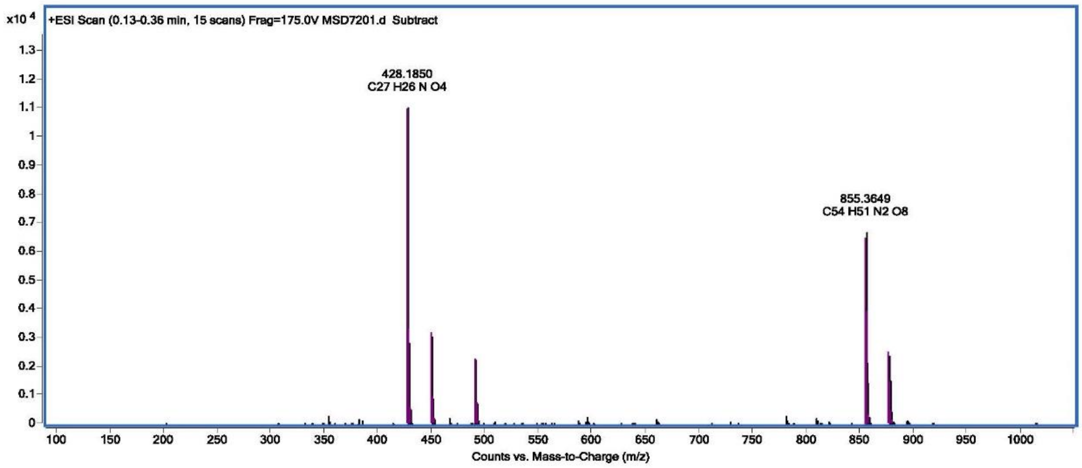

Figure S31. HR ESI-MS spectrum of compound **12**.

Compound 13

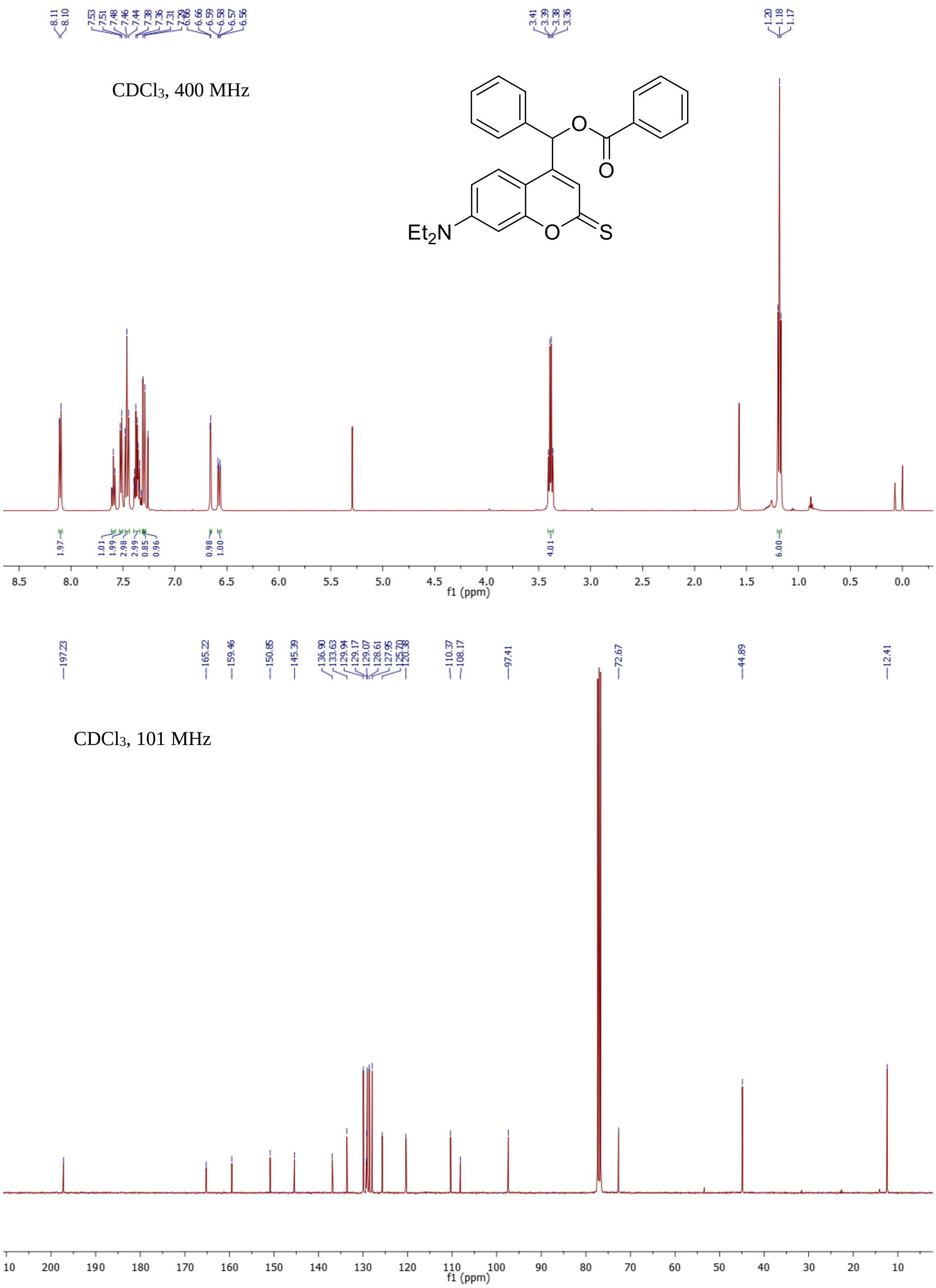

Figure S32. ^{1}H and ^{13}C NMR spectra of compound **13** in $CDCl_3$.

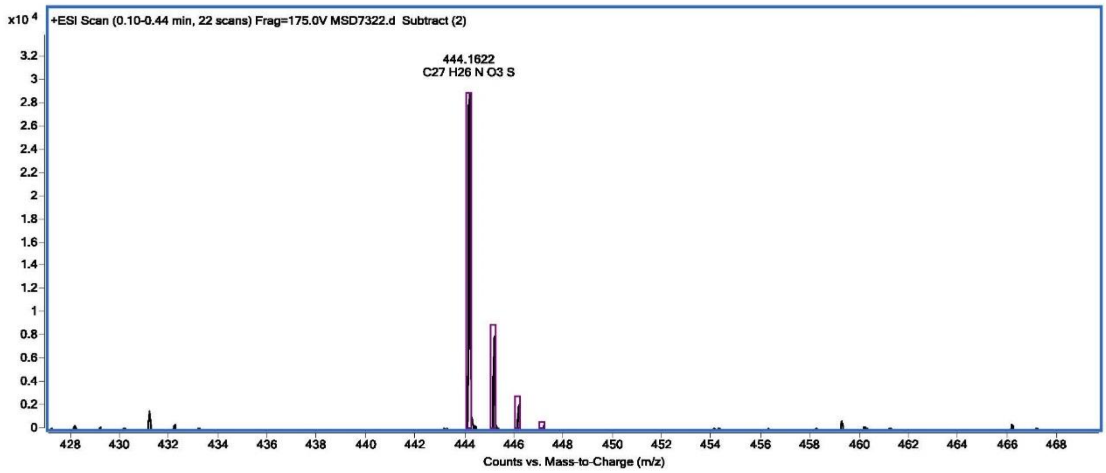

Figure S33. HR ESI-MS spectrum of compound **13**.

Compound 14

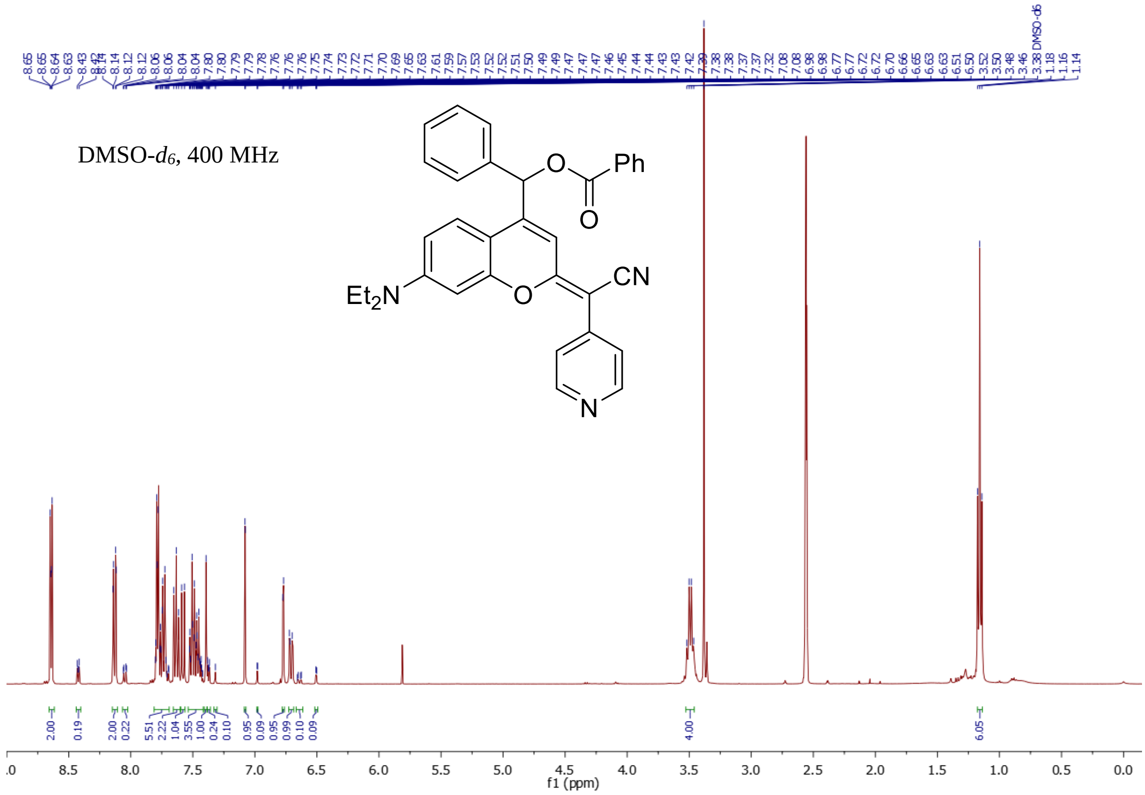

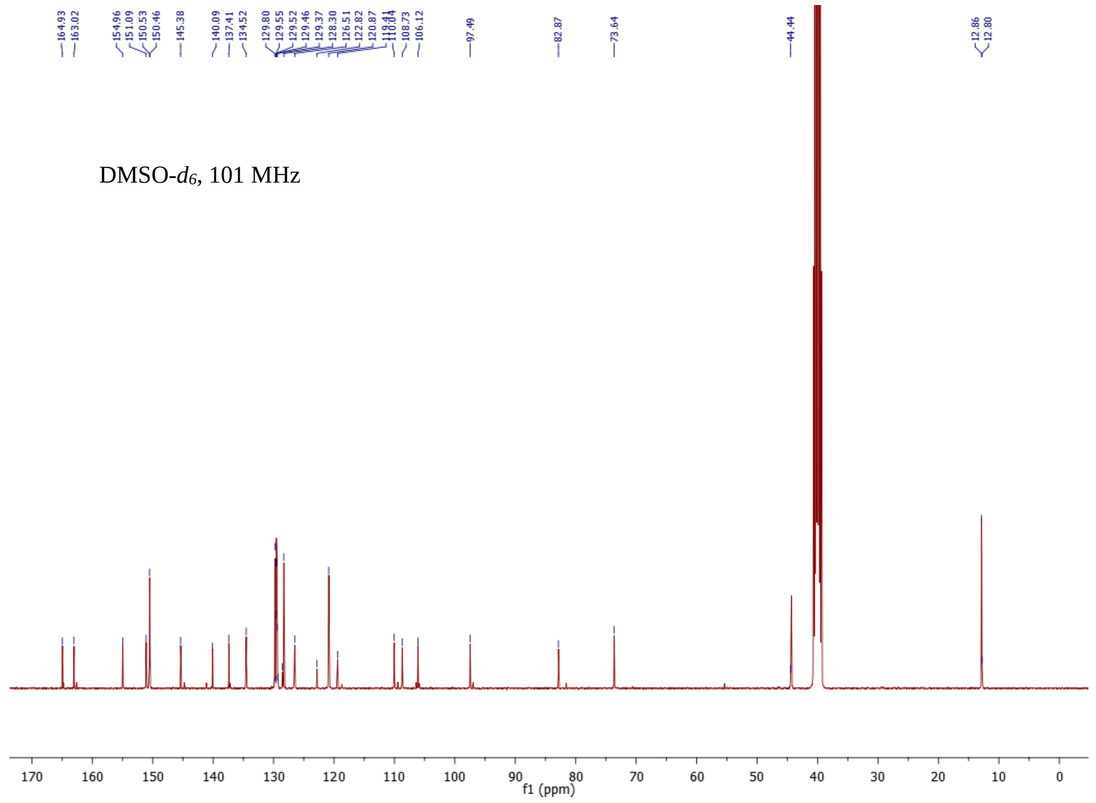

Figure S34. ^1H and ^{13}C NMR spectra of compound **14** in DMSO-d_6.

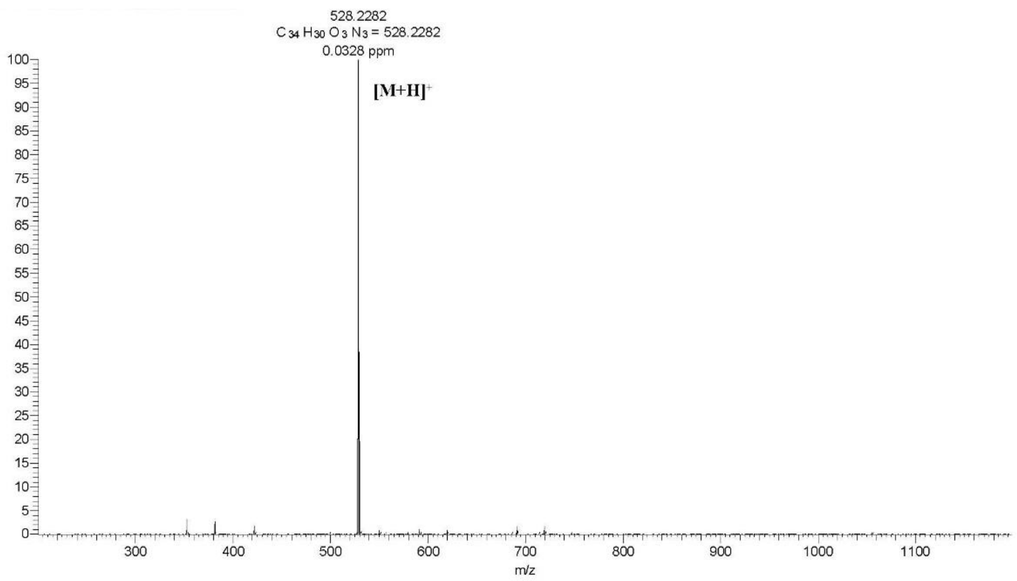

Figure S35. HR ESI-MS spectrum of compound **14**.

Compound 15

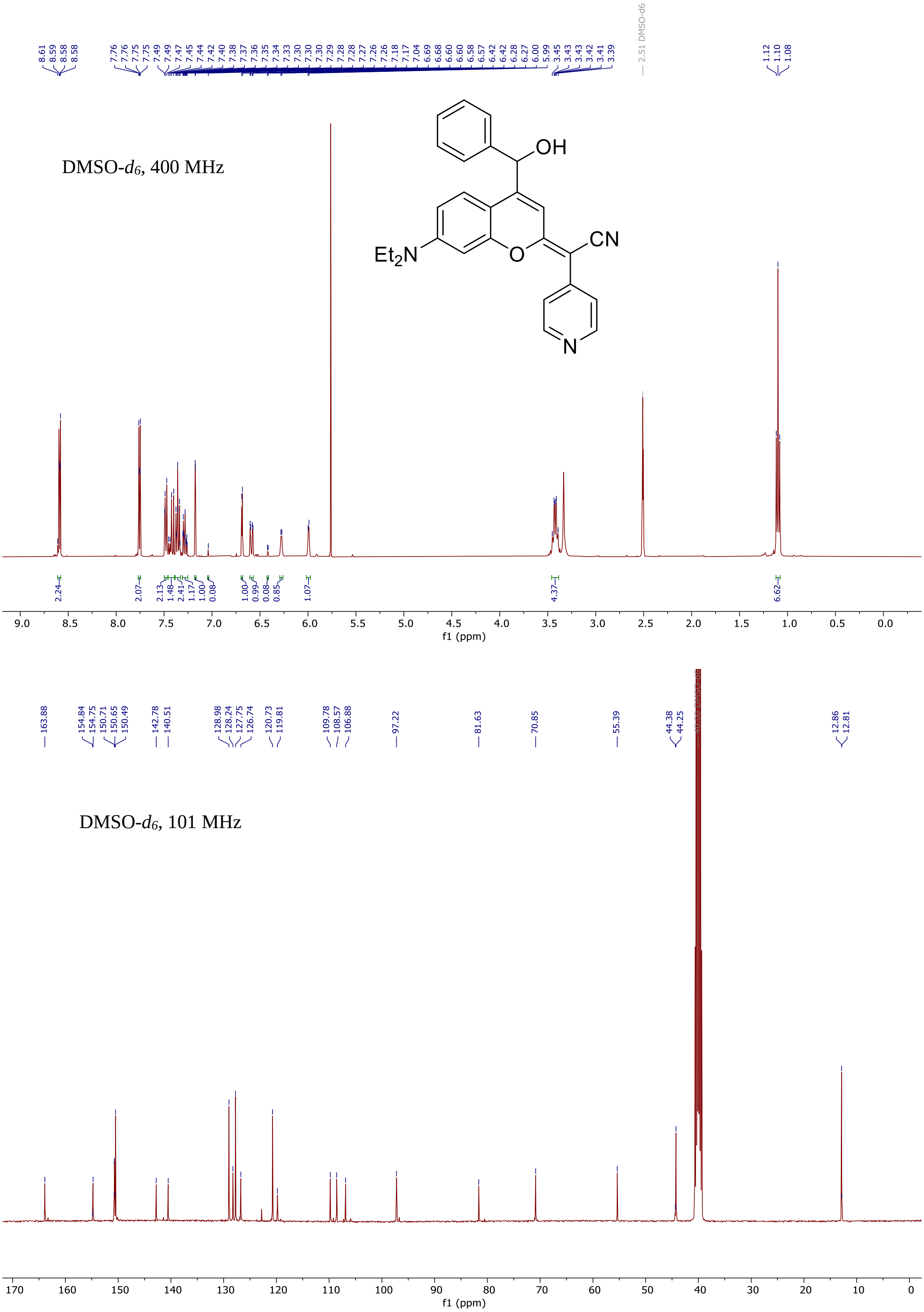

Figure S36. ^{1}H and ^{13}C NMR spectra of compound **15** in DMSO-d_6.

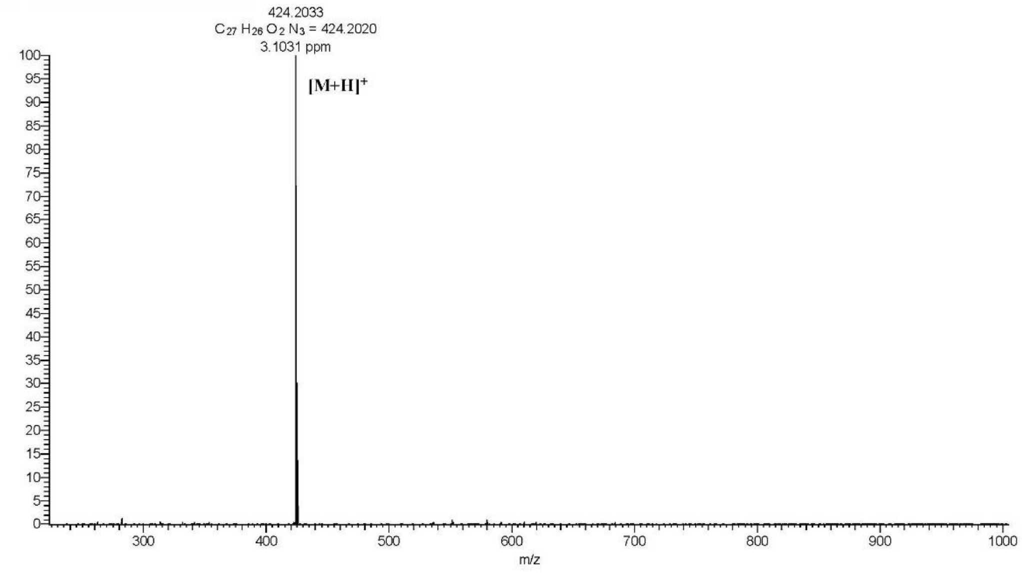

Figure S37. HR ESI-MS spectrum of compound **15**.

Compound 16

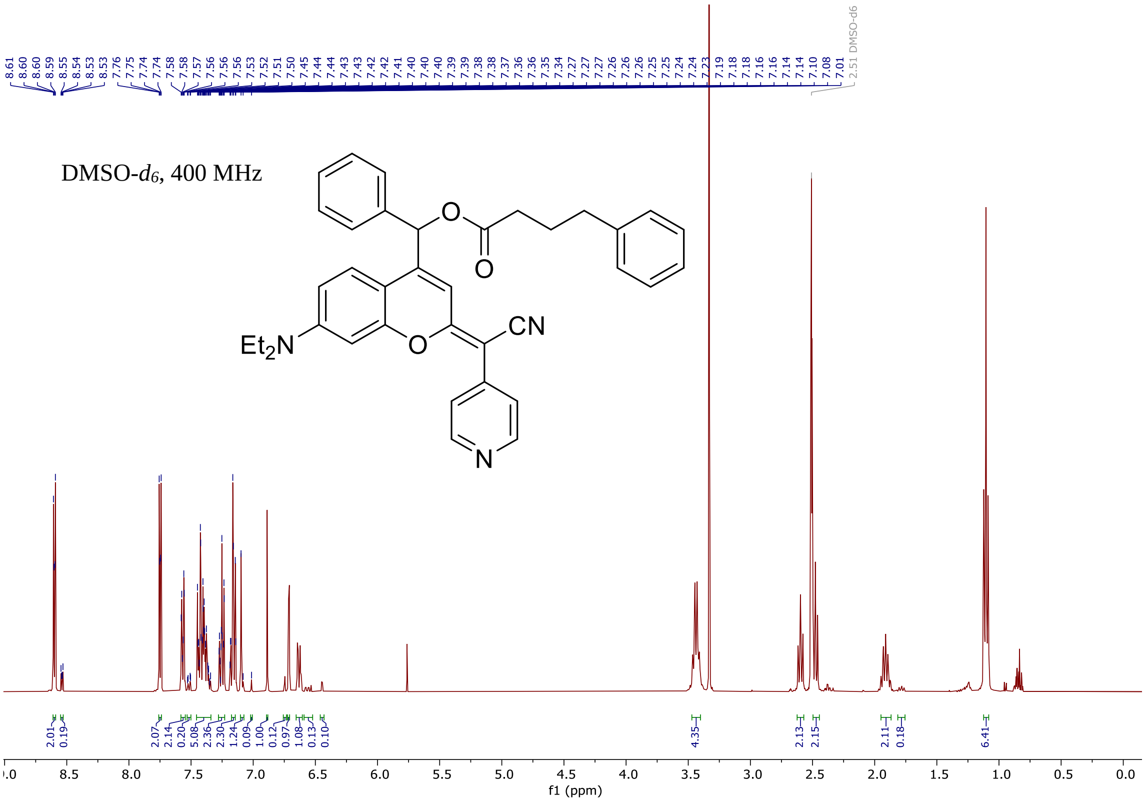

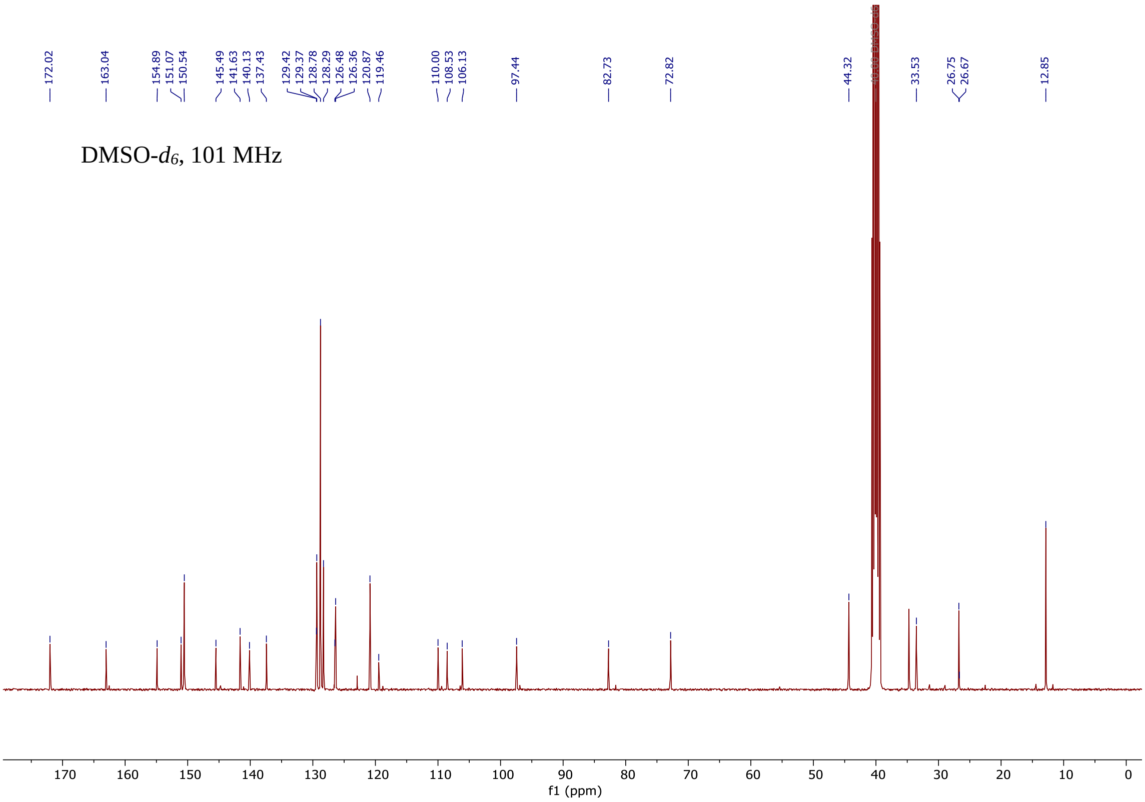

Figure S38. ^{1}H and ^{13}C NMR spectra of compound **16** in DMSO-d_6.

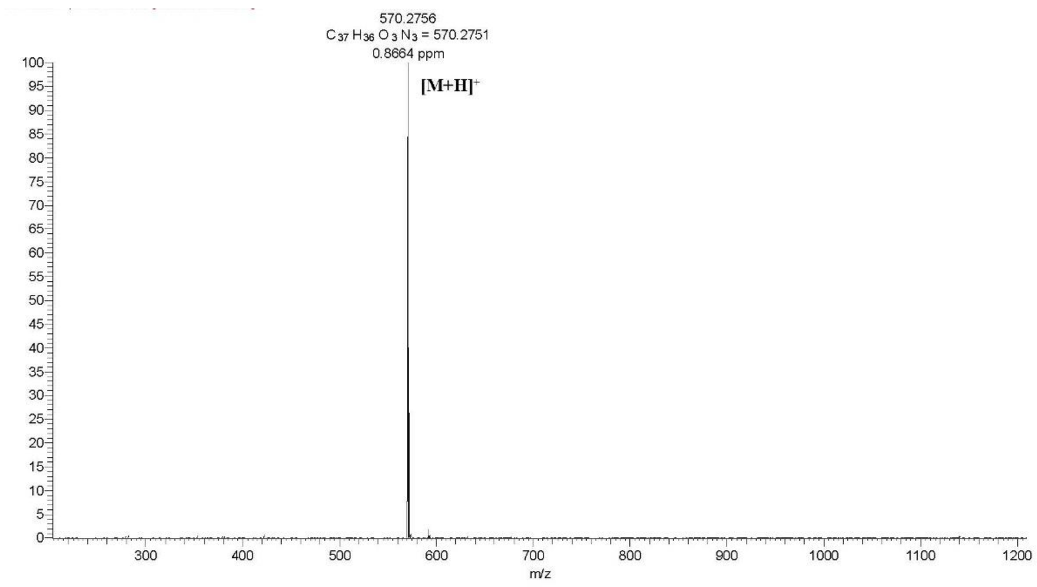

Figure S39. HR ESI-MS spectrum of compound **16**.

Compound 17

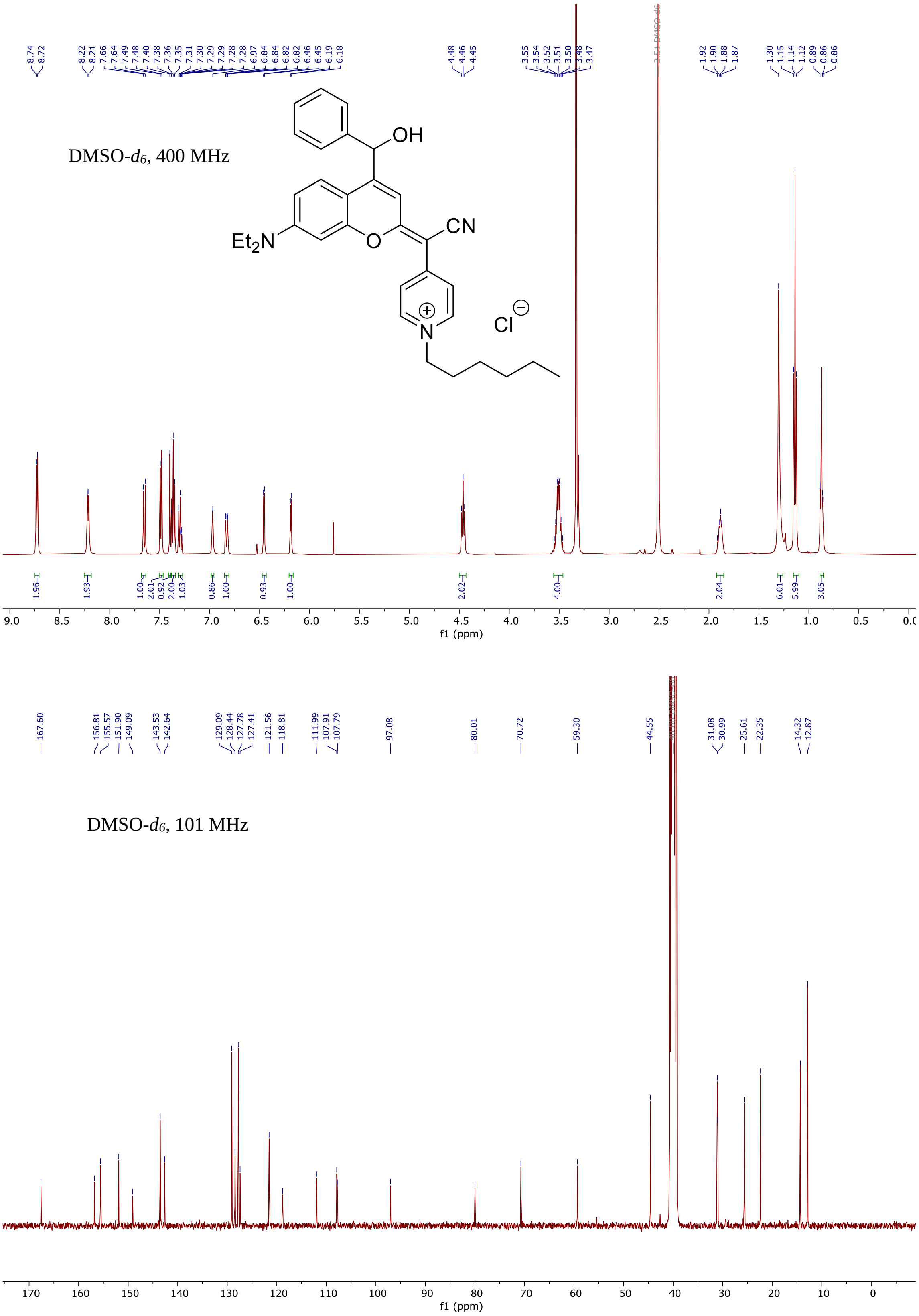

Figure S40. ^{1}H and ^{13}C NMR spectra of compound **17** in DMSO-d_6.

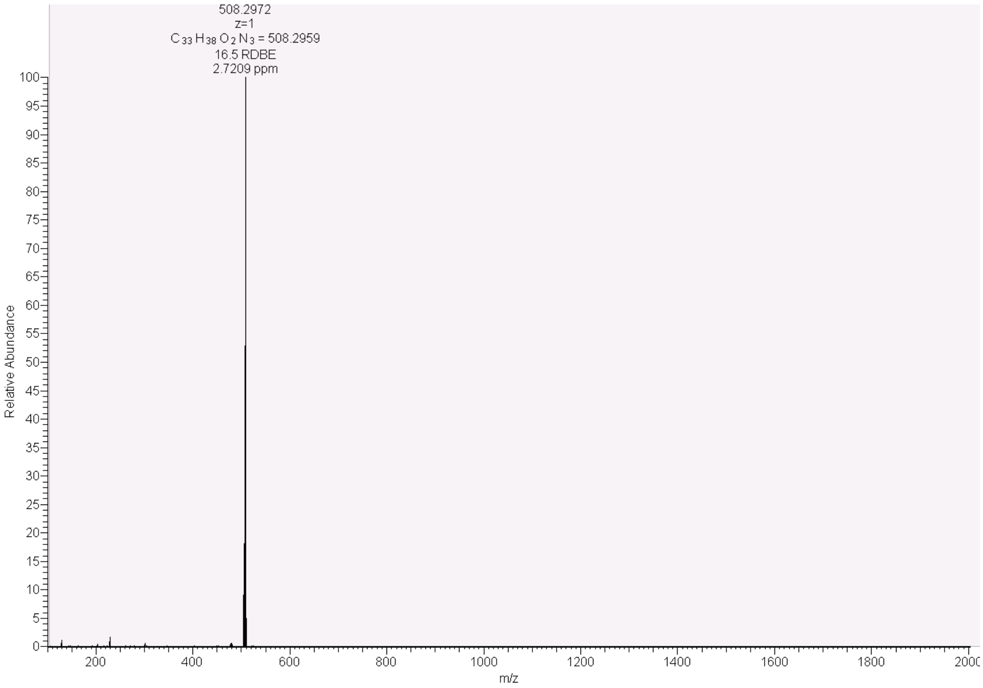

Figure S41. HR ESI-MS spectrum of compound **17**.

Compound 4Ph

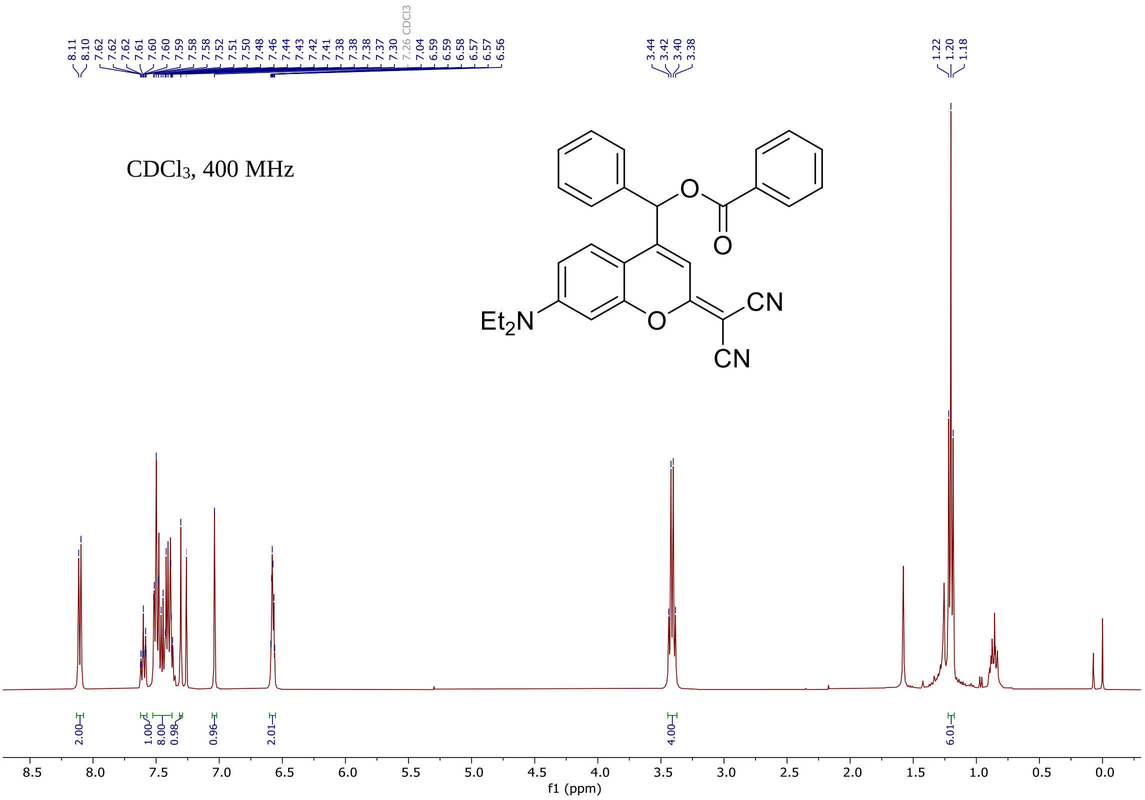

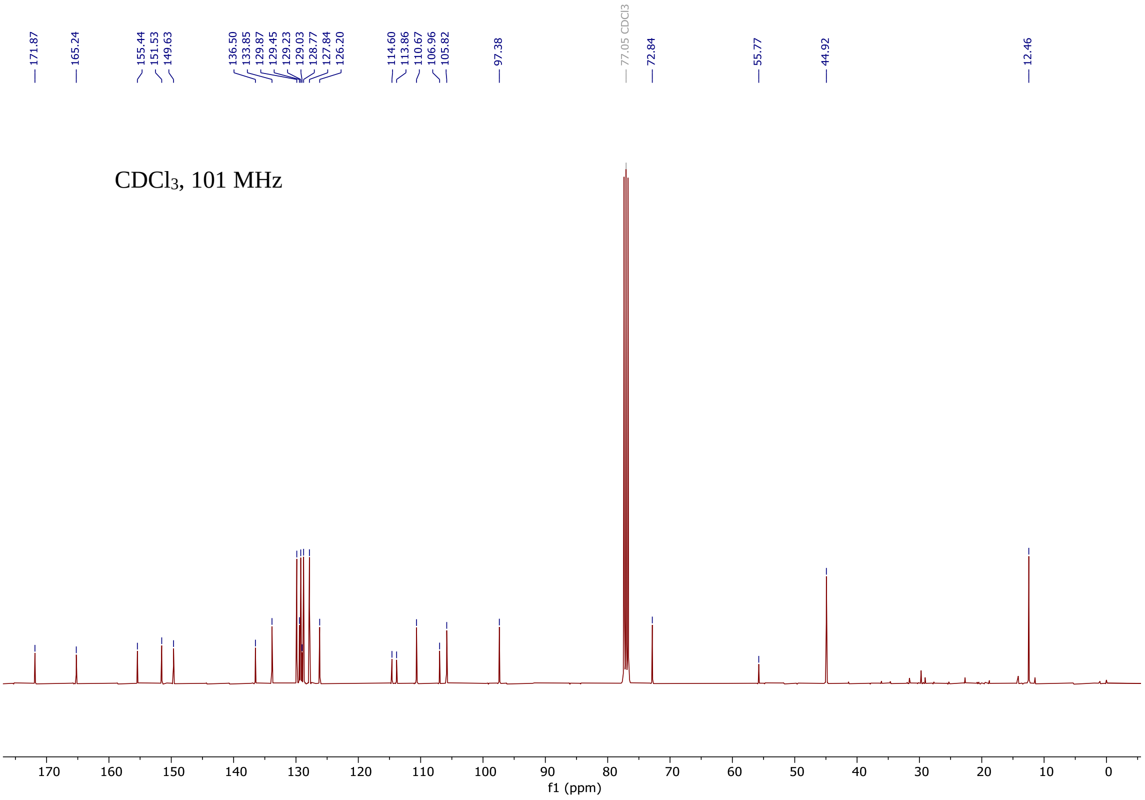

Figure S42. ^{1}H and ^{13}C NMR spectra of compound **4Ph** in $CDCl_3$.

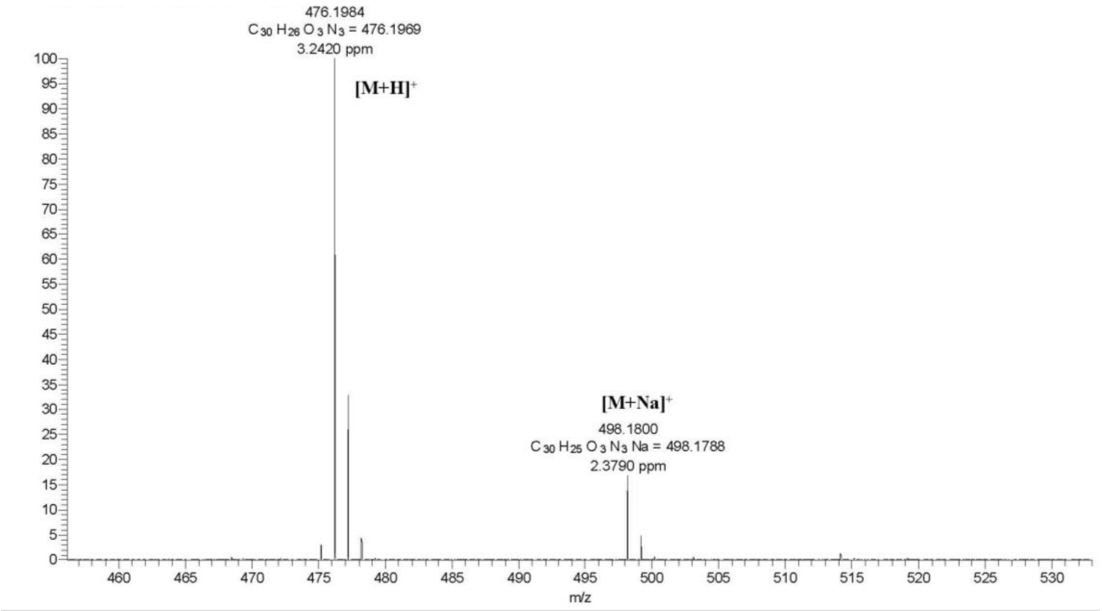

Figure S43. HR ESI-MS spectrum of compound **4Ph**.

Compound 5Ph

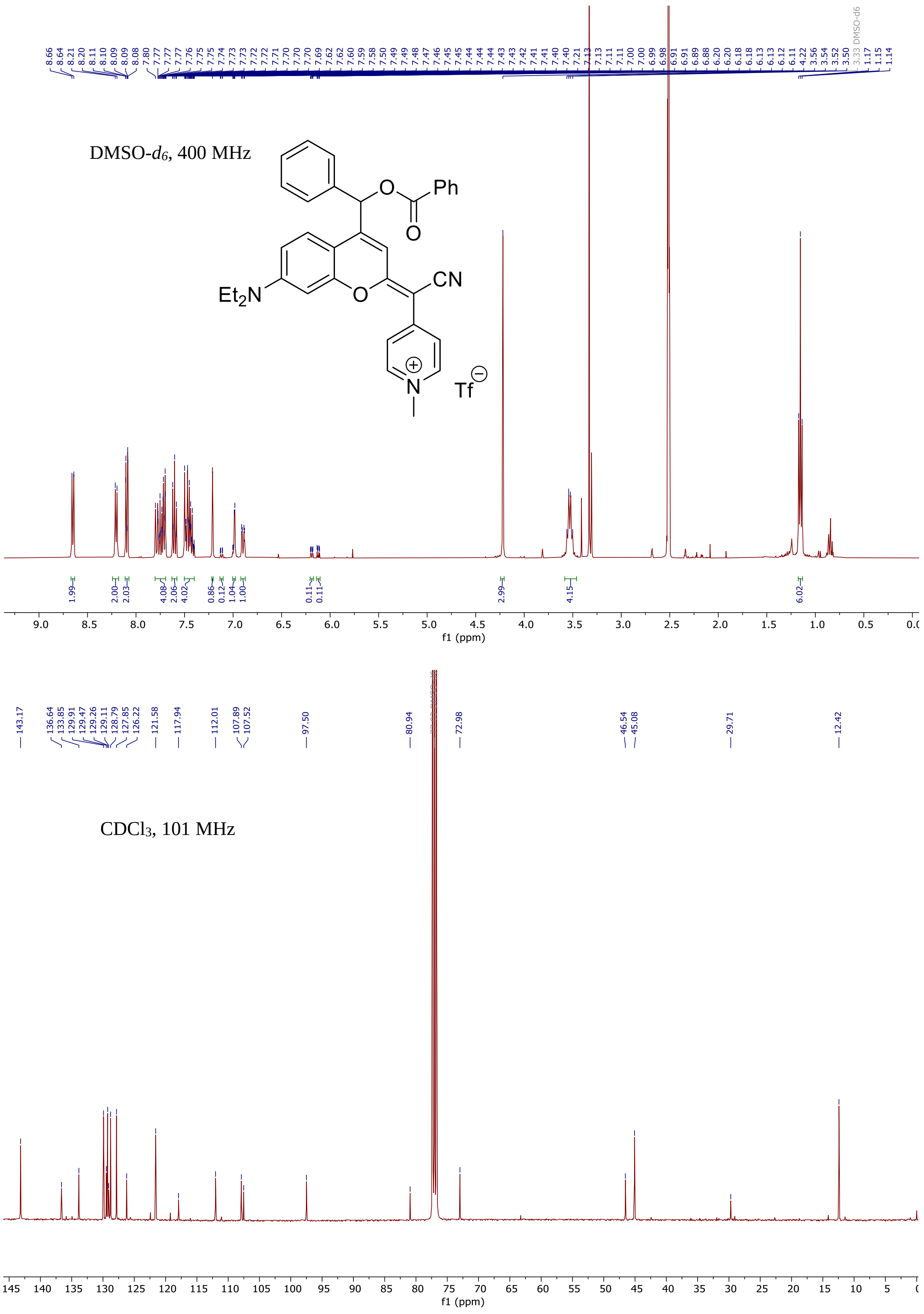

Figure S44. ^{1}H and ^{13}C NMR spectra of compound **5Ph** in DMSO-d_6 and $CDCl_3$, respectively.

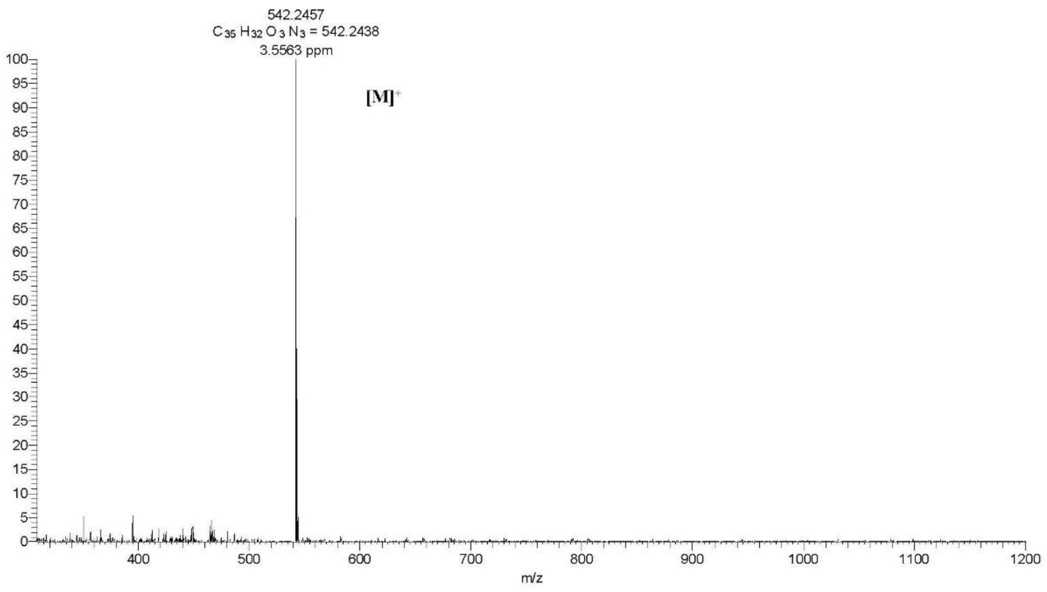

Figure S45. HR ESI-MS spectrum of compound **5Ph**.

Compound 6Ph

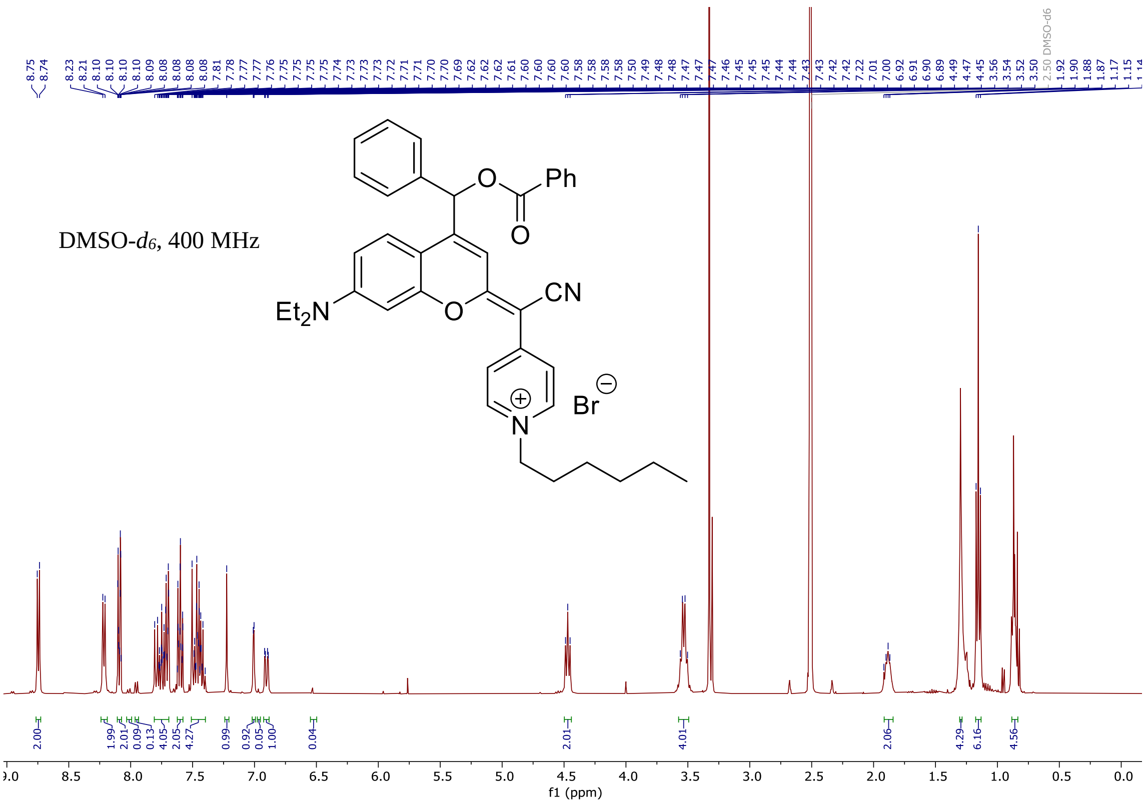

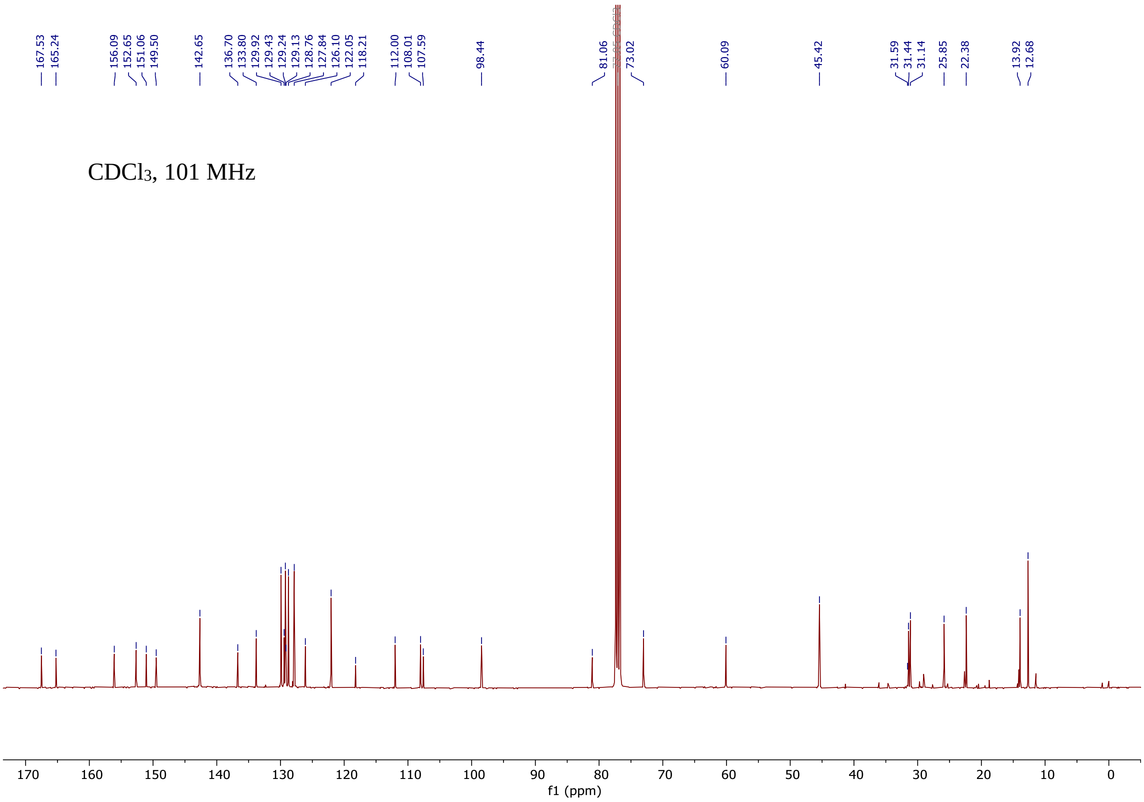

Figure S46. ^{1}H and ^{13}C NMR spectra of compound **6Ph** in DMSO-*d*$_6$ and $CDCl_3$, respectively.

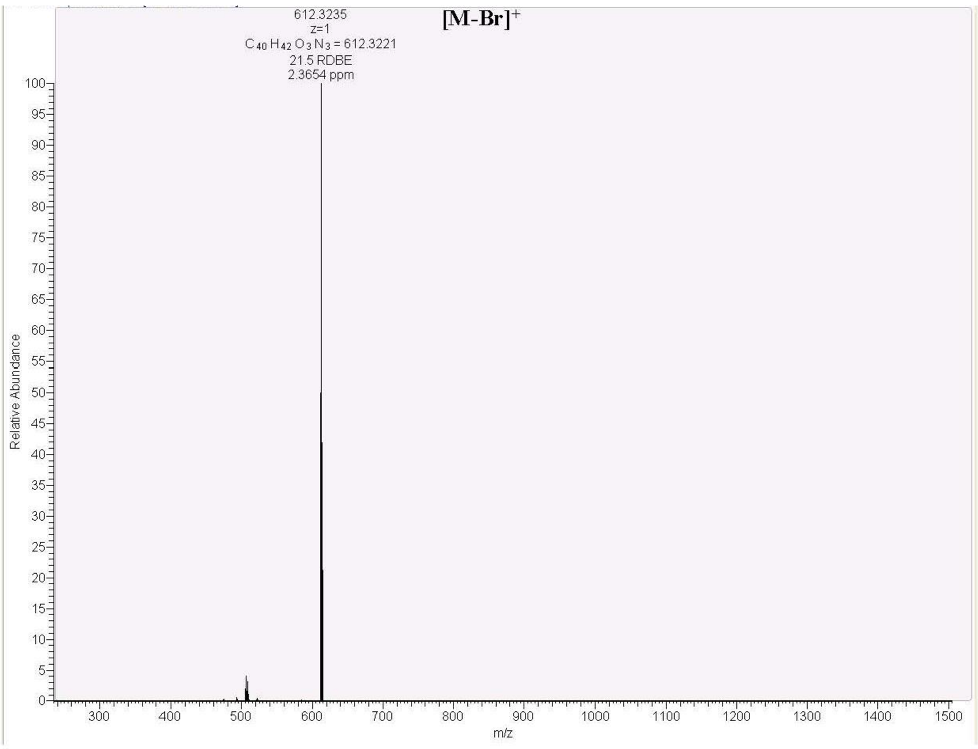

Figure S47. HR ESI-MS spectrum of compound **6Ph**.

Compound 7

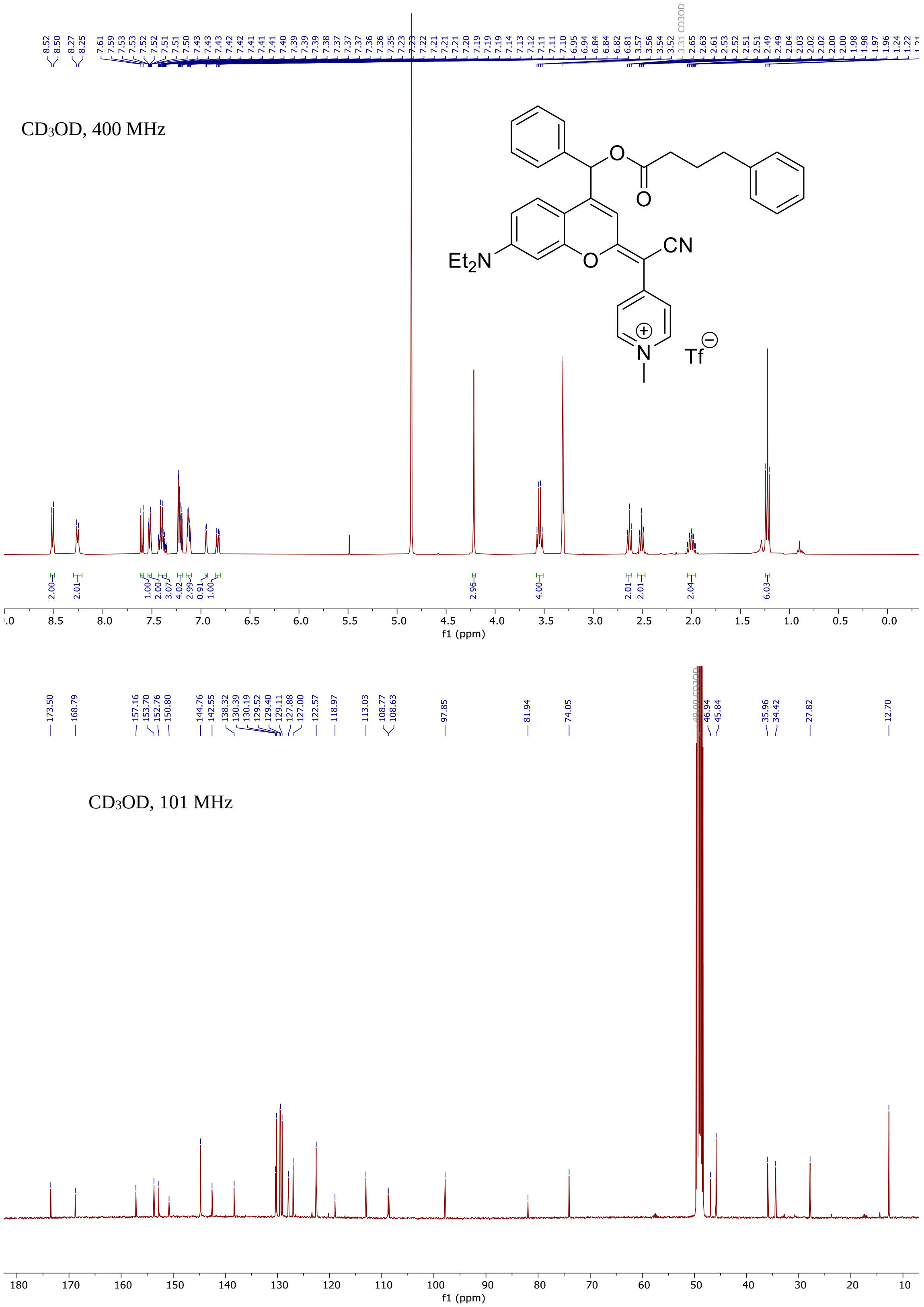

Figure S48. ^{1}H and ^{13}C NMR spectra of compound **7** in CD_3OD.

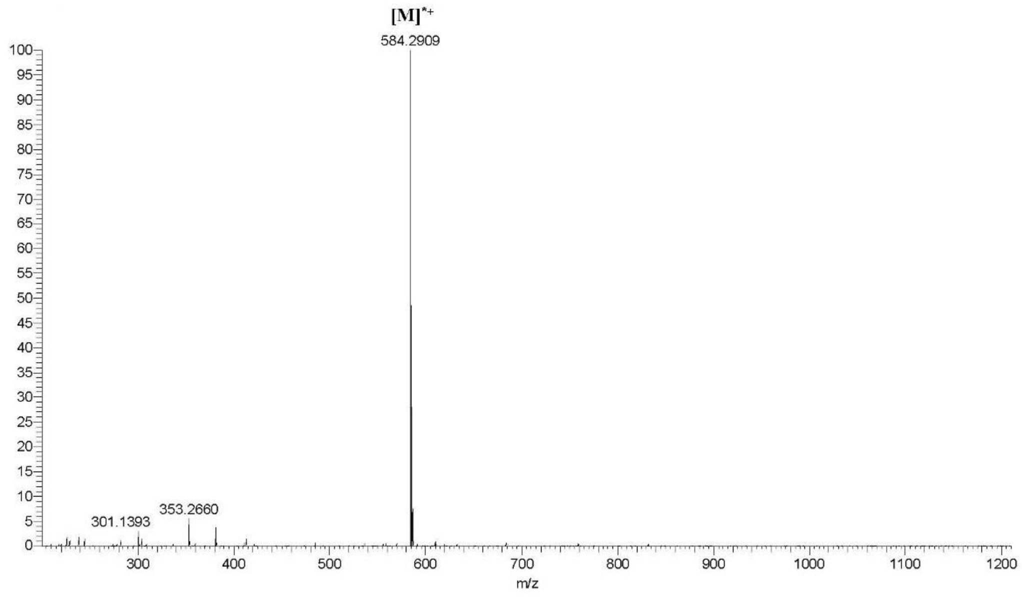

Figure S49. HR ESI-MS spectrum of compound **7**.

Compound 8

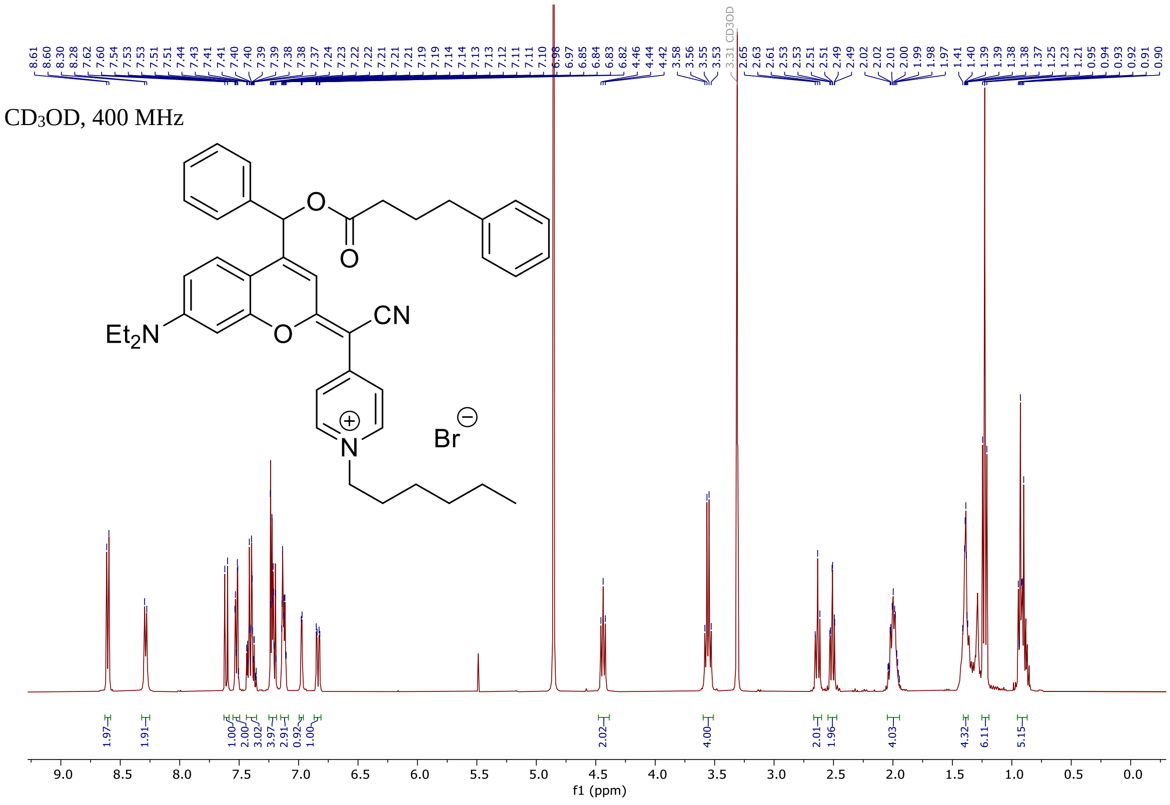

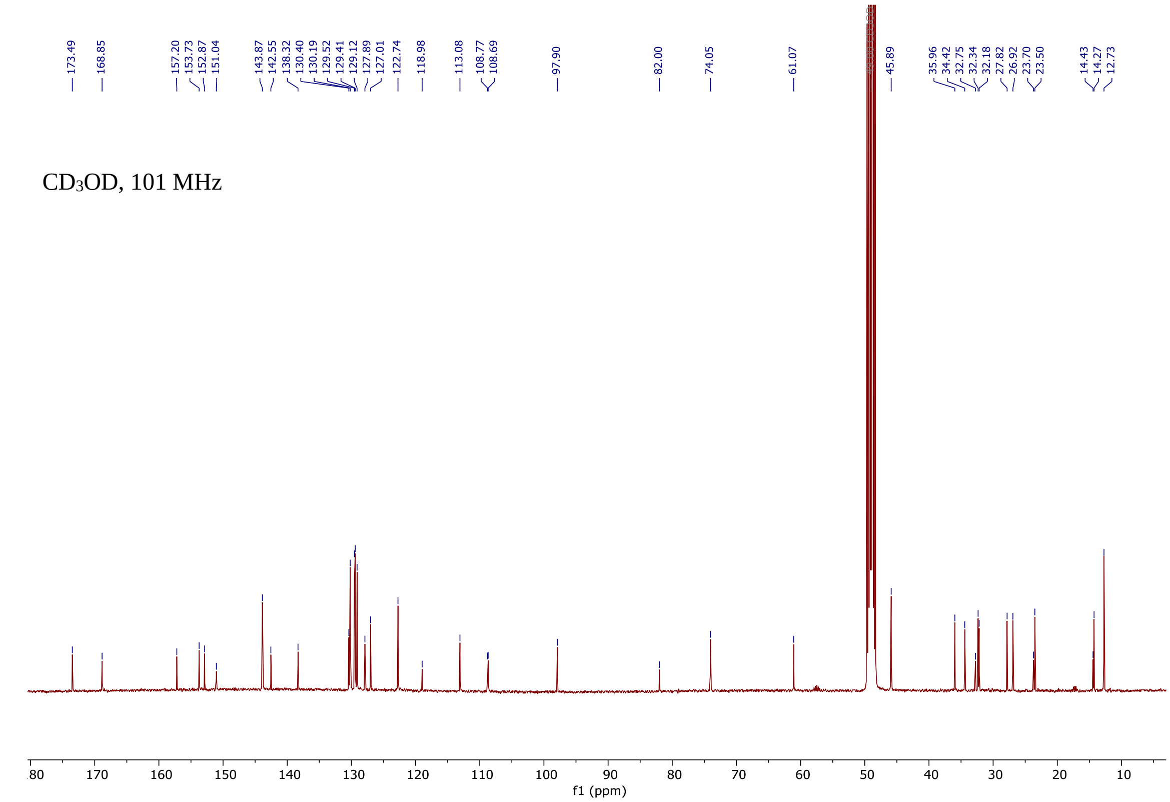

Figure S50. ^{1}H and ^{13}C NMR spectra of compound **8** in CD_3OD.

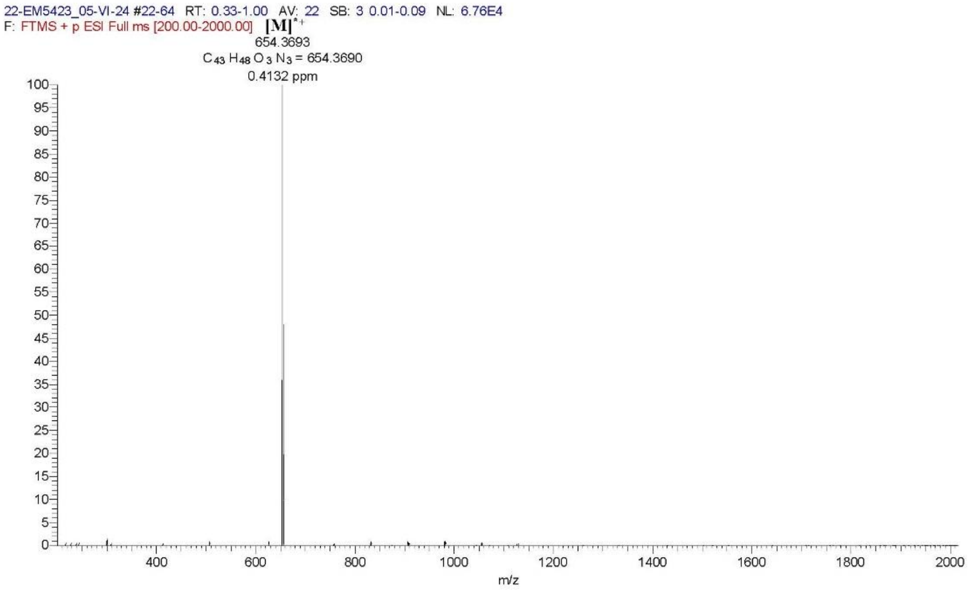

Figure S51. HR ESI-MS spectrum of compound **8**.

Compound 9

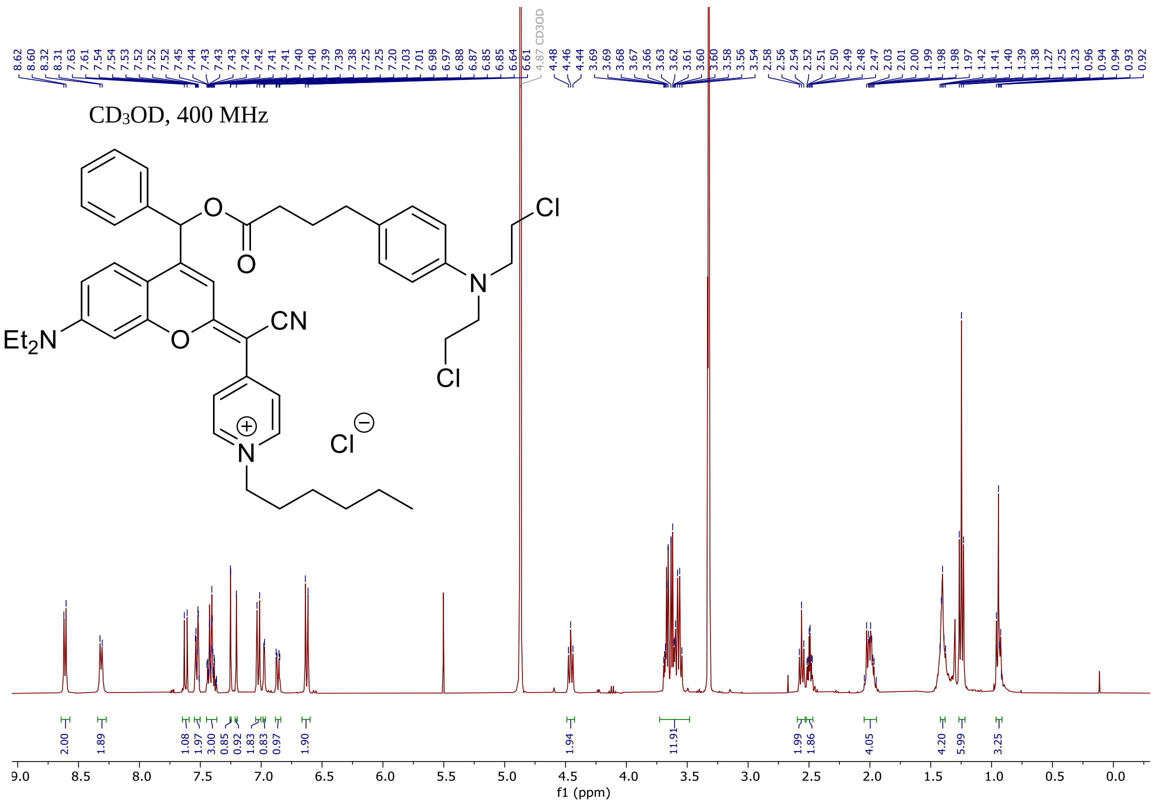

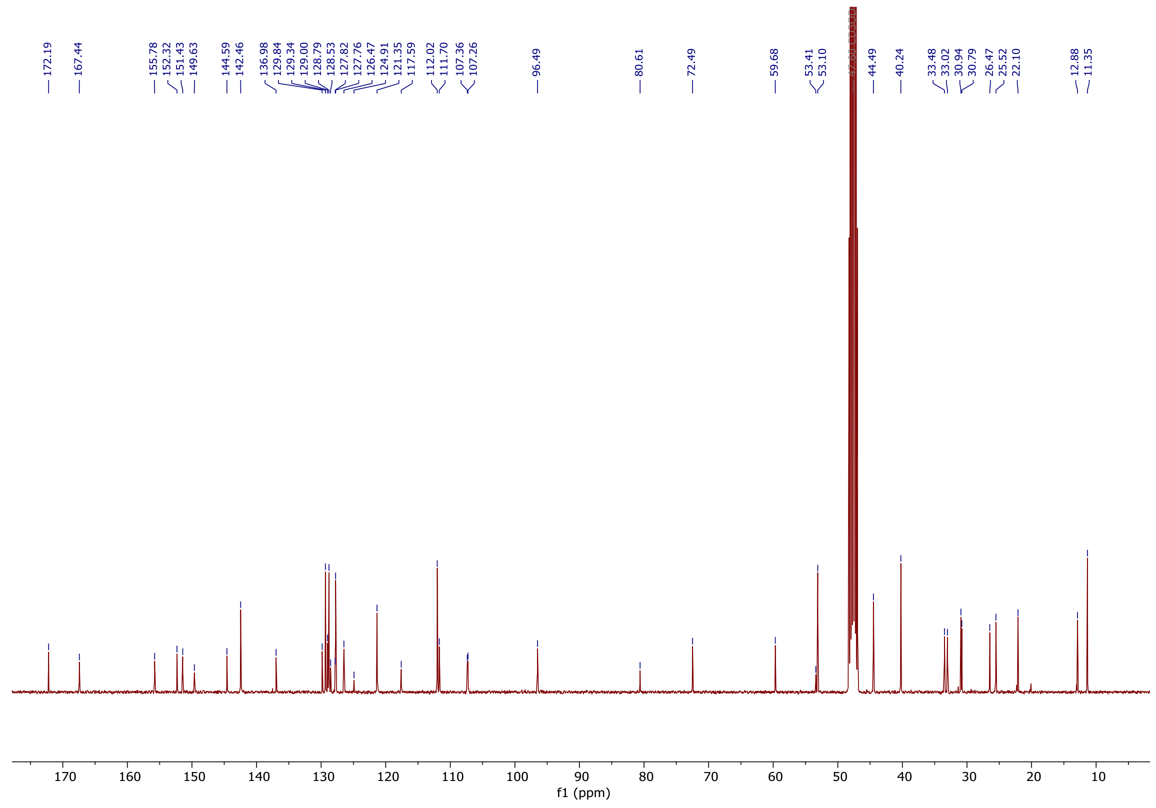

Figure S52. ^{1}H and ^{13}C NMR spectra of compound **9** in CD_3OD.

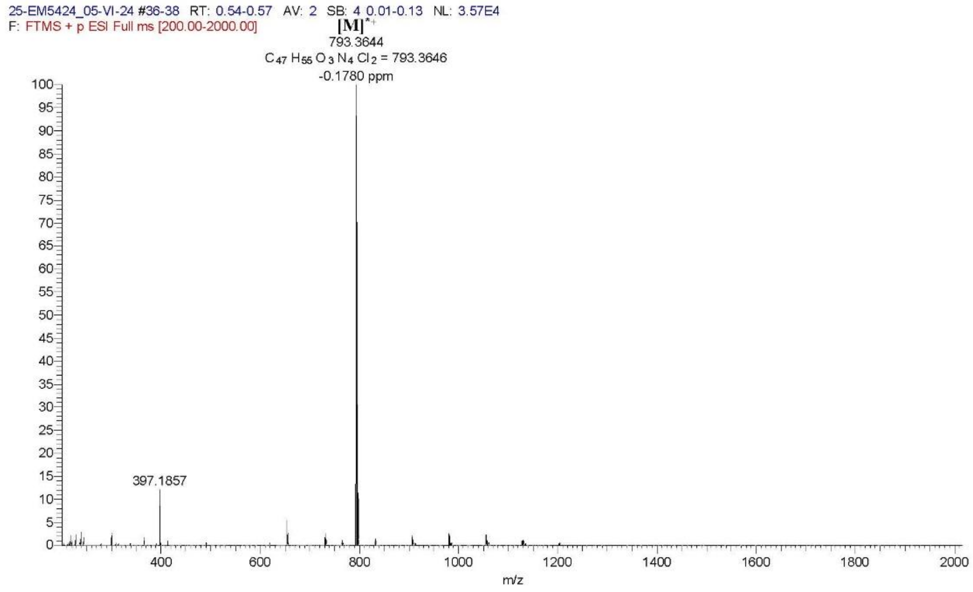

Figure S53. HR ESI-MS spectrum of compound **9**.

7. References

[39] Roibu, A.; Fransen, S.; Leblebici, M. E.; Meir, G.; Gerven, T. V.; Kuhn, S., An accessible visible-light actinometer for the determination of photon flux and optical pathlength in flow photo microreactors, *Sci. Rep.* **2018**, *8*, 5421.

[40] Maafi, M. The potential of AB(1Φ) systems for direct actinometry. Diarylethenes as successful actinometers for the visible range. *Phys. Chem. Chem. Phys*. **2010**, *12,* 13248–13254.

[41] Maafi, M.; Brown, R. G., The kinetic model for AB(1Φ) systems A closed-form integration of the differential equation with a variable photokinetic factor. *J. Photochem. Photobiol. A 187,* **2007**, 319–324.

[42] Sumi, T.; Takagi, Y.; Yagi, A.; Morimoto, M.; Irie, M. Photoirradiation wavelength dependence of cycloreversion quantum yields of diarylethenes. *Chem. Commun.* **2014***, 50,* 3928–3930.
